# Potential Molecular Mechanisms of Rare Anti-Tumor Immune Response by SARS-CoV-2 in Isolated Cases of Lymphomas

**DOI:** 10.3390/v13101927

**Published:** 2021-09-25

**Authors:** Debmalya Barh, Sandeep Tiwari, Lucas Gabriel Rodrigues Gomes, Marianna E. Weener, Khalid J. Alzahrani, Khalaf F. Alsharif, Alaa A. A. Aljabali, Murtaza M. Tambuwala, Kenneth Lundstrom, Sk. Sarif Hassan, Ángel Serrano-Aroca, Kazuo Takayama, Preetam Ghosh, Elrashdy M. Redwan, Bruno Silva Andrade, Siomar de Castro Soares, Vasco Azevedo, Vladimir N. Uversky

**Affiliations:** 1 Centre for Genomics and Applied Gene Technology, Institute of Integrative Omics and Applied Biotechnology (IIOAB), Nonakuri, Purba Medinipur 721172, West Bengal, India; 2 Department of Genetics, Ecology and Evolution, Institute of Biological Sciences, Federal University of Minas Gerais, Belo Horizonte 31270-901, Brazil; sandip_sbtbi@yahoo.com (S.T.); lucasgabriel388@gmail.com (L.G.R.G.); vascoariston@gmail.com (V.A.); 3 Clinical Research Center, Oftalmic, CRO, 119334 Bardina Str. 22/4, 119991 Moscow, Russia; marianna.weener@gmail.com; 4 Department of Clinical Laboratories Sciences, College of Applied Medical Sciences, Taif University, Taif 21944, Saudi Arabia; ak.jamaan@tu.edu.sa (K.J.A.); alsharif@tu.edu.sa (K.F.A.); 5 Department of Pharmaceutics and Pharmaceutical Technology, Faculty of Pharmacy, Yarmouk University, Irbid 21163, Jordan; alaaj@yu.edu.jo; 6 School of Pharmacy and Pharmaceutical Science, Ulster University, Coleraine BT52 1SA, UK; m.tambuwala@ulster.ac.uk; 7 PanTherapeutics, CH 1095 Lutry, Switzerland; lundstromkenneth@gmail.com; 8 Department of Mathematics, Pingla Thana Mahavidyalaya, Maligram, Paschim Medinipur 721140, West Bengal, India; sksarifhassan@pinglacollege.ac.in; 9 Biomaterials and Bioengineering Lab, Centro de Investigación Traslacional San Alberto Magno, Universidad Católica de Valencia San Vicente Mártir, 46001 Valencia, Spain; angel.serrano@ucv.es; 10 Center for iPS Cell Research and Application (CiRA), Kyoto University, Kyoto 606-8507, Japan; kazuo.takayama@cira.kyoto-u.ac.jp; 11 Department of Computer Science, Virginia Commonwealth University, Richmond, VA 23284, USA; preetam.ghosh@gmail.com; 12 Department of Biological Science, Faculty of Science, King Abdulazizi University, Jeddah 21589, Saudi Arabia; rredwan@gmail.com; 13 Laboratory of Bioinformatics and Computational Chemistry, Department of Biological Sciences, State University of Southwest Bahia (UESB), Jequié 45206-190, Brazil; bandrade@uesb.edu.br; 14 Department of Immunology, Microbiology and Parasitology, Institute of Biological and Natural Sciences, Federal University of Triângulo Mineiro (UFTM), Uberaba 38025-180, Brazil; siomars@gmail.com; 15 Department of Molecular Medicine and USF Health Byrd Alzheimer’s Institute, Morsani College of Medicine, University of South Florida, Tampa, FL 33612, USA; vuversky@usf.edu; 16 Research Center for Molecular Mechanisms of Aging and Age-Related Diseases, Moscow Institute of Physics and Technology, Institutskiy pereulok, 9, 141700 Dolgoprudny, Russia

**Keywords:** lymphoma, cancer, SARS-CoV-2, M protein, ORF3a, anti-tumor immunotherapy, gamma-tubulin ring complex, PD-1, monoclonal antibody

## Abstract

Recently, two cases of complete remission of classical Hodgkin lymphoma (cHL) and follicular lymphoma (FL) after SARS-CoV-2 infection were reported. However, the precise molecular mechanism of this rare event is yet to be understood. Here, we hypothesize a potential anti-tumor immune response of SARS-CoV-2 and based on a computational approach show that: (i) SARS-CoV-2 Spike-RBD may bind to the extracellular domains of CD15, CD27, CD45, and CD152 receptors of cHL or FL and may directly inhibit cell proliferation. (ii) Alternately, upon internalization after binding to these CD molecules, the SARS-CoV-2 membrane (M) protein and ORF3a may bind to gamma-tubulin complex component 3 (GCP3) at its tubulin gamma-1 chain (TUBG1) binding site. (iii) The M protein may also interact with TUBG1, blocking its binding to GCP3. (iv) Both the M and ORF3a proteins may render the GCP2-GCP3 lateral binding where the M protein possibly interacts with GCP2 at its GCP3 binding site and the ORF3a protein to GCP3 at its GCP2 interacting residues. (v) Interactions of the M and ORF3a proteins with these gamma-tubulin ring complex components potentially block the initial process of microtubule nucleation, leading to cell-cycle arrest and apoptosis. (vi) The Spike-RBD may also interact with and block PD-1 signaling similar to pembrolizumab and nivolumab- like monoclonal antibodies and may induce B-cell apoptosis and remission. (vii) Finally, the TRADD interacting “PVQLSY” motif of Epstein-Barr virus LMP-1, that is responsible for NF-kB mediated oncogenesis, potentially interacts with SARS-CoV-2 M^pro^, NSP7, NSP10, and spike (S) proteins, and may inhibit the LMP-1 mediated cell proliferation. Taken together, our results suggest a possible therapeutic potential of SARS-CoV-2 in lymphoproliferative disorders.

## 1. Introduction

In most cases, cancer is either reported to be a comorbid condition, or associated with COVID-19 disease severity from SARS-CoV-2 infection [1]. However, it was recently reported that a patient with classical Hodgkin lymphoma (cHL) showed disease remission upon infection with SARS-CoV-2 [2]. A similar observation has been reported in a case of follicular lymphoma (FL) [3]. The molecular profiles and the infecting SARS-CoV-2 strains of these patients are unknown, and the authors suggest that the complete remission of the diseases is due to an unknown anti-tumor immune response exerted by SARS-CoV-2 [1,2]. These findings have prompted us to elaborate on the molecular mechanisms behind the SARS-CoV-2 induced remission of cHL and FL.

To place the anti-tumor effect of the SARS-CoV-2 possessing a single-stranded RNA (ssRNA) genome in the right context, it is appropriate to mention that several other ssRNA viruses have demonstrated oncolytic activity, leading to efficient killing of tumor cells while causing no damage to normal tissue [4]. This oncolytic effect is not unique to RNA viruses as it has also been documented for DNA viruses, such as adenoviruses, herpes simplex viruses, and vaccinia viruses [5]. The oncolytic activity of viruses relates to the induction of high expression of protein kinase PKR, the interferon signaling pathway, and the activation of caspases inducing apoptotic cell death [6]. Alphaviruses, measles viruses, rhabdoviruses, and Newcastle disease viruses (NDV) have been used in preclinical animal models and in clinical trials, showing encouraging results for cancer therapy [7]. However, the oncolytic effects of viruses vary. For instance, significantly lower Zinc-finger antiviral protein (ZAP) expression in tumors prevented the viral RNA degradation and translational inhibition of the oncolytic M1 alphavirus commonly occurring in normal tissue [8]. In the case of measles viruses, it has been demonstrated that CD46 mediates virus attachment, entry, and virus-induced cell-to-cell fusion for the MV–Edmonston strain [9]. Typically, the high density of CD46 on tumor cells contributes to extensive cell fusion and enhancement of viral gene expression and provides a compelling explanation for the oncolytic specificity of the MV–Edmonston strain and its favorable use for CD46-targeted cancer therapy. Related to rhabdoviruses, genome-wide RNAi screening identified the endoplasmic reticulum (ER) stress response pathways as important modulators for sensitization to caspase-2-dependent apoptosis and cell death [10]. The tumor-cell-killing mechanisms of the NDVare based directly on the formation of multinucleated syncytia, activation of the extrinsic apoptotic pathway, activation of ER stress pathways, and involvement of MAPK pathways [11]. Indirectly, NDV can induce secretion of proinflammatory cytokines and chemokines, enhanced adhesion of leukocytes, upregulation of MHC, and cell adhesion molecules to activate tumor-specific lymphocytes.

The cHL neoplastic cell population is called Hodgkin or Reed–Sternberg (HRS) cells, which exclusively express CD30 and CD15, a cluster of differentiation (CD) markers. Additionally, CD40, CD80, CD86, and CD95 are expressed in most cHL cases, and CD20, CD45, and CD3 are rarely expressed by the HRS [12,13]. However, some reports suggest that CD20 is expressed at very low intensity in 5 to 80% of cases of cHL [14]. Other expressed proteins by the cHL (5–100% cases) are CD19, CD79a, CD138, paired box protein Pax-5 (PAX5), also known as B-cell-specific transcription factor (BSAP), and interferon regulatory factor (4IRF4), also known as multiple myeloma oncogene 1, (MUM1) [14]. Gamma-glutamyltransferase 1 (GGT1) could also be a potential marker for cHL [15], and cytotoxic T-lymphocyte associated protein 4 (CTLA-4) is expressed in programmed cell death-1 (PD-1) negative cHL [16]. On the other hand, CD19 is exclusively expressed, and CD20 is also found in some cases of FL [17].

cHL cells exclusively express CD30, and brentuximab vedotin (SGN-35 or Adcetris) is an FDA-approved CD30 targeted drug for the treatment of cHL [18]. SGN-35 is an anti-CD30 monoclonal antibody (mAb) attached to an anti-microtubule compound monomethyl auristatin E (MMAE). Upon binding to CD30, SGN-35 is internalized into cHL cells and releases MMAE that binds to tubulin and thus leads to cell-cycle arrest and apoptosis [18]. However, 20% to 30% of cHL cases were found to relapse [19]. Anti-CD20 mAbs such as rituximab are used to treat FL. Anti-CD19 and anti-CD47 mAbs may also be attractive targets for immunotherapy of FL [20].

Genomic amplification 9p24.1 in cHL has been associated with overexpression of programmed death ligand 1 (PD-L1) and activation of Janus kinase 1 and signal transducer and activator of transcription (JAK/STAT) signaling for disease relapse. Therefore, blocking PD-L1 and PD-L2 receptor PD-1 could be a better treatment option. More recently, T-cell checkpoint inhibitory mAbs such as nivolumab and pembrolizumab against PD-1 have been found to increase the overall survival in cHL [21,22]. Both pembrolizumab and nivolumab bind to the PD-L1 binding site of PD-1 and block the access of PD-L1 and PD-L2, thus preventing the relapse of cHL [21]. FL can also express PD-1, and therefore, PD-1 immune checkpoint blockers such as pembrolizumab can also be promising targeted therapeutics in FL [20].

It is well-established that Epstein–Barr virus (EBV) infection is associated with increased risk, pathogenesis, and immunocompromised conditions in the elderly with cHL [23,24]. EBV-positive FL is not rare, and EBV may play a specific role in disease progression and/or lymphomagenesis [25]. The latent oncogenic membrane protein 1 (LMP-1) of EBV activates NF-κB, JAK/STAT, and PI3K/AKT pathways, leading to apoptosis-prone germinal center (GC) B-cells and prevention of plasma cell differentiation [23]. The cytoplasmic signaling domain of LMP-1 recruits tumor necrosis factor receptor-associated factors (TRAFs) and tumor necrosis factor receptor type 1-associated DEATH domain protein (TRADD) to activate NF-kB signaling-mediated B-cell proliferation [26].

The patient was EBV positive in the cHL case report, although no genetic or molecular profiles were provided [2]. Neither have any information in the case of FL been reported [3]. Therefore, it is difficult to evaluate the underlying molecular mechanism of the disease remission due to SARS-CoV-2 infection. Hence, we considered all possible genetic makeups of these patients. In our first hypothesis (i), we assume that SARS-CoV-2 brings remission in cHL/FL cells using a similar mechanism as brentuximab vedotin (SGN-35 or Adcetris) does. In this process, we presumed that the SARS-CoV-2 might bind to the extracellular domain of any cHL-specific cell surface CD marker using its Spike-RBD to attach and enter into lymphoma cells. Upon internalization, some protein of SARS-CoV-2 may interact with cell division-related proteins to bring apoptosis or cell-cycle arrest or termination of cell division. The second possibility/hypothesis (ii) is that as the patient was positive for EBV, some SARS-CoV-2 protein may interact with the LMP-1 of EBV upon internalization of SARS-CoV-2 into the cell to inhibit the interaction of LMP-1- TRAFs and/or TRADD from abolishing the LMP-1 mediated cell proliferation or cell de-differentiation. In the third hypothesis (iii), we presume that any protein of SARS-CoV-2 may bind to PD-1, similar to the targeted mAb drugs pembrolizumab or nivolumab, and induce remission of cHL/FL.

## 2. Materials and Methods

### 2.1. Protein Structures and 3D Modeling

Crystal structures used in this study are provided in Table S1. Since the SARS-CoV-2 strains of the infected lymphoma patients are unknown, we have used SARS-CoV-2 proteins of the original Wuhan, China strain (GenBank: NC_045512). The crystal structures of human CD30/TNFRSF8 (NP_001234.3), CD15/FUT4 (NP_002024.1), SARS-CoV-2 membrane (M) protein (YP_009724393.1), and LMP-1 (YP_401722.1) of EBV are unavailable. It is likely that the inability to crystallize the CD30, CD15, and LMP-1 of EBV is determined not only by their transmembrane nature but is also associated with high levels of intrinsic disorder predicted in these proteins (Supplementary File S1). Therefore, we used I-TASSER [27] and RaptorX web servers [28] to model these proteins. The models were further refined using GalaxyRefine [29], and the stereochemical quality of the protein structures were determined using the PROCHECK tool available at the SAVES v6.0 server (https://saves.mbi.ucla.edu/ (accessed on10 May 2021)). Finally, models were selected for further analysis based on residues in the most favored region of the Ramachandran plot. The TMHMM Server v.2.0 (http://www.cbs.dtu.dk/services/TMHMM/ (accessed on 10 May 2021)) was used to determine the transmembrane helices and extracellular sequences of all proteins.

### 2.2. Protein–Protein Docking

We performed protein–protein docking using ZDOCK [30] and HDOCK [31] servers. We identified the binding sites/residues from crystal structures using Ligplot+ v.2.2 [32] and from the literature in most cases. However, when we did not have access to such residue knowledge, we performed blind docking. The top ten complexes from ZDOCK or HDOCK were used for further analysis, selecting specific criteria as per the specific objective of the docking (see: Section 3). UCSF Chimera [33] was used to visualize and analyze the crystal structures and the docked complexes.

## 3. Results

### 3.1. 3D Protein Models

**Figure 1:**
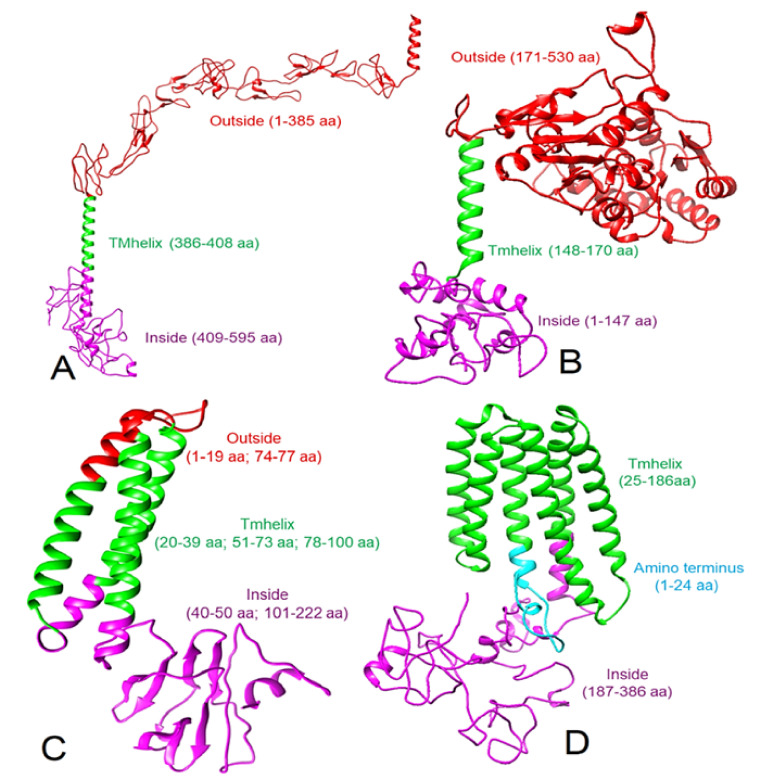
Modeled 3D structures (ribbon view) of proteins and their topology. (**A**) Human CD30, (**B**) human CD15, (**C**) SARS-CoV-2 M protein, and (**D**) Epstein-Barr virus LMP-1 protein. Note: the red color shows the extracellular location and corresponding amino acid residues, green shows the transmembrane location and corresponding amino acid residues, and purple color indicates the intracellular or cytoplasmic location and corresponding amino acid residues.

Compared to I-TASSER [27], a better model was obtained using RaptorX [28] for human CD30, human CD15, and the SARS-CoV-2 M protein. The RaptorX derived CD30 model was similar to previously described models [34,35]. Therefore, we used this structure of CD30 for protein–protein docking after refinement using GalaxyRefine [29]. The Ramachandran plot of the refined CD30 structure (Figure 1A) showed that 87.0% of the residues are in the most favorable regions (Table 1, Figure S1). TMHMM analysis suggested that residues 1 to 385 of the CD30 are exposed to the exterior of the cell and therefore, this area was used in docking experiments. For CD15, the final modeled structure (Figure 1B) showed a Ramachandran plot with 92.0% residues in the most favorable regions (Table 1, Figure S2). Residues 171 to 530 of CD15 were found on the exterior of the cell as per TMHMM analysis. RaptorX-derived SARS-CoV-2 M protein when refined with GalaxyRefine (Figure 1C), showed a Ramachandran plot with 92.5% of residues in the most favorable regions (Table 1, Figure S3) and as per TMHMM, the residues 1-19 and 74-77 of the M protein were predicted to be extracellular. While we modeled the LMP-1 of the EBV following the same method, 96% of residues were found in the most favorable regions of the Ramachandran plot (Figures S4 and S5). However, this refined structure showed five transmembrane helices. According to a previous report [26], the LMP-1 has six transmembrane helices, which we obtained directly from the RaptorX. Therefore, we did not use the GalaxyRefine derived structure of LMP-1 (Figures S4 and S5) for further analysis and docking but used the RaptorX-derived LMP-1 model, containing six transmembrane helices (Figure 1D), which showed 87.7% of residues in the most favorable regions of the Ramachandran plot (Table 1, Figure S6). The TMHMM algorithm did not generate any result for LMP-1. Therefore, we considered the membrane topology as described by Kieser, 2007 [26]. All these selected 3D models were used for protein–protein docking.

### 3.2. Spike-RBD May Bind to CD15, CD27, CD45, and CD152 Receptors of cHL or FL

The first step of SARS-CoV-2 infection is the attachment of the virus to human cells at the human angiotensin-converting enzyme 2 (*h*ACE2) through its S protein receptor-binding domain (RBD), which then facilitates cell membrane fusion for entry into human cells [36,37]. To evaluate our first hypothesis if the S protein binds to any cell surface receptor of cHL/FL, we performed protein–protein docking between the Spike-RBD and reported lymphoma cell surface markers (CD15, CD20, CD27, CD30, CD40, CD45, CD80, CD86, CD95, and CTLA-4/ CD152) using the ZDOCK server [30].

In general, a receptor-binding site of a protein should be in open conformation to bind to its ligand. An in silico structural analysis also predicts that a “1up2down” conformation of the Spike-RBD (one open and two closed monomers of the S protein homotrimer) probably is required prior to binding to the *h*ACE2 [38]. Similarly, the critical residues of the Spike-RBD that interact with the *h*ACE2 are Lys417, Tyr453, Gln474, Ph e486, Gln498, Thr500, and Asn501 [39]. However, any peptide that inhibits the interaction of the Spike-RBD and the *h*ACE2 must bind to three key positions of the Spike-RBD (Gly485/Phe486/Asn487, Gln493, and Gln498/Thr500/Asn501) [40]. To verify and standardize the ZDOCK- or HDOCK-based Spike-RBD and human CD receptor docking, we used the crystal structure of the Spike-RBD- *h*ACE2 complex (PDB: 6LZG, the structure of novel coronavirus spike receptor-binding domain complexed with its receptor *h*ACE2, Method: X-Ray diffraction, Resolution: 2.50 Å) and characterized the interacting residues using Ligplot+ v.2.2. The results showed that the Spike-RBD interacts with the *h*ACE2 through Lys417, Tyr449, Ala475, Asn487, Gly496, Gln498, Thr500, and Gly502 residues forming 10 H-bonds (Figure S7). While we combined these results and our additional structure-based analysis, we observed that four regions of the Spike-RBD—(i) R1—Lys417, Tyr449, Tyr453, and its adjacent residues; (ii) R2—Gln498–Tyr502; (iii) R3—Gln474, Ala475, Phe486, Asn487; (iv) R4—Gly496, Gln493—residues could be critical for strong interaction with any receptor. R2 and R3 are probably the most essential regions (Figure S8).

**Figure 2:**
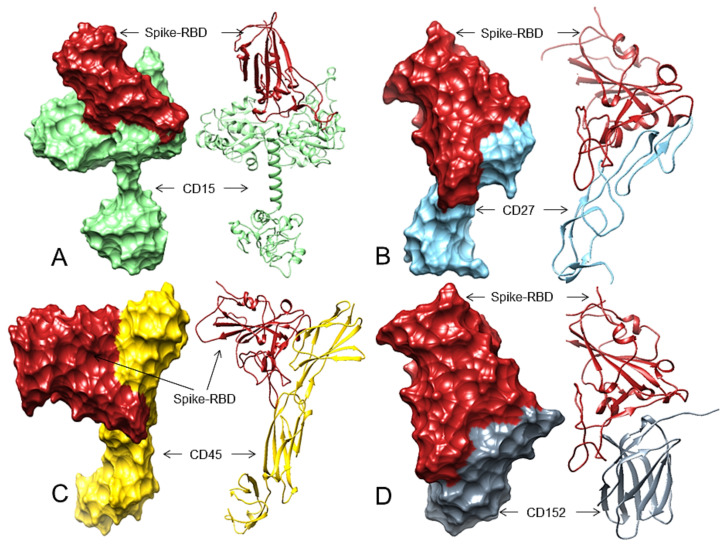
The 3D structures (surface and ribbon views) of human CDs and the Spike-RBD interactions. For interacting residues, see Table 2 and the corresponding supplement 2D Figure(s). (**A**) The Spike-RBD (fire brick) binding to human CD15 (light green) (corresponding to complex 3, Figure S9, 13 H-bonds, involving four regions of the Spike-RBD; (**B**) The Spike-RBD (fire brick) binding to human CD27 (sky blue) (corresponding to complex 9, Figure S13, 10 H-bonds, involving four regions of the Spike-RBD, (**C**) The Spike-RBD (fire brick) binding to human CD45 (gold) and the Spike-RBD (corresponding to complex 8, Figure S19, eight H-bonds, involving four regions of the Spike-RBD, and (**D**) The Spike-RBD (fire brick) binding to human CD152 (slate grey) and the Spike-RBD (corresponding to complex 2, Figure S26, 11 H-bonds, involving four regions of the Spike-RBD).

According to the ZDOCK results, the cell-exposed domain of CD15 (171-530 aa) potentially interacts with the spike-RBD residues, creating 13 H-bonds in complex 3, involving its predicted four regions (R1—Tyr449, Asn450, Tyr453; R2—Gln498, Thr500; R3—Asn487; R4—Ser494). In complex 4, the RBD binds to CD15, forming 11 H-bonds covering all four regions (R1—Tyr449; R2—Gln498, Asn501, R3—Tyr473, Asn487; R4—Gln493, Gly496). In complex 6, the RBD also interacts with CD15 involving four regions (R1—Lys417, Tyr449; R2—Gly502; R3—Glu484; R4—Gly496) and forming nine H-bonds (Table 2 and Table S2A, Figure 2A, Figures S9–S11). ZDOCK did not provide any good result for CD20 (CD antigen 20 or membrane-spanning 4-domains subfamily A member 1, MS4A1) and the Spike-RBD docking (Table S2B), and since the IRF4/MUM1 is a nuclear protein, we excluded this cHL marker from this analysis. The Spike-RBD interacts with CD antigen 30/tumor necrosis factor receptor superfamily member 7 (CD27/TNFRSF7, extracellular domain 20–191 aa) using four binding regions in complex 8 (R1—Lys417, Tyr449, Tyr453; R2—Gln498; R3—Ala475; R4—Gln493) and in complex 9 (R1—Lys417, Tyr453; R2—Gln498, Thr500; R3—Asn487; R4—Gln493) (Table 2, Figure 2B, Figures S12 and S13). Complex 8 and complex 9 form 10 H-bonds (Table S2C). A maximum 11 H-bonds may be formed between the Spike-RBD and the cell-surface-exposed (1 to 385aa) residues of the human CD30 (complex-5). In this complex, the Spike-RBD may bind to CD 30 in two regions (R1—Tyr453; R4—Gln493), and in complex 3, it binds again in two regions (R1—Lys417; R2—Thr500) but forms a total of 5 H-bonds with three other residues (Figures S14 and S15, Table S2D). Therefore, CD30 may not be a good candidate to act as a receptor for the S-RBD. The S-RBD possibly interacts with maximum 10 H-bonds in two complexes with the extracellular domain of CD antigen 40/ tumor necrosis factor receptor superfamily member 5 (CD40/TNFRSF5, residues 21-193). In complex 8, it covers three regions (R1—Lys417, Tyr449, Tyr453; R2—Gln498; R3—Ala475), and in complex 9, it interacts with four regions (R1—Lys417; R2—Gln498, Thr500; R3—Asn487; R4—Gln493) (Figures S16 and S17, Table S2E). Although in complex 9 the Spike-RBD used all four regions to interact with CD40, we did not see any other complex involving all the four regions. Therefore, CD40 also may not be a good receptor for the SARS-CoV-2 S protein. In ZDOCK docking, the Spike-RBD interacts with the CD45 (CD antigen 45 or receptor-type tyrosine-protein phosphatase C, PTPRC) extracellular domain (26- 577 aa) with a maximum nine H-bonds plus two salt bridges in complex-3, where the Spike-RBD involves four binding regions, each with one residue (R1—Lys417; R2—Gln498; R3—Asn487; R4—Gln493). In complex 8, the Spike-RBD also interacts using four regions forming six H-bonds plus two salt bridges and using six residues (R1—Lys417, Tyr453; R2—Gln498, Asn501, R3—Asn487; R4—Gln493) (Table 2 and Table S2F, Figure 2C, Figures S18 and S19). Therefore, CD45 could be a good candidate for the Spike-RBD interaction. For CD80 (CD antigen 80 or T-lymphocyte activation antigen CD80) extracellular domain (35-242 aa), the Spike-RBD forms a total of nine H-bonds in complex 9 and uses three regions (R1—Lys417, Tyr449; R2—Gln498; R4—Gly496) and in complex 3 (total eight H-bonds) it also involves three regions (R2—Asn501; R3—Ala475; R4—Gln493) (Figures S20 and S21, Table S2G). Although complex 9 involves all four regions, they only make four H-bonds. Therefore, the Spike-RBD may not also interact with CD 80. When we docked the Spike-RBD with the extracellular domain of CD86 (CD antigen 86 or T-lymphocyte activation antigen CD86), (24-247 aa), we found that the Spike-RBD may bind through only three regions (R1—Lys417, Tyr453; R2—Asn501, R4—Gly496) in complex 2 that forms a total of 10 H-bonds. In complex 9, the Spike-RBD forms eight H-bonds; however, it uses three regions (R1—Tyr449; R2—Gln498, Thr500; R4—Gln493). Therefore, CD86 also may not be a good candidate that can act as a receptor for SARS-CoV-2 attachment to human cells (Figures S22 and S23, Table S2H). In CD95 (CD antigen 95 or tumor necrosis factor receptor superfamily member 6 or Apoptosis-mediating surface antigen FAS) extracellular domain (26-173 aa) and the Spike-RBD docking, we observed two complexes that show good interactions involving three regions. In complex 2, the Spike-RBD involves R1—Lys417, Tyr449; R3—Asn487; R4—Gln493, and in complex 10, the Spike-RBD uses R1—Thr449; R2—Gln498, Thr500; R3—Asn487 (Figures S24 and S25, Table S2I). The Spike-RBD interacts with our last tested receptor CTLA4/CD152 (cytotoxic T-lymphocyte protein 4 or CD antigen 125) extracellular domain (36-161 aa) using all four regions in complex 2, where the Spike-RBD interacts using residues (R1—Tyr449; R2—Gln498; R3—Asn487; R4—Gln493, Gly496) forming 11 H-bonds. Furthermore, in complex 3, the Spike-RBD involves three key regions (R1—Gly449, Tyr453; R2—Gln498, Asn501, Thr500; R4—Ser494) to interact with CD152 forming 10 H-bonds (Table 2 and Table S2J, Figure 2D and Figures S26 and S27). Therefore, CD152 may interact with the Spike-RBD and facilitate SARS-CoV-2 attachment and infection.

Therefore, considering the total number of H-bonds and maximum interacting regions and residues of the Spike-RBD, we predicted that CD15, CD27, CD45, and CD152 are top candidates that may act as receptors for the SARS-CoV-2 S protein and can facilitate virus attachment and its subsequent entry into cHL cells (Figure 2A–D).

### 3.3. The SARS-CoV-2 M and ORF3a Proteins Interact with the Human Gamma-Tubulin Complex Components and May Inhibit Tubulin Nucleation

During microtubule (MT) polymerization, the gamma-tubulin ring complex is initially formed as a gamma-tubulin small complex. In this process, each of the GCP2 and GCP3 molecules recruits one TUBG1 molecule at its top and then forms a heterodimer binding through their lateral positions to start the MT nucleation process. Alpha- and beta-tubulin form heterodimers, and through alpha-tubulin, these heterodimers bind to gamma-tubulin. The other GCPs (GCP4, 5, and 6) also follow the same process, and finally, the microtubule polymerization starts [41,42].

As part of our first hypothesis, next, we focused on identifying whether any SARS-CoV-2 protein interacts with the tubulin complex-forming components and blocks the microtubule formation to arrest the cell cycle. According to Gordon et al. [43], the SARS-CoV-2 M protein interacts with the human gamma-tubulin complex component 2 (TUBGCP2/ GCP2) and gamma-tubulin complex component 3 (TUBGCP3/GCP3). Similarly, Chen et al., reported that SARS-CoV-2 ORF3a potentially interacts with GCP2, GCP3, and even gamma-tubulin (TUBG1) [44]. However, in none of the cases, the authors have provided any further details about the binding sites and the effect of these interactions.

Therefore, if the M or ORF3 protein inhibits MT nucleation to restrict the cell cycle, it should bind to any of these three possible areas: (i) the sites where the GCP2 and GCP3 bind laterally to form heterodimers (ii) the gamma-tubulin binding sites of GCP2 or CGP3, and (iii) the TUBG1 sites that are used to interact with GCP2 or GCP3.

**Figure 3:**
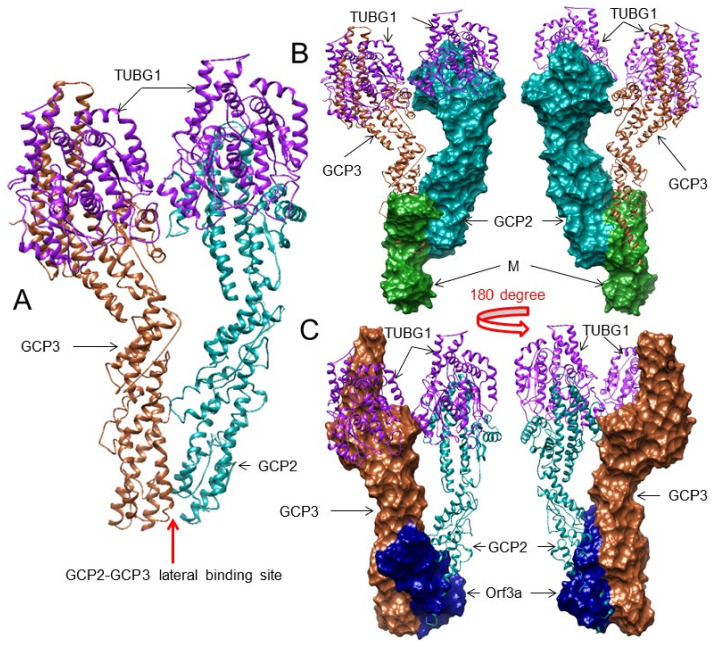
The 3D structures (surface and superimposed views) of the M protein and ORF3a interactions with the GCP2–GCP3 complex. For interacting residues, see the corresponding supplement 2D Figure(s) and Table 3. (**A**) The lateral binding positions of GCP2 (dark cyan) and GCP3 (sienna) in the native human gamma-tubulin ring complex crystal structure (PDB: 6V6B), corresponding to Figure S28. (**B**) Binding of the M protein (forest green) to GCP2 (dark cyan) at its GCP3 binding lateral position (front and 180-degree rotation views, corresponding to complex 7 and Figure S33. (**C**) Binding of the ORF3a protein (navy blue) to GCP3 (sienna) at its GCP2 binding lateral position (front and 180-degree rotation views, corresponding to complex 7 and Figure S36).

To understand this interaction, first we identified the binding residues between GCP2 and GCP3. For this purpose, we used two crystal structures: the human GCP2–GCP3 complex (PDB: 6V6B) and the human gamma-tubulin ring complex (PDB: 6V6S). Next, we standardized the residue positions of PDB: 6V6S corresponding to PDB: 6V6B, and we noted the interacting residues in the GCP2–GCP3 complex according to PDB: 6V6B using Ligplot+ v.2.2. We identified the interacting residues between GCP2-GCP3 at lateral positions as GCP2: Ser283, Gly396, Glu242, Gln350; GCP3: Arg252, Ser323, His343, Gln529, which form three H-bonds and one salt bridge (Figure 3A and Figure S28). We found that the chains C/G (GCP2) and B/F (GCP3) of PDB: 6V6S show similar interactions as the interactions found in PDB: 6V6B.

Since there is no crystal structure for GCP2–TUBG1 and GCP3–TUBG1, we identified the interactions between TUBG1–GCP2 and TUBG1–GCP3 the gamma-tubulin ring complex (PDB: 6V6S) using Ligplot+ v.2.2 and considered that at least two complexes in this ring complex should show similar interactions. Furthermore, the corresponding residues of GCP3 and TUBG1 were identified in PDB: 6V6B and PDB: 6V5V through structural overlapping using UCSF Chimera. A similar approach was applied to identify GCP2 and TUBG1 interacting residues. We observed that GCP2 (chains C/G) (PDB: 6V6B) interacts with TUBG1 (PDB: 6V5V) using 10 H-bonds and two salt bridges (TUBG1: Arg3, Arg47, Tyr248, Ile254, Ser259, Pro262, Asp329, Pro353, Ala354, Gln357; GCP2: Asp554, Asp561, Cys684, Arg711, Asn716, Gln719, Asn720, Glu731, His735, Asn890) (Figures S29 and S30) and GCP3 (chains B/F) (PDB: 6V6S) interacts with TUBG1 (PDB: 6V5V) using 14 H-bonds and three salt bridges (TUBG1: Arg3, Thr45, Arg47, Pro162, Lys163, Asn198, Tyr248, Asp252, Pro265, His334, Arg341, Trp351, Ser355, Gln357; GCP3: Asp572, Arg575, Lys671, Asp579, Asn609, Arg681, Lys682, Cys686, Lys689, Ser709, Gln717, Gln719, Glu725, Glu884, His885) (Figures S31 and S32).

### 3.4. The M and ORF3a Proteins May Interact at GCP2–GCP3 Lateral Binding Sites

To explore if the M and ORF3a proteins hinder the lateral binding of GCP2 and GCP3, we selected the GCP2 (GCP2: Ser283, Gly396, Glu242, Gln350) and GCP3 (GCP3: Arg252, Ser323, His343, Gln529) interacting residues and docked them with the M and ORF3a proteins using the HDOCK server. Among the four residues of GCP2 that form three H-bonds with GCP3, the M protein may interact with two residues (Ser283 and Gln350) along with two nearby residues, forming six H-bonds (complex-7) (Table 3 and Table S3A, Figure 3B and Figure S33). In complex 3, GCP2 interacts with the M protein, forming nine H-bonds (Thr235, Ser243, Arg290, Ser393) (Table 3 and Table S3A, Figure S34). However, the residues are not the same as the ones that interact with GCP3. When we performed the docking between the SARS-CoV-2 M protein with GCP3 targeting its GCP2 binding residues, only one residue (Arg252) of GCP3 interacted in three different complexes (complex-4, -6, -7). However, other interacting residues of GCP3 are not also close to the mapped interacting residues involved in GCP2-GCP3 interaction (Table S3B).

We performed a similar analysis with the ORF3a protein as we did for the M protein. Although the ORF3a protein interacts with GCP2 with a maximum of seven H-bonds (complex 4), it does not involve any residue of GCP2 that interacts with GCP3 (Table 3 and Table S4A, Figure S35). On the other hand, the ORF3a protein potentially interacts with GCP3 at its GCP2 binding site. In complex 7 it makes five H-bonds, and one salt bridge involves two residues (out of four) of GCP3 (Ser323, Arg252, His343-salt bridge) that are involved in lateral binding with GCP2 and the other two residues (Gln322, Arg333) (Table 3 and Table S4B, Figure 3C and Figure S36). In complex 3 it makes seven H-bonds and involves seven residues of GCP3; however, only one residue (Arg252) interacts with GCP2 (Table 3 and Table S4B, Figure S37).

Taken together, the M protein may not bind to GCP3 at its GCP2 interacting site. However, the M protein possibly interacts with GCP2 at its GCP3 binding site using two residues (out of four) (Table 3, Figure 3A,B and Figures S28 and S36). On the other hand, the ORF3a may interact with GCP3, blocking at least three (out of four) of its GCP2 binding residues (Table 3 and Table S4B, Figure 3A,C and Figure S36). Therefore, the potential interactions of M–GCP2 or ORF3a–GCP3 may affect the GCP2–GCP3 lateral binding and hinder microtubule nucleation.

### 3.5. Both the M and ORF3a Proteins May Interact with GCP3 at Its TUBG1 Binding Sites

To understand if the M and ORF3a proteins interact with TUBG1, we docked both proteins at four different positions: (i) TUBG1 binding sites/residues of GCP2, (ii) TUBG1 binding sites/residues of GCP3, (iii) GCP2 binding sites/residues of TUBG1, and (iv) GCP3 binding sites/residues of TUBG1.

**Figure 4:**
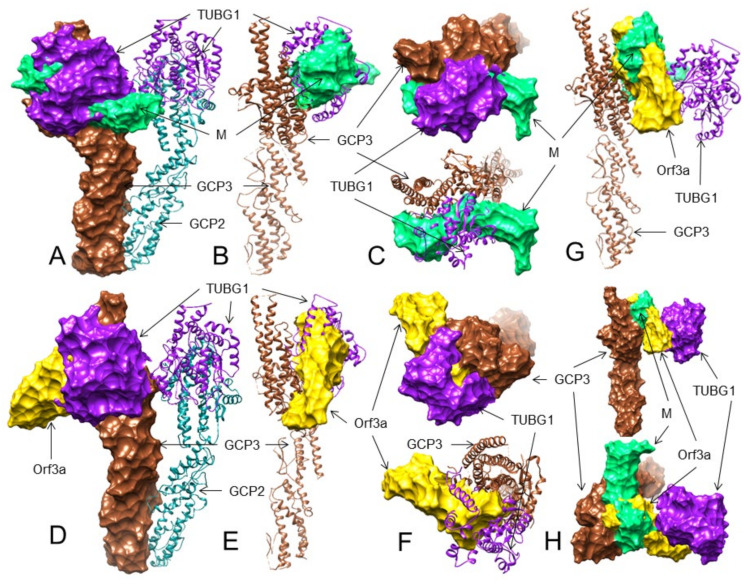
The 3D structures (surface, ribbon, and superimposed views) of the M protein and ORF3a protein interactions with GCP3 at its TUBG1 binding site. For interacting residues, see the corresponding supplement 2D Figure (s) and Table 3. (**A**) Superimposed (front view) structure showing the SARS-CoV-2 M protein (spring green) binding to GCP3 (sienna) at its TUBG1 (purple) binding position, blocking GCP3-TUBG1 interaction (corresponding to complex 1, Figure S40); (**B**) its lateral view; and (**C**) its top views. (**D**) Front view structure showing the SARS-CoV-2 ORF3a protein (gold) binding to GCP3 (sienna) at its TUBG1 (purple) binding position, blocking GCP3-TUBG1 interaction (corresponding to complex 1, Figure S44); (**E**) its lateral view; and (**F**) its top views. (**G**) Superimposed (front view) complex of both the M and ORF3a protein binding to GCP3 at its TUBG1 binding site; and (**H**) its front and top views.

Out of 10 GCP2 residues that interact with TUBG1, the M protein can maximally bind to three such GCP2 residues (Asp554, Arg711, Asp561) and two additional residues (Tyr723, Ser885) in complex 5, forming six H-bonds (Table 3 and Table S3C, Figure S38). In complex 1, the interactions show five H-bonds. However, only 3 GCP2 residues (Glu731, Cys684, Ser885) interact with TUBG1 (Table 3 and Table S3C, Figure S39). The M protein interacts with GCP3 using maximally 10 H-bonds involving three key residues (Lys671, Lys689, Ser709) and six other residues of GCP3 in complex 1 (Table 3 and Table S3D, Figure 4A–C and Figure S40). In complex 5, the M protein binds to three TUBG1 interacting residues of GCP3 (Asp572, Arg681, Ser709) and four other residues (Gly571, Phe612, His716, Asn883), which are close to the TUBG1 interacting residues of GCP3 forming eight H-bonds (Table 3 and Table S3D, Figure S41).

The ORF3a protein binds to the TUBG1 interacting GCP2 residues in two complexes (complex 3 and 9), forming seven H-bonds. In complex 9, seven residues are involved where four residues (Gln719, Cys684, Asn716, Asp561) interact with TUBG1 (Table 3 and Table S4C, Figure S42). However, in complex 3, only four residues of GCP2 interact with the ORF3a protein, where three residues (Cys684, Gln719, Asn890) of GCP2 interact with TUBG1 (Table 3 and Table S4C, Figure S43). In GCP3 (TUBG1 binding site) -ORF3a interaction, in complex 1, GCP3 interacts with the ORF3a protein involving seven residues and 12 H-bonds, where three residues of GCP3 (Arg681, Ser709, Gln717) interact with TUBG1 (Table 3 and Table S4D, Figure 4D–F and Figure S44). In complex 3 we found that five H-bonds and only two such GCP3 residues (Asn609, Gln717) interact with the ORF3a protein (Table 3 and Table S4D, Figure S45).

Therefore, our results suggest that the SARS-CoV-2 M protein may interact with the TUBG1 binding site of GCP3 and probably block GCP3 binding to TUBG1. However, considering the number of H-bonds and the number of GCP residues involved, the ORF3a protein may bind to GCP3 at its TUBG1 binding site more strongly than GCP2. The probable 3D interactions are shown in Table 3 and Figure 4A–F. Since both the M and ORF3a proteins are probably interacting with GCP3 at its TUBG1 binding sites, we also performed a superimposition analysis that also shows the M and ORF3a protein interaction with GCP3 may inhibit TUBG1 binding to GCP3 (Table 3, Figure 4G,H). Nevertheless, additional analyses are needed if these interactions of the M and ORF3a proteins inhibit TUBG1 binding to GCP3.

### 3.6. The M but Not the ORF3a Protein May Strongly Interact with TUBG1 at Its GCP3 Binding Sites

**Figure 5:**
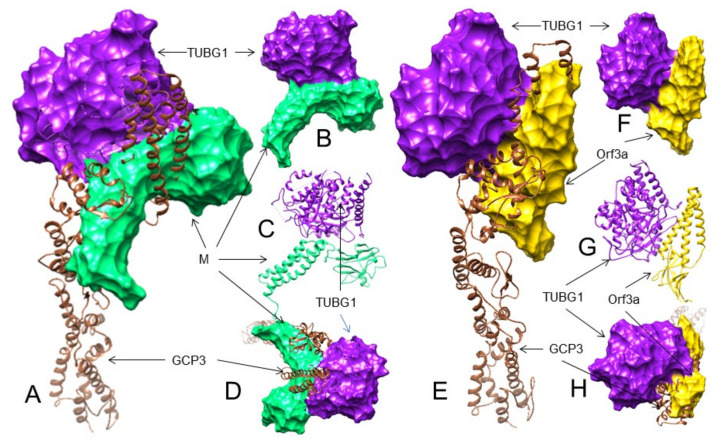
The 3D structure (surface, ribbon, and superimposed views) of TUBG1-M and TUBG1-ORF3a interactions at the GCP3 binding site of TUBG1. For interacting residues, see the corresponding supplement 2D Figure(s) and Table 3. (**A**) Superimposed view of the M protein (spring green) binding to TUBG1 (purple) at its GCP3 binding position, blocking GCP3–TUBG1 interaction (corresponding to complex 1, Figure S49); (**B**) front surface view; (**C**) front ribbon view; and (**D**) superimposed top view. (**E**) Superimposed view of the ORF3a protein (gold) binding to TUBG1 (purple) at its GCP3 binding position, blocking GCP3–TUBG1 interaction (corresponding to complex 1, Figure S54); (**F**) front surface view; (**G**) front ribbon view; and (**H**) superimposed top view.

In docking studies of the M protein with TUBG1 binding sites that interact with GCP2, no interaction involving the GCP2 binding residue of TUBG1 was detected except in complex 1, where only one matching residue (Tyr248) was observed. However, in this model, we found nine H-bonds (Table 3 and Table S3E, Figure S46). Similarly, in complexes 7 and 8, the M protein interacted with TUBG1 using 9 and 10 H-bonds, respectively (Table 3 and Table S3E, Figures S47 and S48). For TUBG1 binding sites for GCP3, we got better results. The M protein can bind with nine H-bonds (complex-1) and involves five residues (Tyr248, Asp252, His334, Arg341, Trp351) of TUBG1 (out of 14) that interact with GCP3 (Table 3 and Table S3F, Figure 5A–D and Figure S49). In complexes 8 and 9, the M protein interacts with TUBG1 making 10 and 7 H-bonds, respectively. In complex 8 it uses two common residues (Pro162, Arg265), and in complex 9 four common residues (His334, Arg341, Ser355, Gln357) (Table 3 and Table S3F, Figures S50 and S51).

In the ORF3a protein, although it makes seven H-bonds in complex 3, it does not involve any TUBG1 residue that interacts with GCP2. However, in complex 1, only four H-bonds are formed by four residues, where only two residues (Tyr248, Gln357) of TUBG1 are involved in binding with GCP2 (Table 3 and Table S4E, Figures S52 and S53). In the case of GCP3, we also found the same trend. Out of 16 TUBG1 residues, that interact with GCP3, only four of them (Tyr248, His334, Arg341, Gln357) probably bind to the ORF3a protein, forming four H-bonds in complex 1 (Table 3 and Table S4F, Figure 5E–H and Figure S54). In this complex, His334 and Arg341 form H-bonds with the ORF3a protein. However, in TUBG1-GCP3 interaction, these two residues of TUBG1 form salt bridges. In complex 3, a total of six residues of TUBG1 (Arg212, Asp216, Val305, Arg343, Arg390, Gln394) bind with the ORF3a protein making seven H-bonds. However, none of these TUBG1 residues interact with GCP3 (Table 3 and Table S4F, Figure S55).

Taken together, our analysis suggests that the M protein may bind to TUBG1, blocking its binding to GCP3 more strongly than GCP2. The probable 3D interactions between TUBG1-M are shown in Table 3 and Table S3F, Figure 5A–D and Figure S49. Considering the number and specific residues of TUBG1 involved in GCP2 and GCP3 interactions, it is unlikely that the same residues interact with the ORF3a protein and interfere with TUBG1 binding to GCP2 and GCP3 (Table 3 and Table S4F, Figure 5E–H and Figure S54).

### 3.7. LMP-1 Signaling Domains of EBV Potentially Interact with SARS-CoV-2 Proteins

The LMP-1 protein of EBV is essential for EBV-associated oncogenesis. Since the cHL patient described by Challenor and Tucker [2] tested positive for EBV, we assess in our second hypothesis that some SARS-CoV-2 proteins might inhibit the oncogenic signaling of LMP-1 by binding to its cytoplasmic signaling motif residues 204-208 aa/“PQQAT” and 379-384 aa/“PVQLSY” that recruit TRAFs and TRADD, respectively, to activate the oncogenic NF-kB signaling-induced B-cell proliferation [26].

To test this second hypothesis, we performed a SARS-CoV-2 proteome-wide docking with our modeled LMP-1 using ZDOCK. In this docking, we selected the LMP-1 residues 202-210 aa (TRAFs binding site) and 375-386 aa (TRADD interacting site) and docked them with SARS-CoV-2 proteins without selecting any residues. We considered few principles in this analysis, (i) since the LMP-1 interacting motifs of TRAFs or TRADD are only 4-5 amino acids of length, a SARS-CoV-2 protein must interact with at least two critical amino acids of any of these motifs, and (ii) since more than two ZDOCK models should show the same interacting amino acids of TRAFs or TRADD binding motifs with hydrogen bonds.

Based on these criteria, we found that out of the 25 SARS-CoV-2 proteins we docked, seven proteins, namely PL^pro^/NSP3 (Gln381, Ser383, Tyr384, Asp386), 3CL^pro^/M^pro^ (Ser383, Tyr384, Tyr385), NSP7 (Gln381, Ser383, Tyr384, Tyr385), NSP10 (Gln381, Ser383, Tyr384, Tyr385), RdRp/NSP12 (Gln381, Ser383, Tyr385, Asp386), Spike (Gln381, Tyr384, Tyr385), and ORF8 (Gln381, Ser383), which may potentially block the TRADD interacting motif of LMP-1 (Table 4, Table S5A–Y).

**Figure 6:**
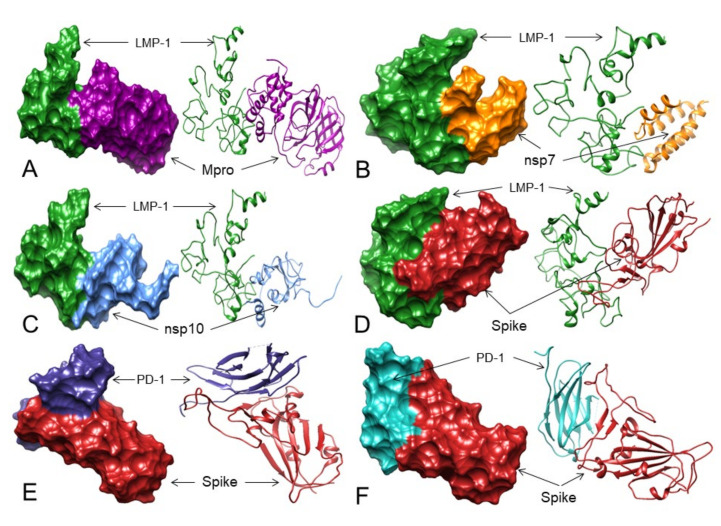
The 3D structures (surface and ribbon views) of SARS-CoV-2 proteins binding to LMP-1 and PD-1—Spike-RBD interactions. For interacting residues, see Table 4 and corresponding supplement 2D Figure(s). (**A**) 3CL^pro^/M^pro^ (dark magenta) binding to LMP-1 (forest green) (corresponding to complex 9, Figure S57, seven H-bonds); (**B**) NSP7 (orange) binding to LMP-1 (forest green) (corresponding to complex 7, Figure S60, eight H-bonds); (**C**) NSP10 (cornflower blue) binding to LMP-1 (forest green) (corresponding to complex 2, Figure S62, nine H-bonds); and (**D**) Spike protein (fire brick) binding to LMP-1 (forest green) (corresponding to complex 6, Figure S64, 5 H-bonds). (**E**) The Spike-RBD (fire brick) binding to PD-1 (dark slate blue) at its nivolumab binding sites (corresponding to complex 2, Figure S67, 8 H-bonds); and (**F**) The Spike-RBD (fire brick) binding to PD-1 (dark slate blue) at its pembrolizumab binding sites (corresponding to complex 8, Figure S69, 10 H-bonds).

For activation of NF-kB, TRADD binding to the PVQLSY motif of LMP-1 is essential, and mutations in the Tyr384 and Tyr385 residues of LMP-1 stop the TRADD interaction with LMP-1 [26]. Considering these facts and our results, it is therefore suggested that 3CL^pro^/M^pro^ (Table 4, Figure 6A, Figures S56–S58), NSP7 (Table 4, Figure 6B, Figures S59–S61), NSP10 (Table 4, Figure 6C, Figures S62 and S63), and Spike (Table 4, Figure 6D, Figures S64–S66) proteins of SARS-CoV-2 may bind to the LMP-1 of EBV at its TRADD binding residues, restricting the access of TRADDs to LMP-1, therefore, potentially inhibiting the NF-kB oncogenic signaling for B-cell proliferation in this patient.

### 3.8. Does the Spike Protein Interact with PD-1 to Block Access to PD-L1 and PD-L2?

Finally, we checked our third possible mechanism, i.e., whether the SARS-CoV-2 Spike protein interacts with PD-1 and acts similarly to the T-cell checkpoint inhibiting monoclonal antibodies (mAbs) like nivolumab and pembrolizumab to induce PD-1 mediated apoptosis.

To check the feasibility of this hypothesis, we first mapped the binding residues between PD-1 and these two mAbs from PD-1 in complex with pembrolizumab Fab (PDB: 5GGS) and PD-1 in complex with nivolumab-Fab (PDB: 5WT9) through a literature search [45,46]. It was found that pembrolizumab-Fab interacts with PD-1 residues (Phe63, Asn66, Thr76, Lys78, Glu84, Ser87, Gly90, Lys131, Ala132) and nivolumab-Fab binds to PD-1 residues (Leu25, Ser27, Pro28, Asp29, Arg30, Thr59, Ser60, Leu128, Ala129, Prp130, Lys131, Ala132). After this mapping, we performed Spike-RBD (without selecting any residue) and PD-1 (selecting residues that interact with nivolumab and pembrolizumab) docking using ZDOCK. Although no interaction of the Spike-RBD was detected with PD-1, the complex 2 that forms eight H-bonds showed that the Spike-RBD could interact with PD-1 near the stretch of nivolumab interacting residues of PD-1 (Table 4 and Table S6, Figure 6E and Figure S67). On the other hand, complexes 7 and 8 form 6 and 10 H-bonds, respectively, where the Spike-RBD may interact with the stretch of pembrolizumab interacting residues of PD-1 (Figure 6F and Figures S68 and S69, Table 4 and Table S6).

Although the Spike-RBD does not bind exactly to the pembrolizumab and nivolumab interacting residues of PD-1, it may interact with PD-1 at very close proximity to the PD-1 residues that interact with these two mAbs. Therefore, the Spike-RBD may bind to PD-1 and may act as pembrolizumab and nivolumab-like mAbs to block access to PD-L1 and PD-L2 leading to cHL cell apoptosis and remission.

## 4. Discussion

During the initial days of the COVID-19 pandemic, there were no reports of any direct correlation between SARS-CoV-2 and lymphoma. However, in December 2020, based on a multi-omics approach, we predicted that lymphoma would have an association with COVID-19 [47,48]. In January 2021, Challenor and Tucker first brought to our attention that a 61-year-old man with cHL and who tested positive for EBV showed complete remission of cHL after he was infected with SARS-CoV-2 [2]. The second case was reported by Sollini et al. in February 2021, where a 61-year-old patient suffering from FL showed complete remission upon SARS-CoV-2 infection [3]. The molecular mechanisms behind the SARS-CoV-2 induced remission of these lymphoma cases are yet to be understood.

Challenor and Tucker suggested that SARS-CoV-2 may induce an unknown specific anti-tumor immune response mechanism [2] that may also be responsible for a previously reported case of spontaneous regression of diffuse large B-cell lymphoma (DLBCL) after infection with *Clostridium difficile* [49]. Similarly, Sollini et al. [3] suggested that the SARS-CoV-2-induced remission of their reported FL case was probably due to a “flare phenomenon” as observed in immunotherapy that finally results in an “abscopal effect”.

**Figure 7:**
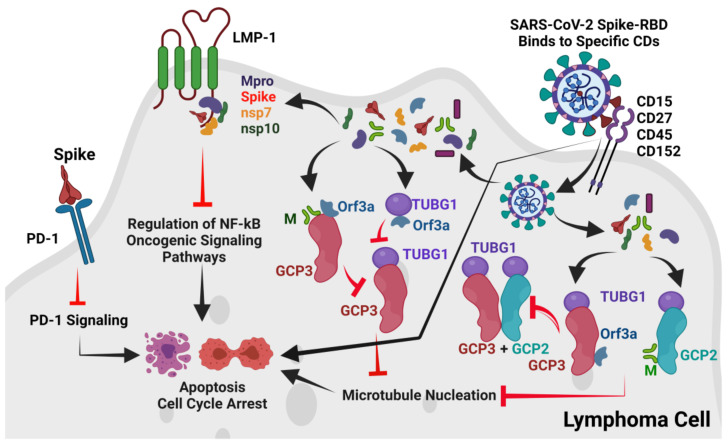
Proposed overall mechanisms of SARS-CoV-2 induced anti-tumor immune response in lymphoma. The SARS-CoV-2 Spike-RBD probably binds to CD15, CD27, CD45, and CD152 and may directly inhibit cell proliferation. Alternately, after binding to these CDs, SARS-CoV-2 may internalize into host cHL or FL cells. After entry into cancer cells, the M and ORF3a proteins block GCP2 –GCP3-TUBG1 interactions and thereby inhibit MT nucleation leading to cell-cycle arrest or cell death. Additionally, the SARS-CoV-2 Spike protein may also interact with PD-1 to block PD-1 signaling, leading to cell-cycle arrest or cell death. The SARS-CoV-2 M^pro^, Spike, NSP7, and NSP10 were also found to potentially interact with the TRADD binding motif of the EBV oncogenic LMP-1 protein. This interaction may also lead to the regulation of the NF-kB oncogenic signaling pathway in cHL or FL. The Figure is developed by BioRender (www.biorender.com, accessed on 24 September 2021).

A recent report in May 2021 from Spain also suggested that SARS-CoV-2 triggers an anti-tumor immune response in lymphoma [50]. In addition, a 22-year-old Hodgkin lymphoma patient suffering from COVID-19 was successfully treated with the PD-1 inhibitor pembrolizumab [51], and the cHL was generally treated with CD30 targeting brentuximab vedotin [18]. Therefore, like the anti-tumor mechanisms of other oncolytic ssRNA viruses [4,8,9,10,11], we also focused on possible anti-tumor immune responses by SARS-CoV-2 in the reported cHL and FL cases, hypothesizing three possible mechanisms. As per our results, the proposed overall mechanisms of SARS-CoV-2-induced remission of cHL and FL cases are presented in Figure 7.

ACE2 is expressed in lymphomas [52] and SARS-CoV-2 infection is associated with increased severity and mortality in lymphomas [53]. However, these SARS-CoV-2-induced remissions of cHL and FL are very isolated events [2,3] compared to global cHL and FL cases and the magnitude of the COVID-19 pandemic. Additionally, the molecular profiles of these patients [2,3] are unknown. Therefore, it is not likely to be a general mechanism to explain this remission phenomenon. Our first hypothesis-based results show that, the initial contact and attachment of SARS-CoV-2 to cHL or FL cells in these two cases may be possible through the interactions between the SARS-CoV-2 Spike-RBD and cell-surface markers for cHL or FL such as CD15, CD27, CD45, and CD152 (Table 2, Figure 2A–D).

Previous reports suggest that some unclassified FL expresses CD15 [54] and CD27 [55]. Expression of CD152 is also reported in cHL [12,56]. CD15 is also expressed in some specific groups of cHL patients [57,58] and its expression can predict the disease outcome [59]. Circulating blood cells in cHL patients show clonal expression of CD27 [60]. CD15 is also required for cell adhesion to platelets to promote cell proliferation and migration beyond the lymphatic system [61]. Therefore, CD15 may be a potential target in cHL. CD27 activates protein kinase C (PKC) and induces cellular proliferation in B-cell lymphomas [62] and anti-CD27 mAb shows antitumor activity [63]. CD45 is also aberrantly expressed in certain cases of lymphomas [64,65], and phosphatase activity of CD45 is required for lymphoid cell proliferation [66]. Inhibition of CD45 phosphatase activity negatively regulates Src family tyrosine kinase (SFK) signaling and thereby induces G2/M cell-cycle arrest and cell apoptosis [66]. Therefore, CD45 could be a possible therapeutic target in lymphoma. Similarly, activation of CD152/ CTLA4 induces cell proliferation and tumor growth in lymphomas through activation of the TYK2-STAT3 pathway [67], and anti-CD152 mAb ipilimumab could be a potential targeted therapeutic for lymphomas [67,68]. PD-1 is upregulated in lymphomas, a hallmark of EBV-associated lymphoproliferative disorders, and this overexpression is associated with disease relapse [69,70]. Dual targeting of PD-1 and CD152 using combinations of anti-PD-1 and anti- CD152 mAbs may be a potential therapy in relapsed lymphoid malignancies [71].

Based on these facts, if we assume that these two patients [2,3] express any of these very specific receptors such as CD15, CD27, CD45, and CD152 that are not commonly expressed in cHL and FL, and that the SARS-CoV-2 Spike-RBD may bind to any of these receptors like a mAb and thereafter works in a similar fashion to brentuximab vedotin (SGN-35 or Adcetris), this suggests a possible molecular mechanism of the remission of the lymphoma patients as described by Challenor and Tucker [2] and Sollini et al. [3]. As the expression of CD15, CD27, CD45, and CD152 is also associated with lymphoma cell proliferation these CD molecules can be therapeutic targets [61,62,63,66,67,68]. If the Spike-RBD interaction inhibits the CD expression, it may also induce cell-cycle arrest and apoptosis.

Our analysis found that the Spike-RBD can potentially interact with CD15, CD27, CD45, and CD152 expressed by lymphoma cells (Table 2, Figure 2A–D). As per our first hypothesis, after attachment to lymphoma cells through CD15, CD27, CD45 or CD152, the SARS-CoV-2 enters into lymphoma cells by an unknown mechanism and upon cellular entry, it will use some of its proteins to initiate cell-cycle arrest or apoptosis by interacting with cell-cycle machinery components. Gordon et al. [43] and Chen et al. [44] reported that the M and ORF3a proteins of SARS-CoV-2 interact with the gamma-tubulin complex components GCP2, GCP3, and TUBG1. The binding of GCP2-TUBG1, GCP3–TUBG1, and GCP2–GCP3 is essential for the microtubule nucleation process [41,42]. Our analysis suggests that the M protein may bind to GCP2 at its GCP3 binding site (Figure 3B), and the ORF3a protein may bind to GCP3 at its GCP2 binding residues (Table 3, Figure 3C); thus, both the M and ORF3a proteins can potentially render the GCP2 –GCP3 lateral binding. Further, the M and ORF3a proteins may also bind to GCP3 at its TUBG1 binding site (Table 3, Figure 4A–H). Additionally, we also predicted that the M and ORF3a proteins might bind to TUBG1, blocking its interaction with GCP3 (Table 3, Figure 5A–H). Therefore, if the M and ORF3a proteins are involved in such interactions, the microtubule nucleation process will stop leading to cell-cycle arrest.

Some coronavirus infections induce apoptosis in host cells that may be required for viral replication and propagation in respective hosts [72]. SARS-CoV and SARS-CoV-2 induce apoptosis through distinct mechanisms [73]. SARS-CoV uses the 7a protein to induce apoptosis, activating the p38 MAPK pathway [74]. SARS-CoV also uses the S, M, and N proteins for induction of apoptosis [75,76]. Recently, the SARS-CoV-2 ORF3a protein was reported to induce apoptosis via the release of cytochrome C [77]. As per our prediction, the ORF3a protein may also be involved in cell-cycle arrest at a very early stage, leading to apoptosis or cell death (Table 3, Figure 4D–F and Figure 5E–H).

PD-1 immune-checkpoint-blocking mAbs such as pembrolizumab and nivolumab are used in cHL [21,22] and FL [20] treatment to induce apoptosis. Although we do not know if the reported cHL and FL cases [2,3] are PD-1 positive, we analyzed whether the SARS-CoV-2 Spike protein may act like pembrolizumab and nivolumab, which bind to PD-1 to block the access of PDL-1 and PDL-2 and thus downregulate PD-1 signaling and induce apoptosis. Our analysis observed that the Spike-RBD potentially interacts with PD-1 at the binding sites of these two mAbs, although only a few overlapping residues of PD-1 that interact with pembrolizumab/nivolumab are found to interact with the Spike-RBD (Table 4, Figure 6E,F). Therefore, we suggest further validating whether the SARS-CoV-2 Spike protein can also induce apoptosis through negative regulation of PD-1 mediated signaling as per the results of our second hypothesis.

Our previous report predicted that the SARS-CoV-2 infection pathway is involved in crosstalk with other viral pathways, including EBV [47]. Co-infection of EBV in COVID-19 patients is not uncommon, and in COVID-19 patients, reactivation of EBV has been reported, which may be associated with disease severity and other symptoms of long COVID-19 [78,79,80,81]. Additionally, patients with lymphoproliferative disorders showing immunodeficiency and post-traNSPlantation patients subjected to immunosuppression, the synergistic action of EBV and SARS-CoV-2 may increase the fatality rate [82]. The cHL patient of Challenor and Tucker was EBV positive [2], and the LMP-1 of EBV is the main oncogenic protein of EBV that activates oncogenic signaling through activation of NF-κB, JAK/STAT, and PI3K/AKT pathways through its cytoplasmic TRAFs and TRADD binding motifs [23,26]. Contrary to the reported potential synergistic association between EBV and SARS-CoV-2 [78,79,80,81,82], in our analysis (as per our third hypothesis), we found that the 3CL^pro^/M^pro^, NSP7, NSP10, and S (Table 4, Figure 6A–D) proteins of SARS-CoV-2 may interact at the TRADD binding sites of LMP-1 thus blocking the access of TRADDs to LMP-1, and this interaction may inhibit the LMP-1-mediated NF-kB oncogenic signaling to induce remission. However, further investigations are required to validate these interactions and their outcomes in lymphoma remission.

## 5. Conclusions

This study has explored the possible molecular mechanisms behind the rare phenomenon of lymphoma remission upon SARS-CoV-2 infection. We have focused on a specific anti-tumor immune response caused by SARS-CoV-2. We are currently planning to conduct in vitro experimental validations of our results and if our proposed mechanism is proven right, SARS-CoV-2 may be engineered for effective therapeutic interventions against certain lymphomas and other proliferative disorders.

## Figures and Tables

**Table 1:** Ramachandran plot information of the modeled proteins.

Proteins	% of Residues in			
	Most Favored Regions	Additional Allowed Regions	Generously Allowed Regions	Disallowed Regions
**Human CD30**	87.0	11.0	1.4	0.6
**Human CD15**	92.0	7.5	0.0	0.5
**EBV LMP-1**	96.0	4.0	0.0	0.0
**SASR-CoV-2 M protein**	92.5	7.0	0.5	0.0

**Table 2:** Details of interacting top four human CD protein residues (bold) with SARS-CoV-2 RBD residues (Roman).

Protein Complexes	Docked Complex Number	Interacting Residues
**CD15- Spike RBD**	3	**Trp-500**-Tyr505, **Arg428**-Thr500, **Glu502**-Gln498, **Glu-502**-Tyr449, **Trp504**-Gly446, **Asp-210**-Tyr453, **Pro207**-Ser494, **Arg206**-Tyr449, **Arg206**-Ser494, **Arg206**-Asn450, **Leu178**-Tyr489, **Leu178**-Asn487
	4	**Gly340**-Tyr449, **Ser344**-Gln498, **Ser344**-Gly496, **Ser344**-Asn501, **Arg449**-Asn487, **Leu333**-Tyr489, **Glu448**-Tyr473, **Ser337**-Ser494, **Ser337**-Leu492, **Arg336**-Gln493
	6	**Arg214**-Gly496, **Asp210**-Tyr449, **Arg212**-Glu484, **Ser498**-Gly446, **Leu178**-Lys417, **Gln174**-Asp405, **Tyr169**-Tyr505, **Cys171**-Gly502
**CD27- Spike RBD**	8	**Ile46**-Gln498, **Ser50**-Gln498, **Thr57**-Gln493, **Leu29**-Tyr453, **His60**-Tyr495, **Cys61**-Tyr449, **Gln15**-Tyr421, **Trp13**-Ala475, **Trp13**-Tyr489
	9	**Arg87**-Tyr495, **Trp90**-Gln498, **Asn88**-Gln498, **Asn88**-Thr500, **Gly70**-Gln493, **Leu72**-Tyr453, **Leu72**-Arg403, **Ser63**-Asn487, **Ser63**-Tyr489, **Gly48**-Lys417
**CD45- Spike RBD**	3	**Lys210**-Glu484, **His191**-Cys488, **Ile235**-Asn487, **Lys157**-Tyr473, **Lys157**-Arg457, **Pro186**-Gln493, **Gln187**-Arg403, **His134**-Asn460, **Asn184**-Gln498
	8	**Ile235**-Asn487, **His191**-Cys488, **Pro186**-Gln493, **Gln187**-Tyr453, **Asn184**-Gln498, **Gln167**-Asn501
**CD152- Spike RBD**	2	**Asp118**-Tyr449, **Asp118**-Gly496, **Pro119**-Gln498, **Gly111**-Asn487, **Ala86**-Gln493, **Met87**-Ser494, **Ala42**-Tyr421, **Gln45**-Asp420, **Gln45**-Tyr421, **Asp43**-Arg457
	3	**Met87**-Gln498, **Asp88**-Asn501, **Asp88**-Thr500, **Arg40**-Gly446, **Tyr104**-Tyr489, **Ile67**-Tyr505, **Glu48**-Tyr453, **Thr47**-Ser494, **Val46**-Ser494

**Table 3:** Interacting human microtubule ring complex protein residues (bold) with SARS-CoV-2 protein residues (Roman).

Protein Complexes	Docked Complex Number	Interacting Residues
**GCP2-CoV-2 M Protein** (Lateral Position)	7	**Arg290**-Cys33, **Arg290**-Thr30, **Trp353**-Thr30, **Trp353**-Phe26, **Gln350**-Lys50, **Ser283**-Asn43
	3	**Ser243**-Ser173, **Ser243**-Arg174, **Thr235**-Arg198, **Arg290**-Leu56, **Ser393**-Asn5, **Ser393**-Thr7
**GCP2- Orf3a** (Lateral Position)	4	**Arg361**-Asp210, **Ser393**-Ile236, **Glu242**-Lys61, **Asp397**-Ser209, **Trp353**-Asp142, **Arg290**-Tyr189
**GCP3-Orf3a** (Lateral Position)	7	**Arg333**-Asp142, **Arg252**-Ser165, **Ser323**-Lys61, **Gln322**-Lys61, **His343**-Asp210
	3	**Tyr256**-Val163, **Arg252**-Val163, **Arg337**-Ile118, **Arg333**-Asn119, **Gln416**-Tyr107, **Arg315**-Lys61, **Gln322**-Ser58
**GCP2-M Protein** (At GCP2 residues that interact with TUBG1)	5	**Asp554**-Lys205, **Tyr723**-Asn117, **Arg711**-Glu137, **Asp561**-Thr208, **Ser885**-Arg101
	1	**Ser885**-Arg105, **Ser885**-Arg101, **Cys684**-Cys86, **Glu731**-Gln36
**GCP3-M Protein** (At GCP3 residues that interact with TUBG1)	1	**Phe612**-Gln36, **Gln570**-Gln36, **Ser709**-Arg131, **Ser709**-His154, **Lys689**-Glu137, **Thr678**-Arg107, **Glu728**-Arg42, **Gly569**-Arg42, **Tyr720**-Asn41, **Lys671**-Tyr39
	5	**Phe612**-Gln36, **Arg681**-Glu137, **Glu728**-Arg42, **Ser709**-His154, **His716**-Arg107, **Asn883**-His155, **Gly571**-Arg105, **Asp572**-Arg105
**GCP2-Orf3a** (At GCP2 residues that interact with TUBG1)	3	**Cys684**-Val163, **Gln719**-Ser166, **Tyr723**-Ser166, **Asn890**-Tyr215
	9	**Gln722**-Lys192, **Gln719**-Trp193, **Cys684**-His182, **Asn716**-Cys153, **Gln712**-Asn152, **Asp561**-His227, **Arg681**-Gly174
**GCP3-Orf3a** (At GCP3 residues that interact with TUBG1	1	**His716**-Asp210, **Tyr720**-Asp210, **Asn883**-Asn144, **Gln717**-Tyr145, **Ser709**-Ser162, **Arg681**-Ser166, **Arg681**-Thr164, **Thr678**-Ser165
	3	**His702**-Leu83, **Glu728**-Lys192, **Asn609**-Thr151, **Gln570**-Asn152, **Gln717**-Trp69
**TUBG1-M Protein** (At TUBG1 residues that interact with GCP2)	1	**Pro350**-Arg131, **Trp351**-Arg131, **Asp442**-His125, **Arg341**-Leu134, **Asn251**-Gln36, **His334**-Arg101, **Tyr248**-Tyr47, **Asp252**-Arg42
	7	**Leu276**-Arg44, **Tyr273**-Arg42, **Lys363**-Phe37, **Glu38**-Arg105, **Ser32**-Arg101, **Lys23**-Arg105, **Ser80**-107, **Thr234**-Tyr39, **Thr234**-Asn41
	8	**Glu426**-Arg131, **Pro162**-Arg42, **Asp200**-Arg42, **Gln197**-Trp110, **Pro-264**-Tyr39, **Thr196**-Asn41, **Arg265**-Asn41, **Asp433**-Arg107, **Asp419**-Thr127, **Asp422**-His125
**TUBG1-M Protein** (At TUBG1 residues that interact with GCP3)	1	**Pro350**-Arg131, **Trp351**- Arg131, **Asp442**-His125, **Arg341**-Leu134, **Asn251**-Gln36, **His334**-Arg101, **Tyr248**-Tyr47, **Asp252**-Arg42
	8	**Glu426**-Arg131, **Pro162**-Arg42, **Asp200**-Arg42, **Gln197**-Trp110, **Pro-264**-Tyr39, **Thr196**-Asn41, **Arg265**-Asn41, **Asp433**-Arg107, **Asp419**-Thr127, **Asp422**-His125
	9	**Gln357**-Thr127, **Thr331**-Arg131, **His334**-Leu156, **Ser355**-His125, **Arg341**-Asp160, **Glu327**-Asn41
**TUBG1-orf3a** (At TUBG1 residues that interact with GCP2)	3	**Val130**-Lys136, **Arg212**-Tyr154, **Arg390**-Arg134, **Arg390**-Asp134, **Asp216**-Arg68, **Arg343**-Leu83, **Gln394**-Asn152
	1	**Arg341**-Phe207, **His334**-Tyr211, **Gln357**-Ile124, **Tyr248**-Cys130
**TUBG1-orf3a** (At TUBG1 residues that interact with GCP3)	1	**Arg341**-Phe207, **His334**-Tyr211, **Gln357**-Ile124, **Tyr248**-130
	3	**Val305**-Lys136, **Arg212**-Tyr154, **Arg390**-Asp155, **Arg390**-Arg134, **Asp216**-Arg68, **Arg343**-Leu83, **Gln394**-Asn152

**Table 4:** Interacting Epstein–Barr virus LMP-1 and human PD residues with SARS-CoV-2 protein residues. The non-SARS-CoV-2 protein residues are shown in bold.

Protein Complexes	Docked Complex Number	Interacting Residues
**LMP-1** and **3CL^pro^/M^pro^**	1	**His352**-Asn274, **Tyr384**-Asn274, **Tyr385**-Gly275, **Leu382**-Asn277, **Ser383**-Asn277, **Ser367**-Ala285
	9	**Ser383**-Asn277, **Tyr385**-Arg279, **Tyr384**-Glu270, **Ser313**-Glu270, **Ser313**-Lys269, **Thr324**-Gln273, **Glu328**-Lys236
	10	**Gln381**-Arg298, **Gln381**-Gly302, **Gln381**-Ser301, **Gly345**-Thr304, **Asp341**-Gln306, **Asp341**-Phe305, **Ser347**-Ser1, **Ser383**-Ser1, **Val228**-Arg4, **Ala231**-Phe3
**LMP-1** and **NSP7**	6	**Ser383**-Leu60, **Tyr385**-Leu59, **Tyr385**-Met62, **Ser367**-Met62, **Ser367**-Leu20, **Gly368**-Leu20, **Gly371**-Arg21, **Ser369**-Ser24
	7	**Tyr384**-Gly64, **Ser313**-Lys70, **Tyr385**-Leu59, **Tyr385**-Met62, **Gln381**-Ser61, **Ser367**-Ser61, **Ser367**-Val58, **Gly371**-Ser26
	8	**Ser313**-Lys70, **Ser367**-Leu20, **Tyr385**-Met62, **Tyr385**-Leu59, **Tyr385**-Val66, **Ser383**-Leu60, **Gln381**-Ser61
**LMP-1** and **NSP10**	2	**Ser313**-Asp65, **Ser383**-His63, **Gln381**-His63, **Tyr384**-His63, **Tyr385**-His63, **Ser367**-Arg61, **Gly365**-Cys62, **Glu325**-Asp5
	8	**Tyr385**-Ser112, **Tyr385**-Arg117, **Ser383**-Arg117, **Tyr384**-Cys113, **Gln381**-Glu118, **Ser347**-Leu121, **His346**-Gln122, **Thr324**-Val102
**LMP-1** and **Spike RBD**	6	**His352**-Tyr489, **Gln381**-Gln493, **Tyr384**-Tyr489, **Tyr384**-Asn487, **Tyr385**-Lys417
	7	**Tyr384**-Asn487, **His352**-Asn487, **His352**-Tyr489, **Asp386**-Tyr421, **Ser367**-Leu492, **Ser367**-Phe490, **Ser367**-Gln493
	8	**Pro376**-Asn501, **Pro376**-Gln498, **Gly344**-Tyr453, **Asp341**-Lys417, **Tyr385**-Phe486, **Ser229**-Asn487, **Gln381**-Glu484
**PD-1** and **Spike RBD**	2	**Thr53**-Tyr505, **His107**-Tyr505, **Arg104**-Tyr505, **Ser55**-Tyr453, **Thr36**-Tyr453, **Glu136**-Tyr489, **Lys135**-Cys488, **Trp32**-Glu484, **Asn58**-Tyr449
	7	**Ser127**-Tyr489, **Ala129**-Tyr489, **Leu100**-Tyr449, **Pro101**-Gln498, **Phe63**-Gln493, **Tyr68**-Glu484
	8	**Asp77**-Gln493, **Lys78**-Phe490, **Arg94**-Tyr505, **Ile126**-Phe486, **Gln75**-Tyr449, **Ser71**-Tyr449, **Ser71**-Gly446, **Ser73**-Gly446

## Data Availability

All data are available in supplement materials.

## Supplementary Materials

The following are available online at www.mdpi.com/article/10.3390/v13101927/s1. Figure S1: The Ramachandran plot of the refined CD30 structure. Figure S2: The Ramachandran plot of the refined CD15 structure. Figure S3: The Ramachandran plot of the refined SARS-CoV-2 M protein structure. Figure S4: Refined 3D model EBV_LMP1. Figure S5: The Ramachandran plot of the refined 3D model EBV LMP1 structure. Figure S6: The Ramachandran plot of the RaptorX 3D model EBV LMP1 structure. Figure S7: 2D representation of Spike-RBD interacting with hACE2 (PDB: 6LZG). Figure S8: The four combined regions of Spike-RBD. Figure S9: 2D representation of CD15 (Chain A) docking residues with Spike RBD (Chain B) Complex 3, provided by the LIGPLOT v.2.2 software. Figure S10: 2D representation of CD15 (Chain A) docking residues with Spike RBD (Chain B), Complex 4 provided by the LIGPLOT v.2.2 software. Figure S11: 2D representation of CD15 (Chain A) docking residues with Spike RBD (Chain B), Complex 6 provided by the LIGPLOT v.2.2 software. Figure S12: 2D representation of CD27/ TNFRSF7 (Chain A) docking residues with Spike RBD (Chain B), Complex 8, provided by the LIGPLOT v.2.2 software. Figure S13: 2D representation of CD27/ TNFRSF7 (Chain A) docking residues with Spike RBD (Chain B), Complex 9, provided by the LIGPLOT v.2.2 software. Figure S14: 2D representation of CD30 (Chain A) docking residues with Spike RBD (Chain B), Complex 5, provided by the LIGPLOT v.2.2 software. Figure S15: 2D representation of CD30 (Chain A) docking residues with Spike RBD (Chain B), Complex 3, provided by the LIGPLOT v.2.2 software. Figure S16: 2D representation of CD40 (Chain A) docking residues with Spike RBD (Chain B), Complex 8, provided by the LIGPLOT v.2.2 software. Figure S17: 2D representation of CD40 (Chain A) docking residues with Spike RBD (Chain B), Complex 9, provided by the LIGPLOT v.2.2 software. Figure S18: 2D representation of CD40 (Chain A) docking residues with Spike RBD (Chain B), Complex 3, provided by the LIGPLOT v.2.2 software. Figure S19: 2D representation of CD45 (Chain A) docking residues with Spike RBD (Chain B), Complex 8, provided by the LIGPLOT v.2.2 software. Figure S20: 2D representation of CD80 (ChainA) docking residues with Spike RBD (Chain B) Complex 3, provided by the LIGPLOT v.2.2 software. Figure S21: 2D representation of CD80 (Chain A) docking residues with Spike RBD (Chain B) Complex 9, provided by the LIGPLOT v.2.2 software. Figure S22: 2D representation of CD86 (Chain A) docking residues with Spike RBD Chain B)Complex 2, provided by the LIGPLOT v.2.2 software. Figure S23: 2D representation of CD86 (Chain A) docking residues with Spike RBD (Chain B) Complex 9, provided by the LIGPLOT v.2.2 software. Figure S24: 2D representation of CD95 (Chain F) docking residues with Spike RBD (Chain B) Complex 2, provided by the LIGPLOT v.2.2 software. Figure S25: 2D representation of CD95 (Chain F) docking residues with Spike RBD (Chain B) Complex 10, provided by the LIGPLOT v.2.2 software. Figure S26: 2D representation of CTLA4/CD152 (Chain A) docking residues with Spike RBD (Chain B) Complex 2, provided by the LIGPLOT v.2.2 software. Figure S27: 2D representation of CTLA4/CD152 (Chain A) docking residues with Spike RBD (Chain B) Complex 3, provided by the LIGPLOT v.2.2 software. Figure S28: 2D representation of interacting residues between GCP2-GCP3 (PDB: 6V6B, Chain C: GCP2 & Chain B: GCP3 ), provided by the LIGPLOT v.2.2 software. Figure S29: 2D representation of interacting residues between GCP2-TUBG1 (PDB: 6V6S, Chain C: GCP2 & Chain c: TUBG1). provided by the LIGPLOT v.2.2 software. Figure S30: Representing the interacting residues between GCP2-TUBG1 (PDB: 6V6S, Chain C: GCP2 & Chain c: TUBG1) and its corresponding residues in PDB ID 6V6B (Chain C) and PDB ID 6V5V (Chain g). Figure S31: 2D representation of interacting residues between GCP3-TUBG1 (PDB: 6V6S, Chain B: GCP2 & Chain b: TUBG1). provided by the LIGPLOT v.2.2 software. Figure S32: Representing the interacting residues between GCP3-TUBG1 (PDB: 6V6S, Chain B: GCP2 & Chain b: TUBG1) and its corresponding residues in PDB ID 6V6B (Chain C) and PDB ID 6V5V (Chain g). Figure S33: 2D representation of interacting residues between GCP2 (Chain C) and CoV-2 M Protein (Chain A), Complex 7, provided by the LIGPLOT v.2.2 software. Figure S34: 2D representation of interacting residues between GCP2 (Chain C) and CoV-2 M Protein (Chain A), Complex 3, provided by the LIGPLOT v.2.2 software. Figure S35: 2D representation of interacting residues between GCP2 (Chain C) and Orf3a (Chain A), Complex 4, provided by the LIGPLOT v.2.2 software. Figure S36: 2D representation of interacting residues between GCP3 (Chain B) and Orf3a (Chain A), Complex 7, provided by the LIGPLOT v.2.2 software. Figure S37: 2D representation of interacting residues between GCP3 (Chain B) and Orf3a (Chain A), Complex 3, provided by the LIGPLOT v.2.2 software. Figure S38: 2D representation of interacting residues between GCP2 (At GCP2 residues that interact with TUBG1) (Chain C) and M Protein (Chain A), Complex 5, provided by the LIGPLOT v.2.2 software. Figure S39: 2D representation of interacting residues between GCP2 (At GCP2 residues that interact with TUBG1) (Chain C) and M Protein (Chain A), Complex 1, provided by the LIGPLOT v.2.2 software. Figure S40: 2D representation of interacting residues between GCP3 (AtGCP3 residues that interact with TUBG1) (Chain B) and M Protein (Chain A), Complex 1, provided by the LIGPLOT v.2.2 software. Figure S41: 2D representation of interacting residues between GCP3 (AtGCP3 residues that interact with TUBG1) (Chain B) and M Protein (Chain A), Complex 5, provided by the LIGPLOT v.2.2 software. Figure S42: 2D representation of interacting residues between GCP2 (At GCP2 residues that interact with TUBG1) (Chain C) and Orf3a (Chain A), Complex 3, provided by the LIGPLOT v.2.2 software. Figure S43: 2D representation of interacting residues between GCP2 (At GCP2 residues that interact with TUBG1) (Chain C) and Orf3a (Chain A), Complex 9, provided by the LIGPLOT v.2.2 software. Figure S44: 2D representation of interacting residues between GCP3 (At GCP3 residues that interact with TUBG1) (Chain B) and Orf3a (Chain A), Complex 1, provided by the LIGPLOT v.2.2 software. Figure S45: 2D representation of interacting residues between GCP3 (At GCP3 residues that interact with TUBG1) (Chain B) and Orf3a (Chain A), Complex 3. provided by the LIGPLOT v.2.2 software. Figure S46: 2D representation of interacting residues between TUBG1 ( At TUBG1 residues that interact with GCP2 ) (Chain g) and M Protein (Chain A), Complex 1, provided by the LIGPLOT v.2.2 software. Figure S47: 2D representation of interacting residues between TUBG1 (At TUBG1 residues that interact with GCP2) (Chain g) and M Protein (Chain A), Complex 7, provided by the LIGPLOT v.2.2 software. Figure S48: 2D representation of interacting residues between TUBG1 (At TUBG1 residues that interact with GCP2) (Chain g) and M Protein (Chain A), Complex 8, provided by the LIGPLOT v.2.2 software. Figure S49: 2D representation of interacting residues between TUBG1 (At TUBG1 residues that interact with GCP3 ) (Chain g) and M Protein (Chain A), Complex 1, provided by the LIGPLOT v.2.2 software. Figure S50: 2D representation of interacting residues between TUBG1 (At TUBG1 residues that interact with GCP3) (Chain g) and M Protein (Chain A), Complex 8, provided by the LIGPLOT v.2.2 software. Figure S51: 2D representation of interacting residues between TUBG1 (At TUBG1 residues that interact with GCP3) (Chain g) and M Protein (Chain A), Complex 9,provided by the LIGPLOT v.2.2 software. Figure S52: 2D representation of interacting residues between TUBG1 (At TUBG1 residues that interact with GCP2) (Chain g) and orf3a (Chain A), Complex 3 provided by the LIGPLOT v.2.2 software. Figure S53: 2D representation of interacting residues between TUBG1 (At TUBG1 residues that interact with GCP2) (Chain g) and orf3a (Chain A), Complex 1, provided by the LIGPLOT v.2.2 software. Figure S54: 2D representation of interacting residues between TUBG1 (At TUBG1 residues that interact with GCP3 ) (Chain g) and orf3a (Chain A), Complex 1,provided by the LIGPLOT v.2.2 software. Figure S55: 2D representation of interacting residues between TUBG1 (At TUBG1 residues that interact with GCP3 ) (Chain g) and orf3a (Chain A), Complex 3,provided by the LIGPLOT v.2.2 software. Figure S56: 2D representation of interacting residues between LMP-1 (Chain Y) and 3CLpro/Mpro (Chain A) complex 1, provided by the LIGPLOT v.2.2 software. Figure S57: 2D representation of interacting residues between LMP-1 (Chain Y) and 3CLpro/Mpro (Chain A) complex 9, provided by the LIGPLOT v.2.2 software. Figure S58: 2D representation of interacting residues between LMP-1 (Chain Y) and 3CLpro/Mpro (Chain A) complex 10, provided by the LIGPLOT v.2.2 software. Figure S59: 2D representation of interacting residues between LMP-1 (Chain Y) and NSP7 (Chain A) complex 6, provided by the LIGPLOT v.2.2 software. Figure S60: 2D representation of interacting residues between LMP-1 (Chain Y) and NSP7 (Chain A) complex 7, provided by the LIGPLOT v.2.2 software. Figure S61: 2D representation of interacting residues between LMP-1 (Chain Y) and NSP7 (Chain A) complex 8, provided by the LIGPLOT v.2.2 software. Figure S62: 2D representation of interacting residues between LMP-1 (Chain Y) and NSP10 (Chain B) complex 2, provided by the LIGPLOT v.2.2 software. Figure S63: 2D representation of interacting residues between LMP-1 (Chain Y) and NSP10 (Chain B) complex 8, provided by the LIGPLOT v.2.2 software. Figure S64: 2D representation of interacting residues between LMP-1 (Chain Y) and CoV-2 Spike (Chain B) complex 6, provided by the LIGPLOT v.2.2 software. Figure S65: 2D representation of interacting residues between LMP-1 (Chain Y) and CoV-2 Spike (Chain B) complex 7, provided by the LIGPLOT v.2.2 software. Figure S66: 2D representation of interacting residues between LMP-1 (Chain Y) and CoV-2 Spike (Chain B) complex 8, provided by the LIGPLOT v.2.2 software. Figure S67: 2D representation of interacting residues between PD-1 (Chain G) and CoV-2 Spike (Chain B) complex 2, provided by the LIGPLOT v.2.2 software. Figure S68: 2D representation of interacting residues between PD-1 (Chain G) and CoV-2 Spike (Chain B) complex 7, providedby the LIGPLOT v.2.2 software. Figure S69: 2D representation of interacting residues between PD-1 (Chain G) and CoV-2 Spike (Chain B) complex 8, provided by the LIGPLOT v.2.2 software. Table S1: Protein structures used in this analysis. Table S2: Docking analysis of human CDs and SARS-CoV-2 Spike-RBD. Table S3: Docking analysis of M with GCP2 at its GCP3 binding site, M with GCP3 at its GCP2 binding site, M with TUBG1 at its GCP2 and GCP3 binding sites. Table S4: Docking analysis of Orf3a with GCP2 at its GCP3 binding site, Orf3a with GCP3 at its GCP2 binding site, Orf3a with TUBG1 at its GCP2 and GCP3 binding sites. Table S5: Analysis of SARS-CoV-2 proteome wide docking with the TRAFs and TRADD interacting motifs of LMP-1. Table S6: Docking analysis of Spike-RBD with the pembrolizumab and nivolumab interacting sites of human PD-1. Supplementary File S1: Intrinsic disorder prediction of human CD15/FUT4, human CD30/TNFRSF8, SARS-CoV-2 membrane (M) protein, and Epstein–Barr virus LMP-1 proteins.

## Abbreviations

hACE2	Human Angiotensin-converting enzyme 2
BSAP	B-cell-specific transcription factor
CD	Cluster of differentiation
CD15	CD antigen 15 (alpha-(1,3)-fucosyltransferase 4, FUT4)
CD20	CD antigen 20 (B-lymphocyte antigen CD20)
CD27	CD antigen 27 (Tumor necrosis factor receptor superfamily member 7, TNFRSF7)
CD30	CD antigen 30 (tumor necrosis factor receptor superfamily member 8, TNFRSF8)
CD40	CD antigen 40 (Tumor necrosis factor receptor superfamily member 5, TNFRSF5)
CD45	CD antigen 45 (Receptor-type tyrosine-protein phosphatase C, PTPRC)
CD80	CD antigen 80 (T-lymphocyte activation antigen CD80)
CD86	CD antigen 86 (T-lymphocyte activation antigen CD86)
CD95	CD antigen 95 (Tumor necrosis factor receptor superfamily member 6, TNFRSF6, or Apoptosis-mediating surface antigen FAS)
CD125	CD antigen 125 (Cytotoxic T-lymphocyte protein 4, CTLA4)
cHL	Classical Hodgkin lymphoma
COVID-19	Coronavirus Disease 2019
CTLA4	T-lymphocyte associated protein 4 (CD antigen 125, CD125)
DLBCL	Diffuse large B-cell lymphoma
EBV	Epstein–Barr virus
FL	Follicular lymphoma
FUT4	Alpha-(1,3)-fucosyltransferase 4 (CD antigen 15, CD15)
GCP2	Gamma-tubulin complex component 2
GCP3	Gamma-tubulin complex component 3
GGT1	Gamma-glutamyl transferase 1
HRS	Hodgkin or Reed–Sternberg cells
IRF4	Interferon regulatory factor 4
JAK	Janus kinase 1
mABs	Monoclonal antibodies
LMP-1	Latent oncogenic membrane protein 1
M	Membrane protein of SARS-CoV-2
MMAE	Monomethyl auristatin E
MS4A1	Membrane-spanning 4-domains subfamily A member 1
MT	Microtubule
PAX5	Paired box protein Pax-5
MUM1	Multiple myeloma oncogene 1
PD-1	Programmed cell death-1
PD-L1	Programmed cell death 1 ligand 1
PD-L2	Programmed cell death 1 ligand 2
PTPRC	Receptor-type tyrosine-protein phosphatase C (CD antigen 45, CD45)
SARS-CoV-2	Severe acute respiratory coronavirus 2
RBD	Receptor binding domain of spike protein
S	Spike protein of SARS-CoV-2
STAT	Signal transducer and activator of transcription
TNFRSF5	Tumor necrosis factor receptor superfamily member 5 (CD antigen 40, CD40)
TNFRSF6	Tumor necrosis factor receptor superfamily member 6 (CD antigen 95, CD95)
TNFRSF7	Tumor necrosis factor receptor superfamily member 7 (CD antigen 27, CD27)
TNFRSF8	Tumor necrosis factor receptor superfamily member 8 (CD antigen 30)
TRAFs	Tumor necrosis factor receptor-associated factors
TRADD	Tumor necrosis factor receptor type 1-associated DEATH domain protein
TUBG1	Tubulin gamma-1 chain
