# Supplementary material for: Potential Molecular Mechanisms of Rare Anti-Tumor Immune Response by SARS-CoV-2 in Isolated Cases of Lymphomas
Source: Viruses. 2021 Sep 25;13(10):1927. doi: 10.3390/v13101927 (PMC8539762; doi:10.3390/v13101927)

Fig S1: The Ramachandran plot of the refined CD30 structure.

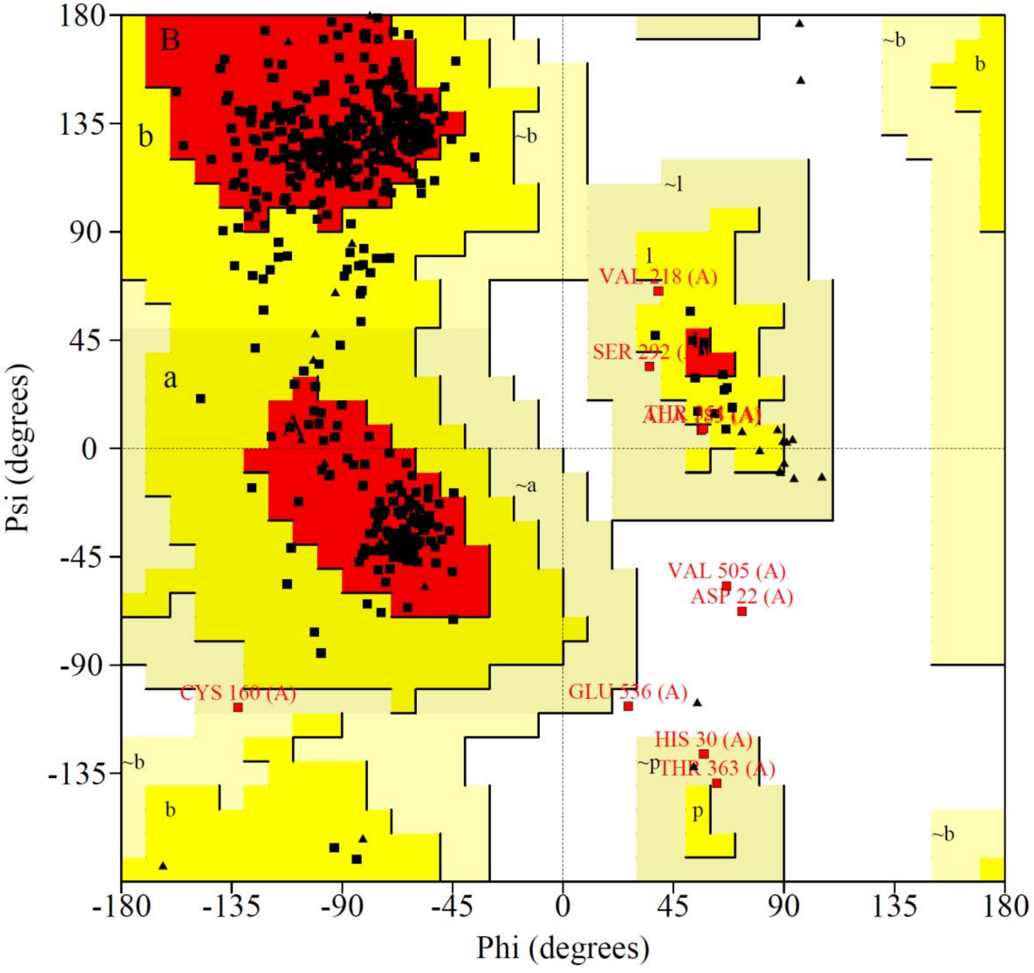

Plot statistics

Residues in most favoured regions [A,B,L]	428	87.0%
Residues in additional allowed regions [a,b,l,p]	54	11.0%
Residues in generously allowed regions [~a,~b,~l,~p]	7	1.4%
Residues in disallowed regions	3	0.6%
	----	------
Number of non-glycine and non-proline residues	492	100.0%
Number of end-residues (excl. Gly and Pro)	2	
Number of glycine residues (shown as triangles)	34	
Number of proline residues	67	

Total number of residues	595	

Fig S2: The Ramachandran plot of the refined CD15 structure.

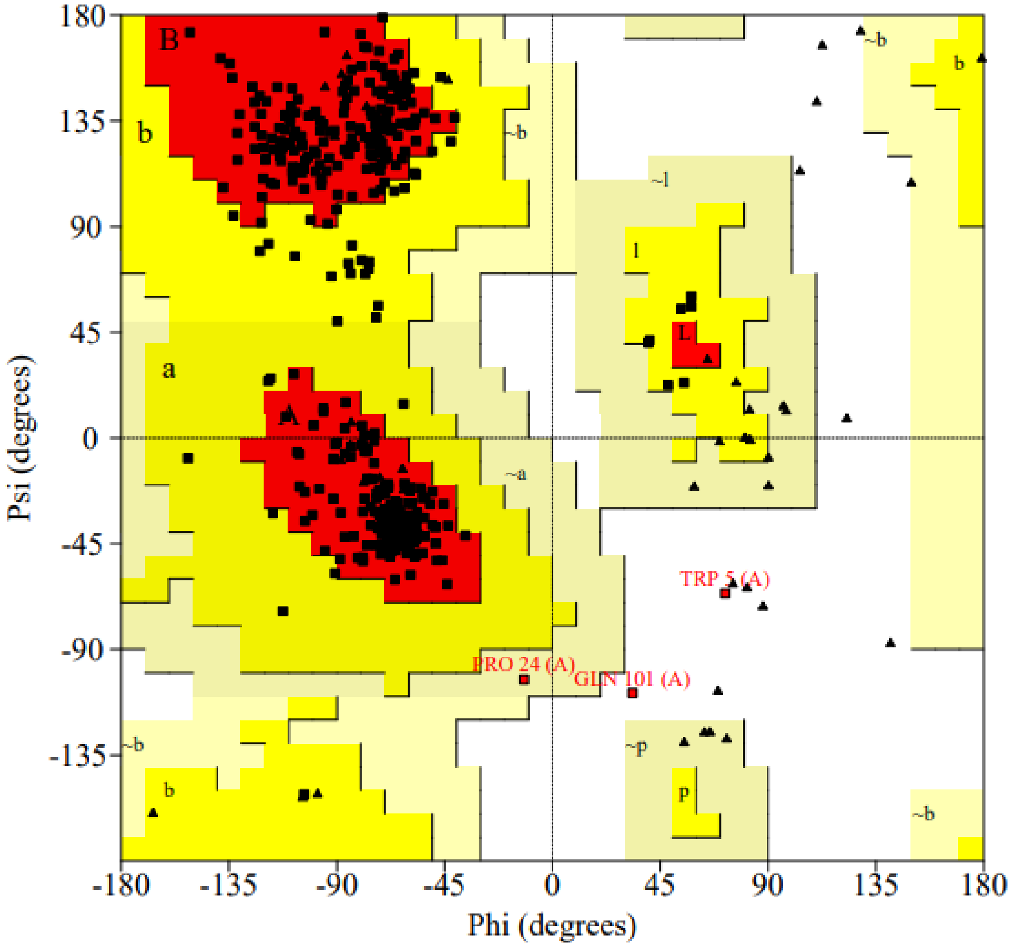

Plot statistics

Residues in most favoured regions [A,B,L]	393	92.0%
Residues in additional allowed regions [a,b,l,p]	32	7.5%
Residues in generously allowed regions [~a,~b,~l,~p]	0	0.0%
Residues in disallowed regions	2	0.5%
	----	------
Number of non-glycine and non-proline residues	427	100.0%
Number of end-residues (excl. Gly and Pro)	2	
Number of glycine residues (shown as triangles)	47	
Number of proline residues	54	

Total number of residues	530	

Fig S3: The Ramachandran plot of the refined SARS-CoV-2 M protein structure.

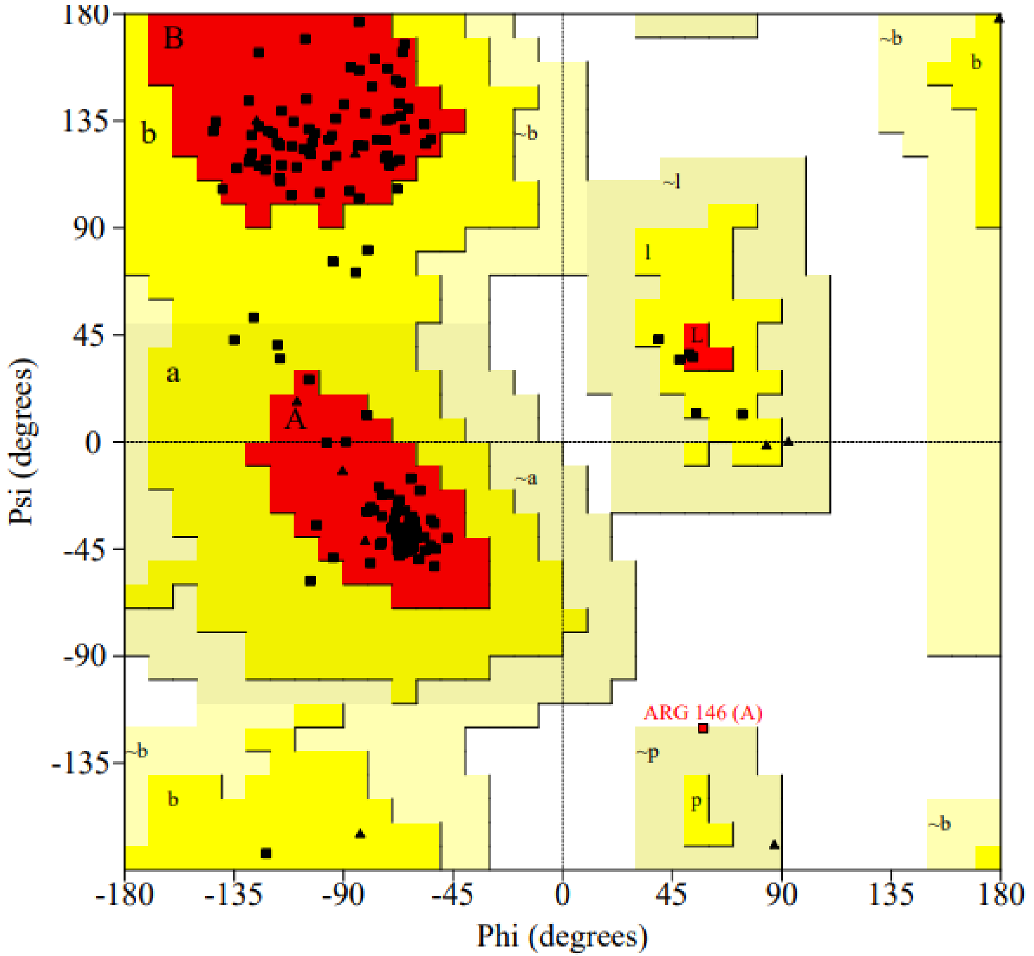

Plot statistics

Residues in most favoured regions [A,B,L]	186	92.5%
Residues in additional allowed regions [a,b,l,p]	14	7.0%
Residues in generously allowed regions [~a,~b,~l,~p]	1	0.5%
Residues in disallowed regions	0	0.0%
	----	------
Number of non-glycine and non-proline residues	201	100.0%
Number of end-residues (excl. Gly and Pro)	2	
Number of glycine residues (shown as triangles)	14	
Number of proline residues	5	

Total number of residues	222	

Fig S4 : Refined 3D model EBV_LMP1

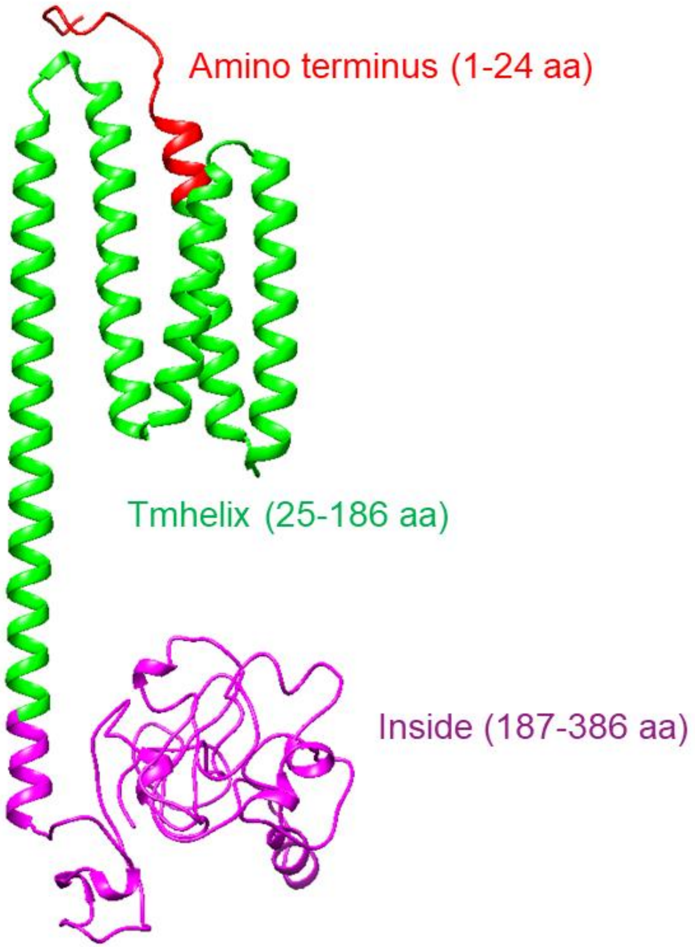

Fig S5: The Ramachandran plot of the refined **3D model EBV LMP1** structure.

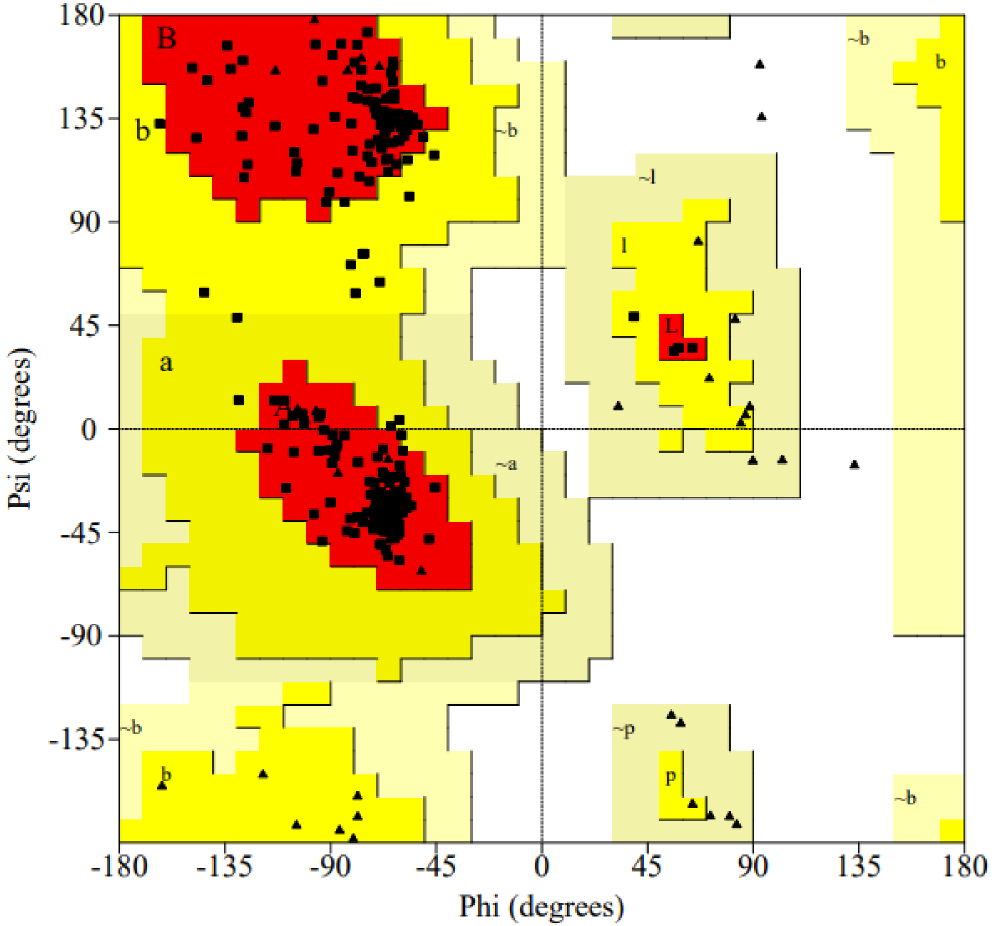

Plot statistics

Residues in most favoured regions [A,B,L]	290	96.0%
Residues in additional allowed regions [a,b,l,p]	12	4.0%
Residues in generously allowed regions [~a,~b,~l,~p]	0	0.0%
Residues in disallowed regions	0	0.0%
	----	------
Number of non-glycine and non-proline residues	302	100.0%
Number of end-residues (excl. Gly and Pro)	2	
Number of glycine residues (shown as triangles)	46	
Number of proline residues	36	

Total number of residues	386	

Fig S6: The Ramachandran plot of the RaptorX 3D model EBV LMP1 structure.

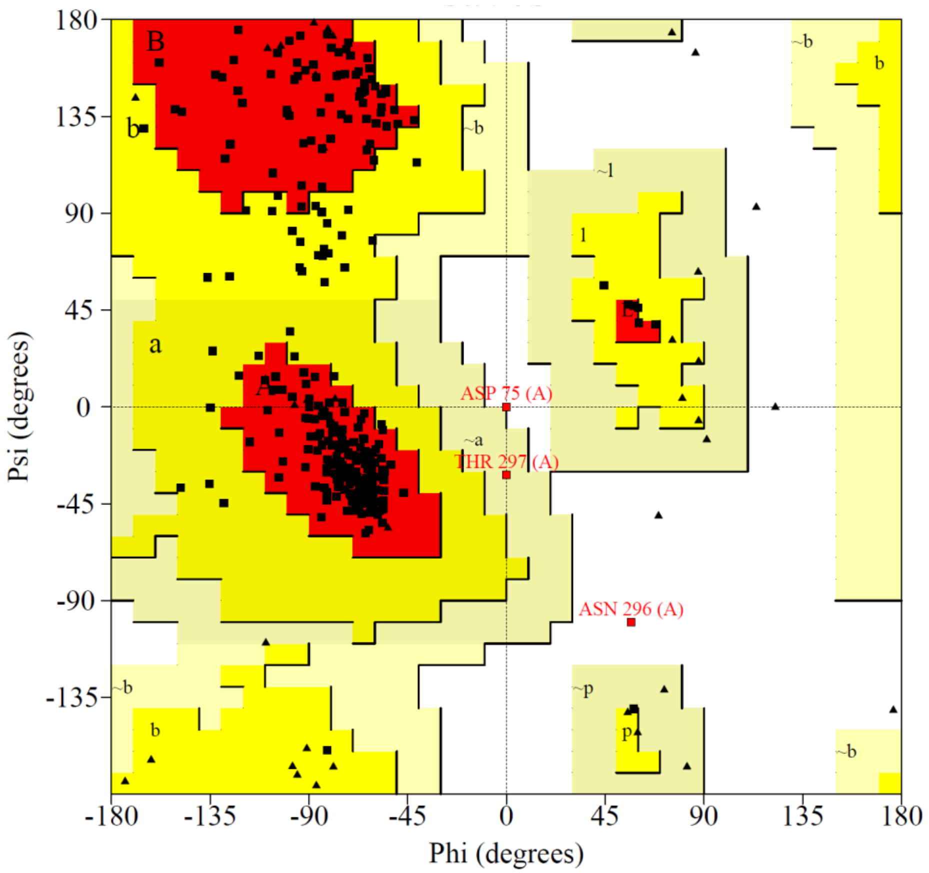

Plot statistics

Residues in most favoured regions [A,B,L]	265	87.7%
Residues in additional allowed regions [a,b,l,p]	34	11.3%
Residues in generously allowed regions [~a,~b,~l,~p]	1	0.3%
Residues in disallowed regions	2	0.7%
	----	------
Number of non-glycine and non-proline residues	302	100.0%
Number of end-residues (excl. Gly and Pro)	2	
Number of glycine residues (shown as triangles)	46	
Number of proline residues	36	

Total number of residues	386	

Based on an analysis of 118 structures of resolution of at least 2.0 Angstroms and R-factor no greater than 20%, a good quality model would be expected to have over 90% in the most favoured regions.

Figure S7: 2D representation of Spike-RBD interacting with *h*ACE2 (PDB: 6LZG). Interactions between Spike-RBD (Chain B) and hACE2 (Chain B) provided by the LIGPLOT v.2.2 software. Olive Green lines represent hydrogen bonds, grey semi-circles denote hydrophobic interactions made by hACE2 and Pink semi-circles represent hydrophobic hydrophobic interactions made by the Spike-RBD.

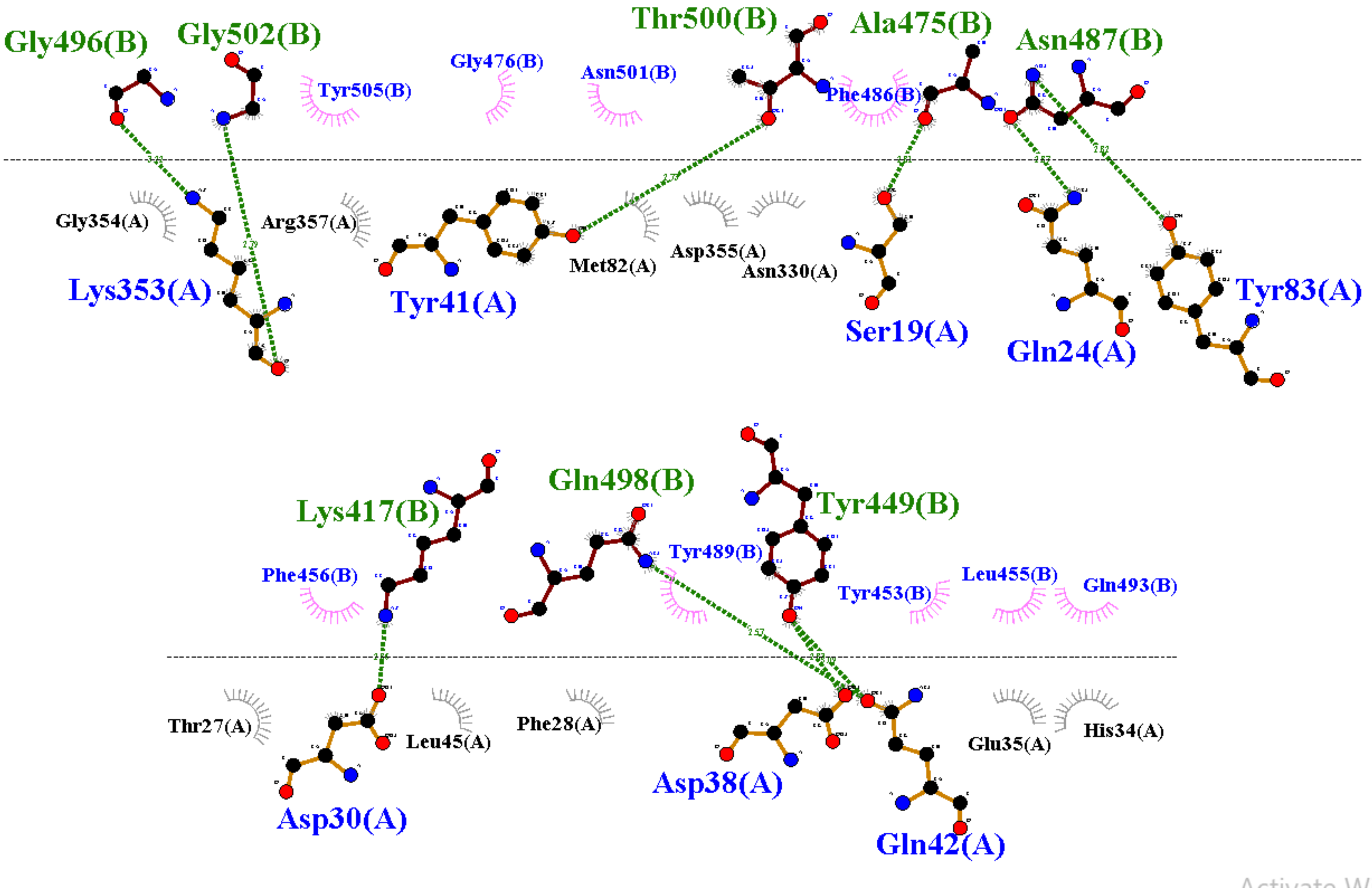

Figure S8: The four combined regions of Spike-RBD.The 3D structure of Spike-RBD(PDB: 6LZG ,Chain B) (Grey) and residues of four important regions R1- Lys417,Tyr449, Tyr453 (Red); R2-Gln498- Tyr502 (Blue); R3-Gln474, Ala475, Phe486, Asn487 (Black); R4-Gly 496, Gln 493 (green)

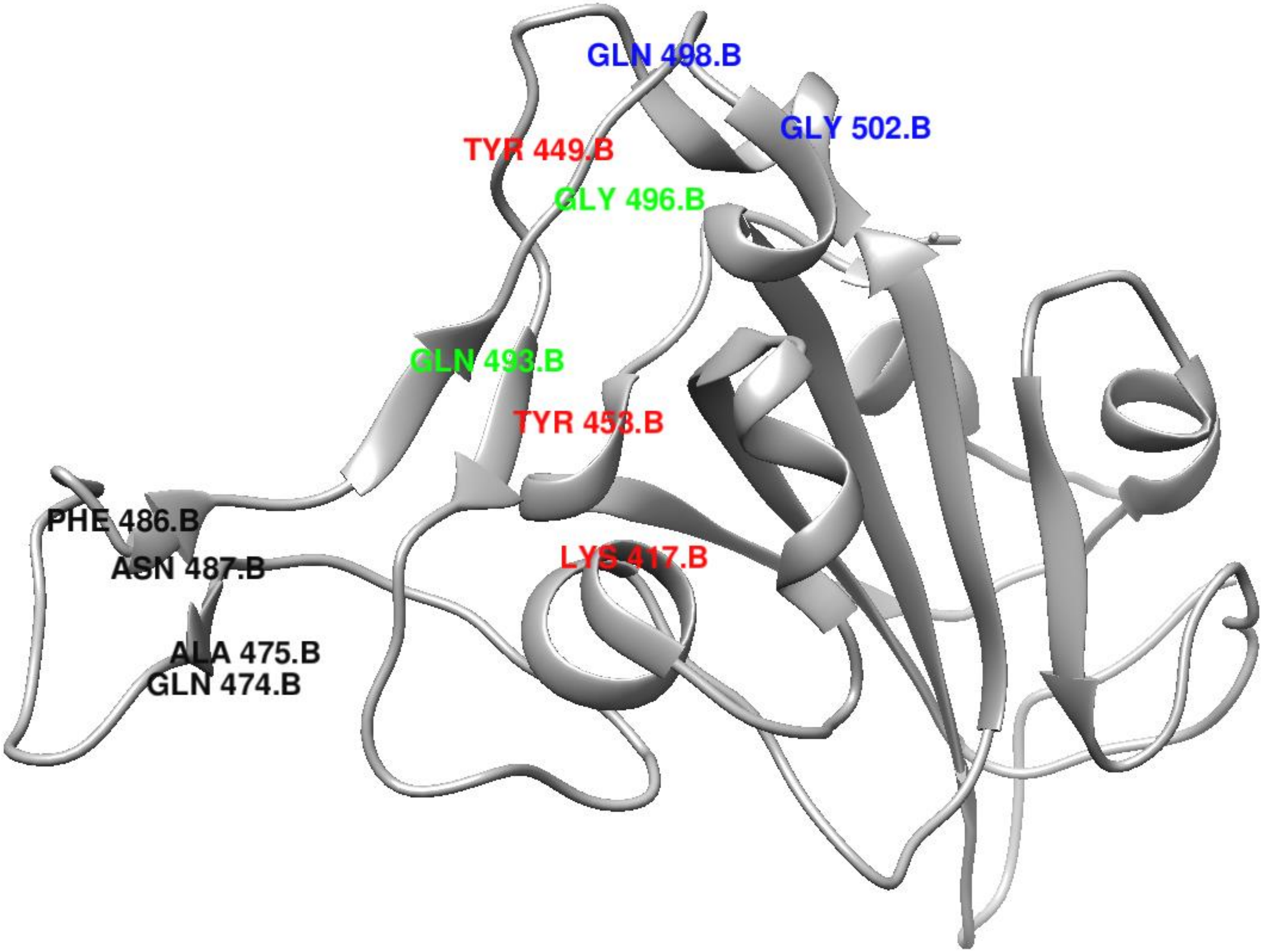

Fig S9: 2D representation of **CD15 (Chain A)** docking residues with Spike RBD (Chain B) Complex 3 , provided by the LIGPLOT v.2.2 software. Olive Green lines represent hydrogen bonds, grey semi-circles denote hydrophobic interactions made by CD15 and Pink semi-circles represent hydrophobic hydrophobic interactions made by the Spike-RBD.

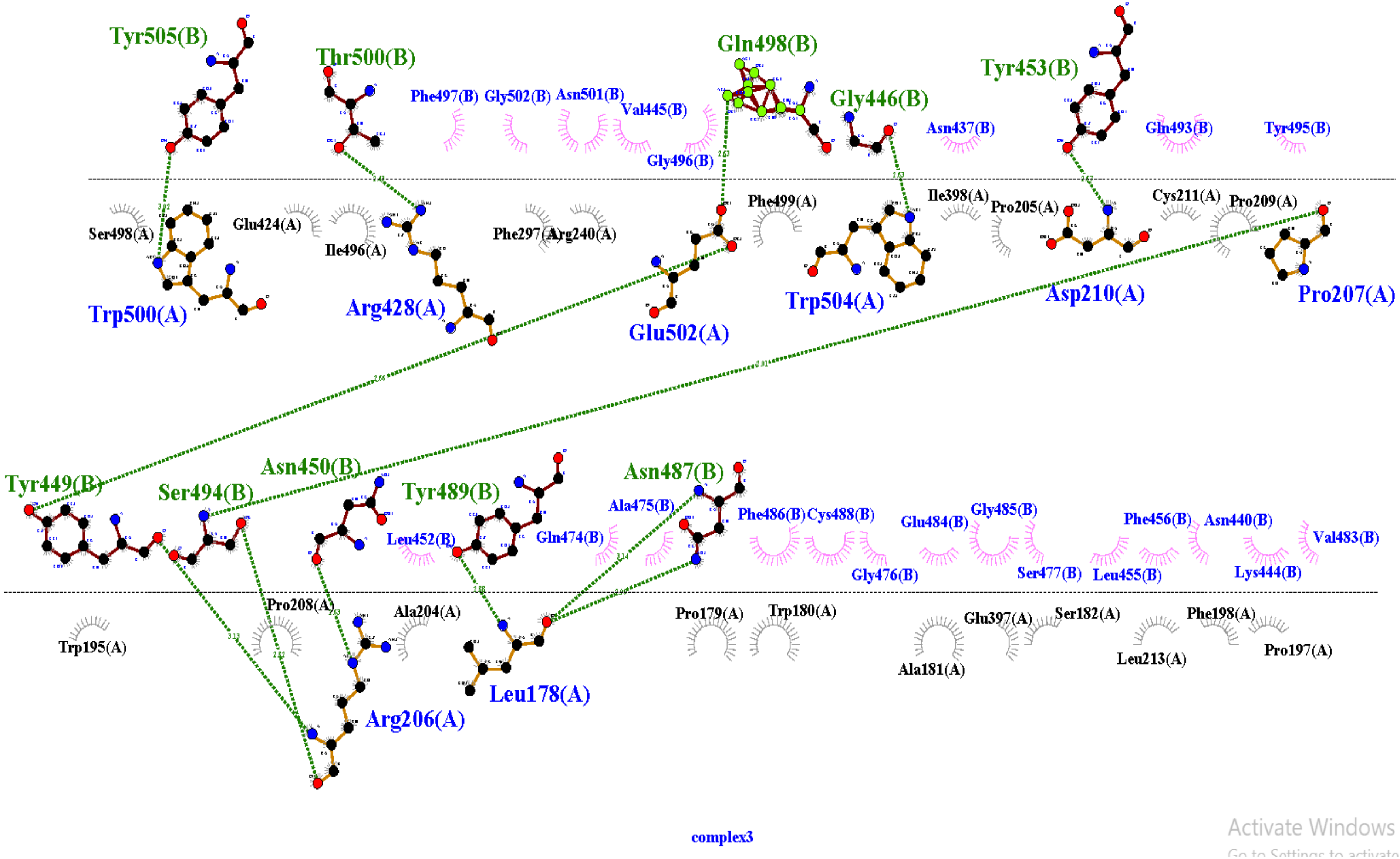

Activate Windows
Go to Settings to activate

Fig S10 2D representation of **CD15 (Chain A)** docking residues with Spike RBD (Chain B), Complex 4 provided by the LIGPLOT v.2.2 software. Olive Green lines represent hydrogen bonds, gray semi-circles denote hydrophobic interactions made by CD15 and Pink semi-circles represent hydrophobic hydrophobic interactions made by the Spike-RBD.

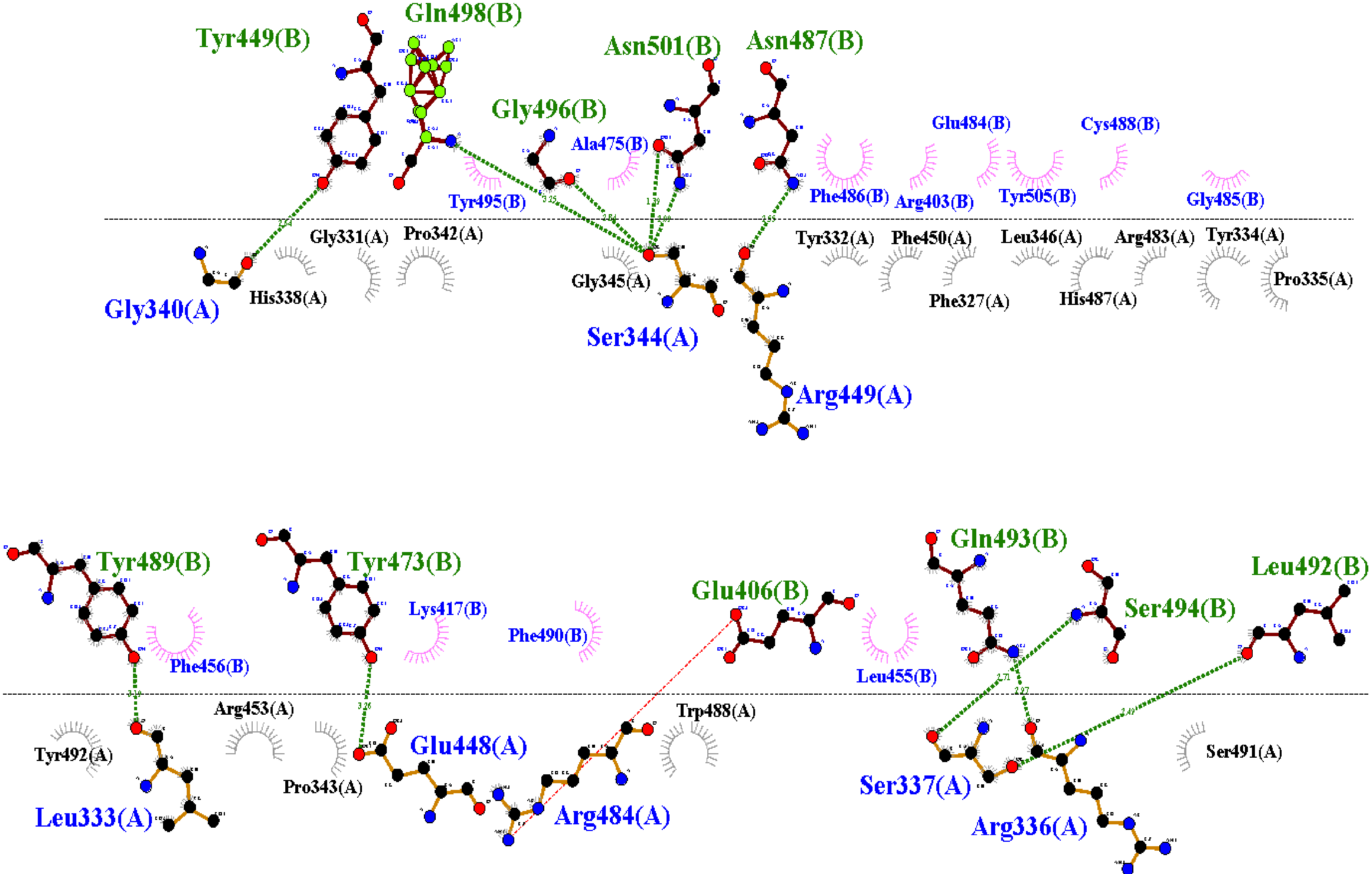

Fig S11 2D representation of **CD15 (Chain A)** docking residues with Spike RBD (Chain B), Complex 6 provided by the LIGPLOT v.2.2 software. Olive Green lines represent hydrogen bonds, grey semi-circles denote hydrophobic interactions made by CD15 and Pink semi-circles represent hydrophobic hydrophobic interactions made by the Spike-RBD.

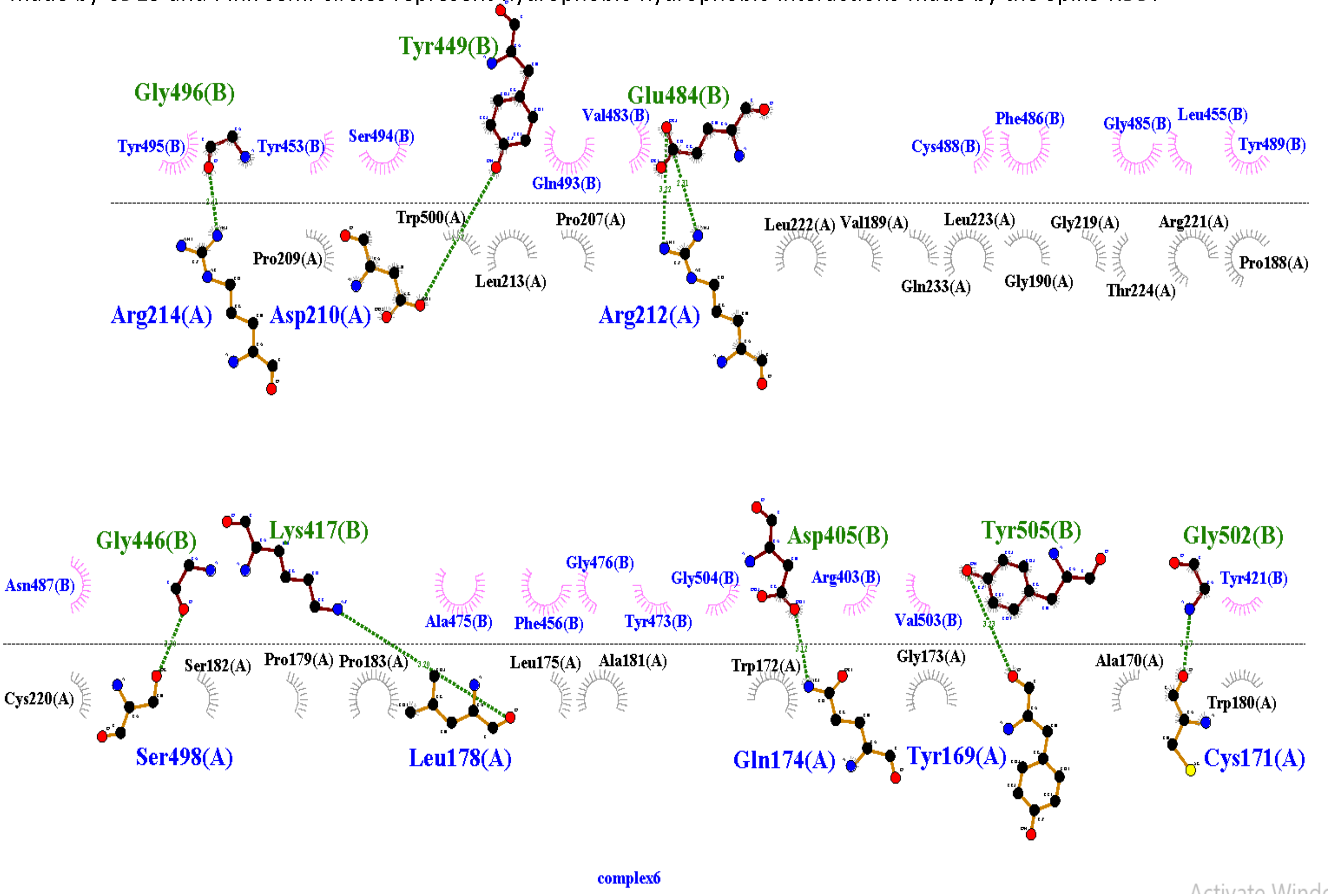

Fig S12 2D representation of **CD27/ TNFRSF7** (Chain A) docking residues with Spike RBD (Chain B), Complex 8, provided by the LIGPLOT v.2.2 software. Olive Green lines represent hydrogen bonds, grey semi-circles denote hydrophobic interactions made by **CD27/ TNFRSF7** and Pink semi-circles represent hydrophobic interactions made by the Spike-RBD.

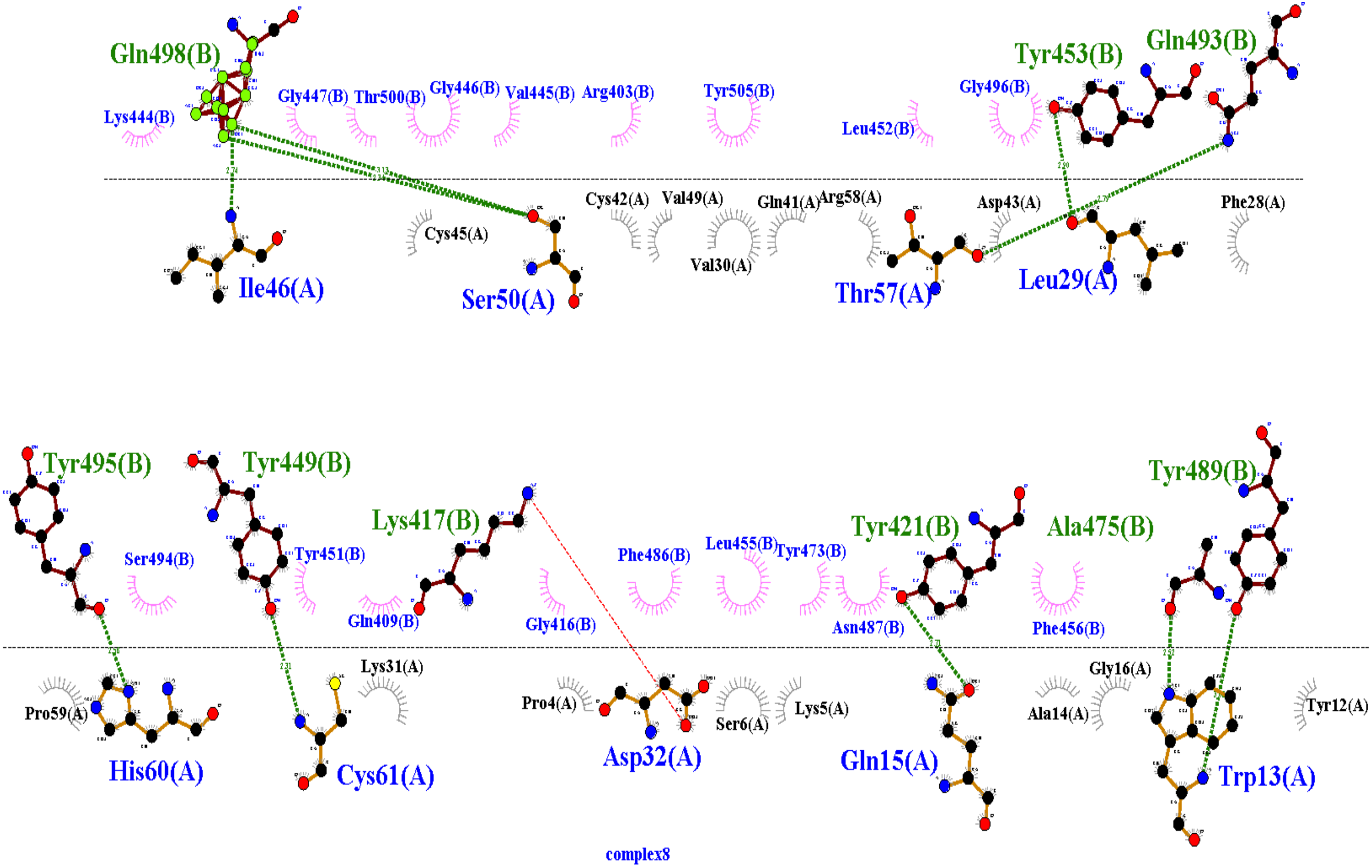

Fig S13: 2D representation of **CD27/ TNFRSF7** (Chain A) docking residues with Spike RBD (Chain B), Complex 9, provided by the LIGPLOT v.2.2 software. Olive Green lines represent hydrogen bonds, grey semi-circles denote hydrophobic interactions made by **CD27/ TNFRSF7** and Pink semi-circles represent hydrophobic interactions made by the Spike-RBD.

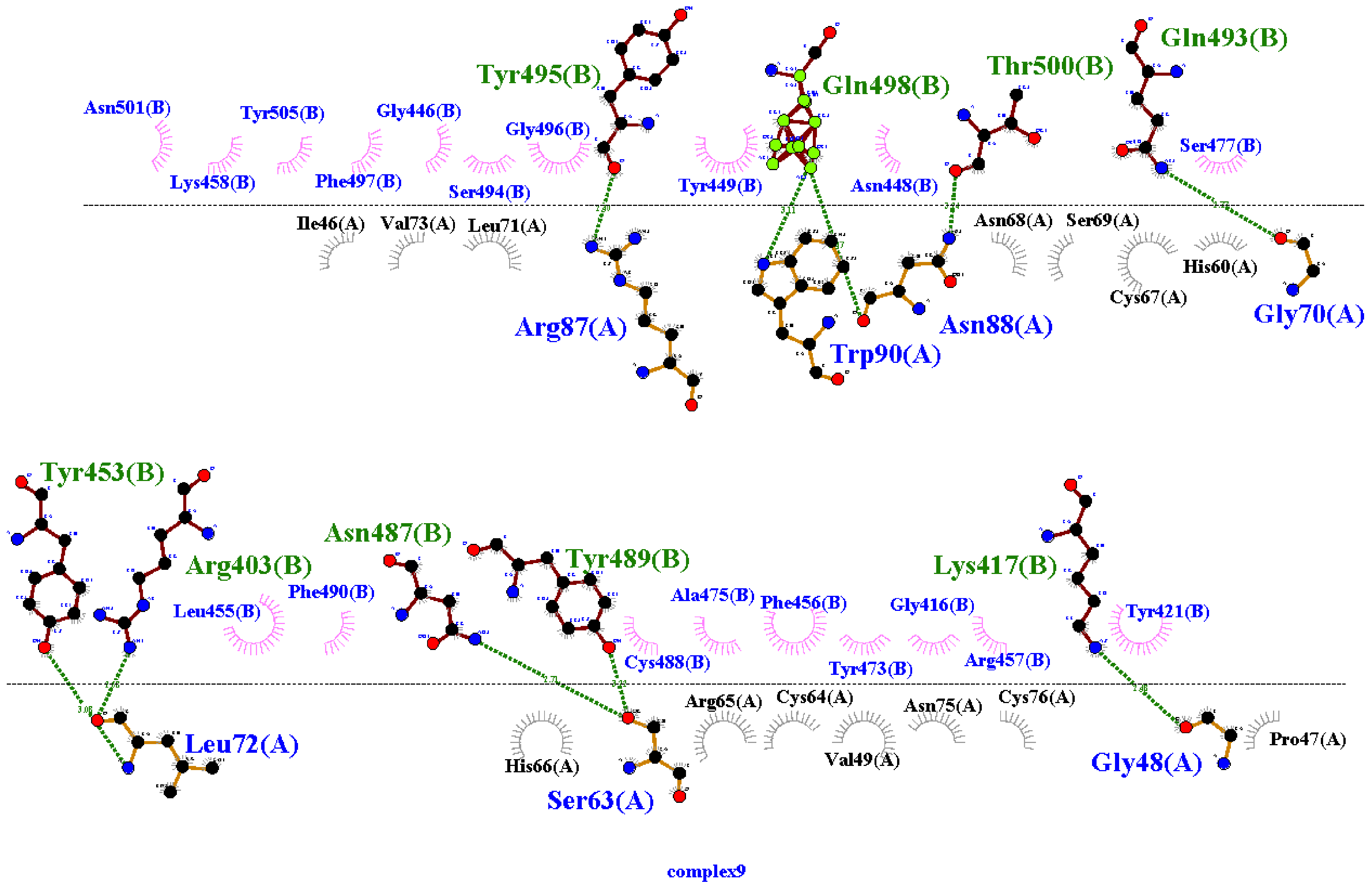

Fig S14: 2D representation of **CD30** (Chain A) docking residues with Spike RBD (Chain B), Complex 5, provided by the LIGPLOT v.2.2 software. Olive Green lines represent hydrogen bonds, grey semi-circles denote hydrophobic interactions made by **CD30** and Pink semi-circles represent hydrophobic interactions made by the Spike-RBD.

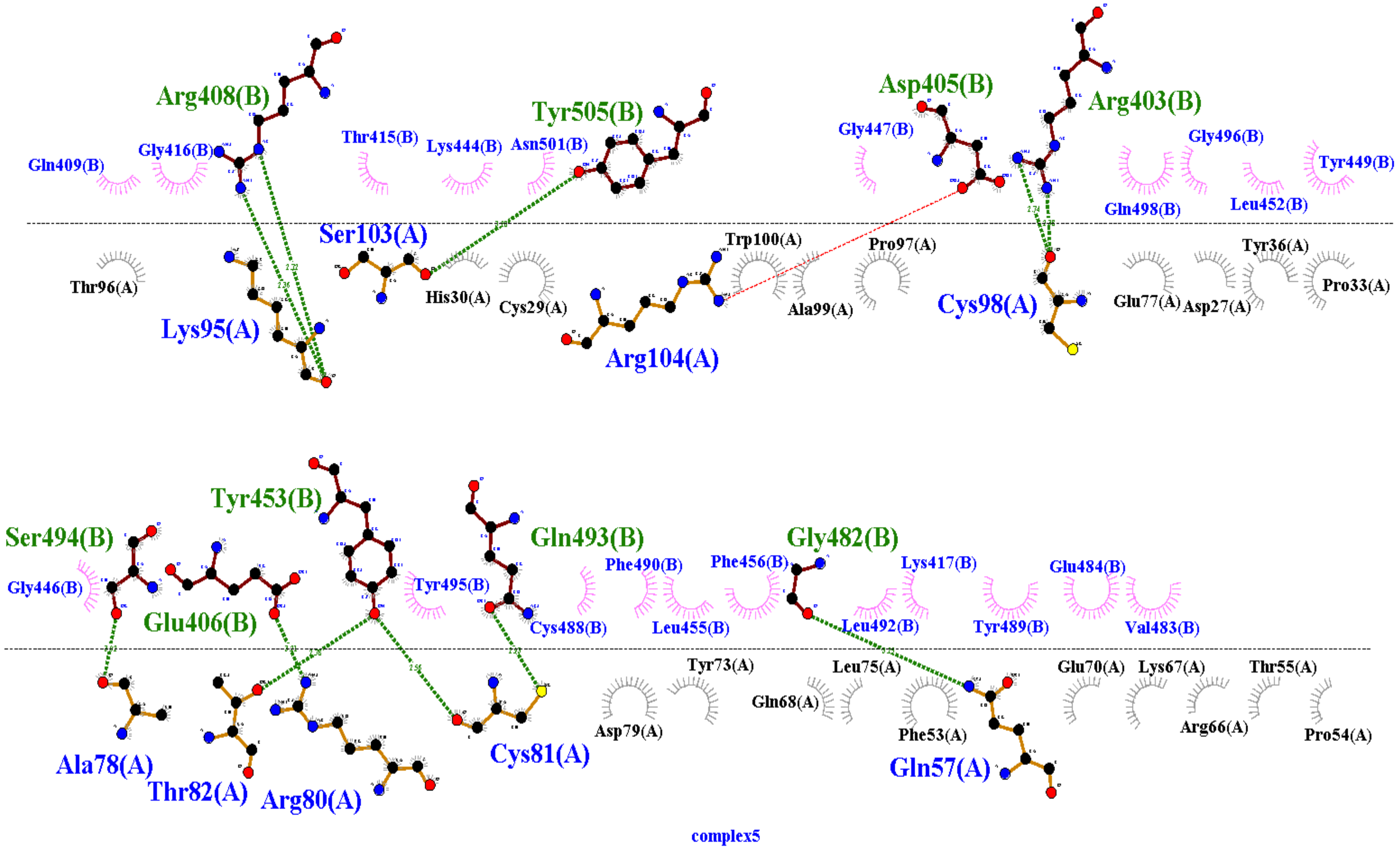

Fig S15: 2D representation of **CD30** (Chain A) docking residues with Spike RBD (Chain B), Complex 3, provided by the LIGPLOT v.2.2 software. Olive Green lines represent hydrogen bonds, grey semi-circles denote hydrophobic interactions made by **CD30** and Pink semi-circles represent hydrophobic interactions made by the Spike-RBD.

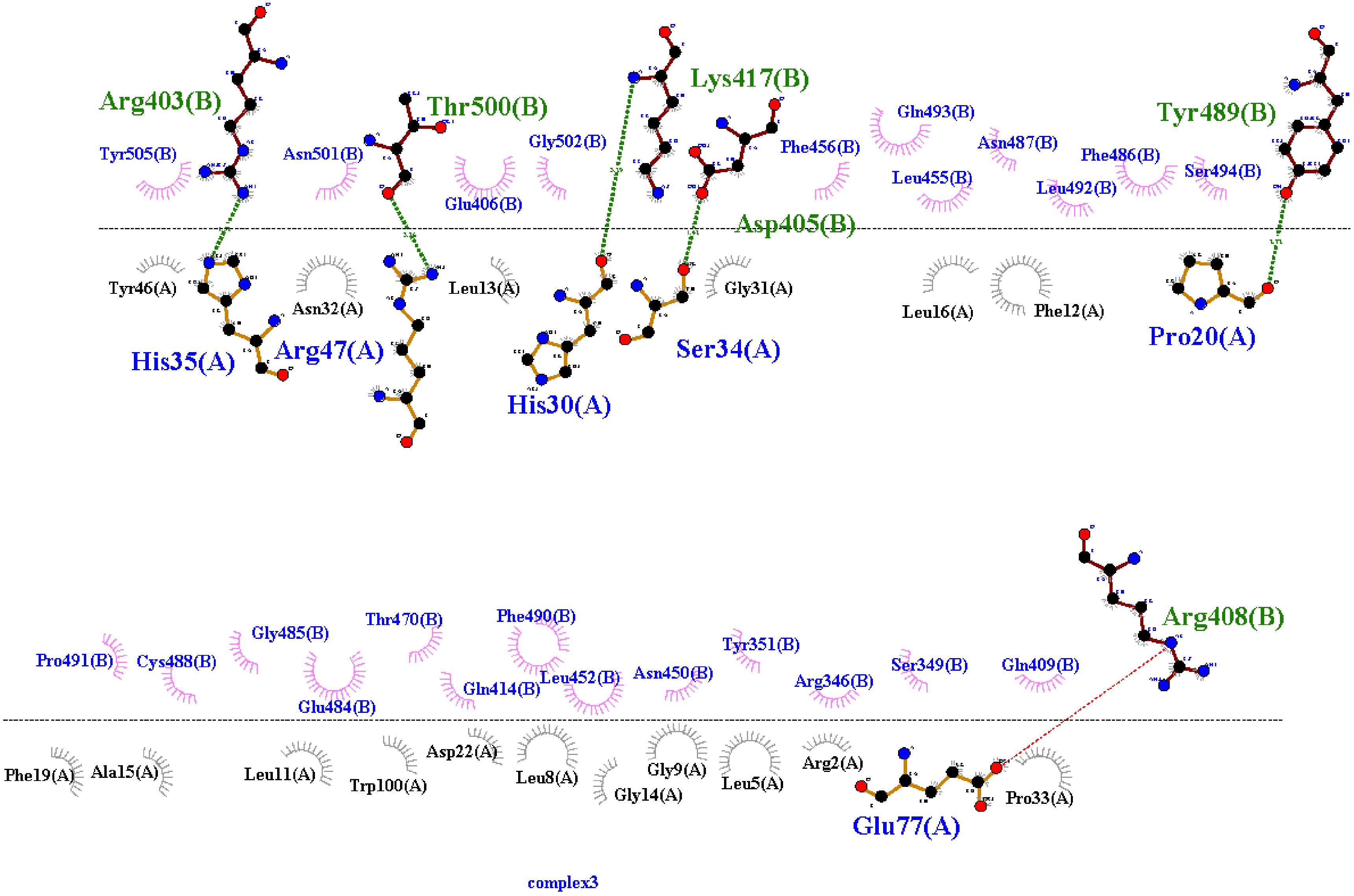

Fig S16: 2D representation of **CD40** (Chain A) docking residues with Spike RBD (Chain B), Complex 8, provided by the LIGPLOT v.2.2 software. Olive Green lines represent hydrogen bonds, grey semi-circles denote hydrophobic interactions made by **CD40** and Pink semi-circles represent hydrophobic interactions made by the Spike-RBD.

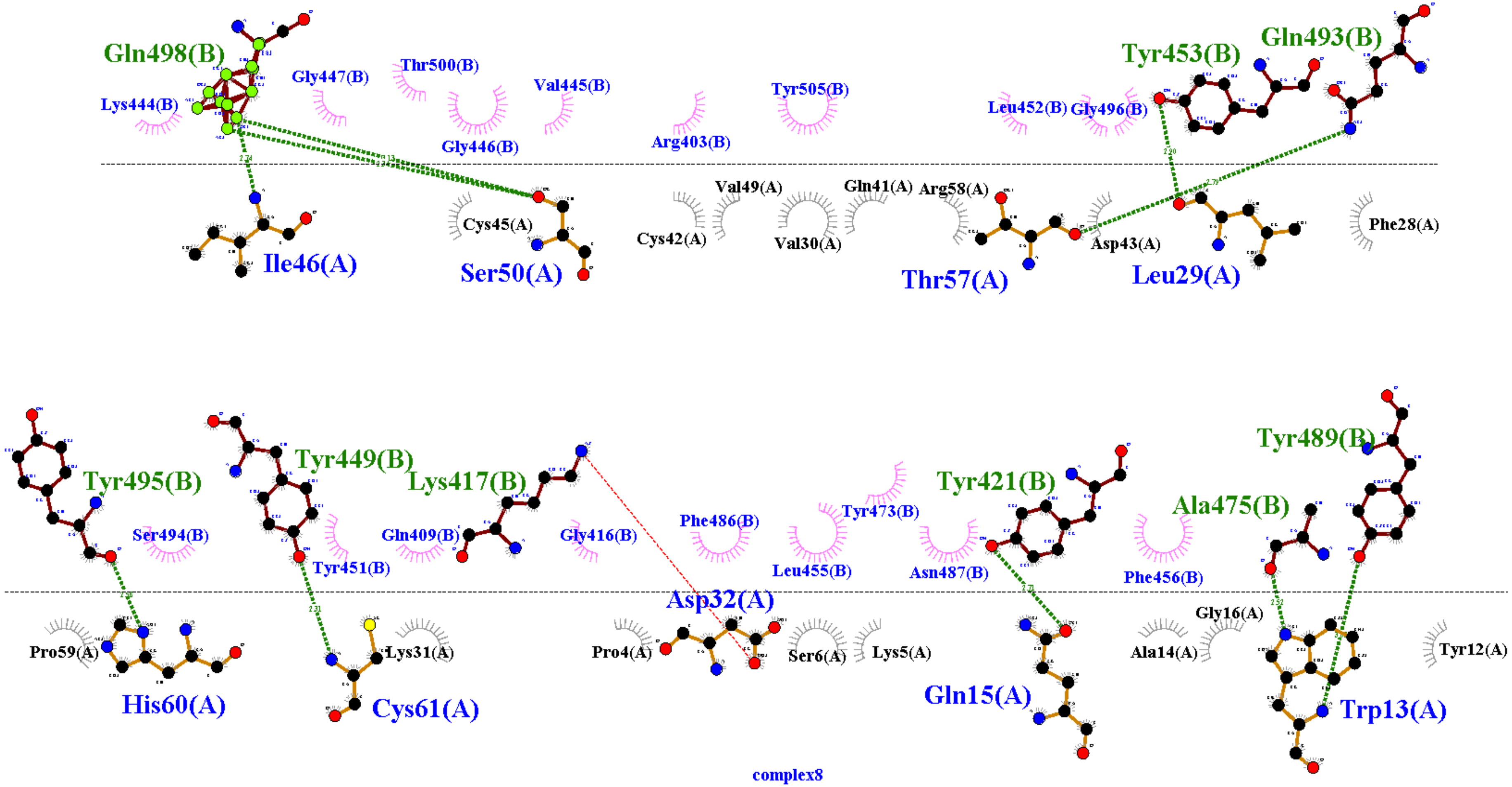

Fig S17: 2D representation of **CD40** (Chain A) docking residues with Spike RBD (Chain B), Complex 9, provided by the LIGPLOT v.2.2 software. Olive Green lines represent hydrogen bonds, grey semi-circles denote hydrophobic interactions made by **CD40** and Pink semi-circles represent hydrophobic interactions made by the Spike-RBD.

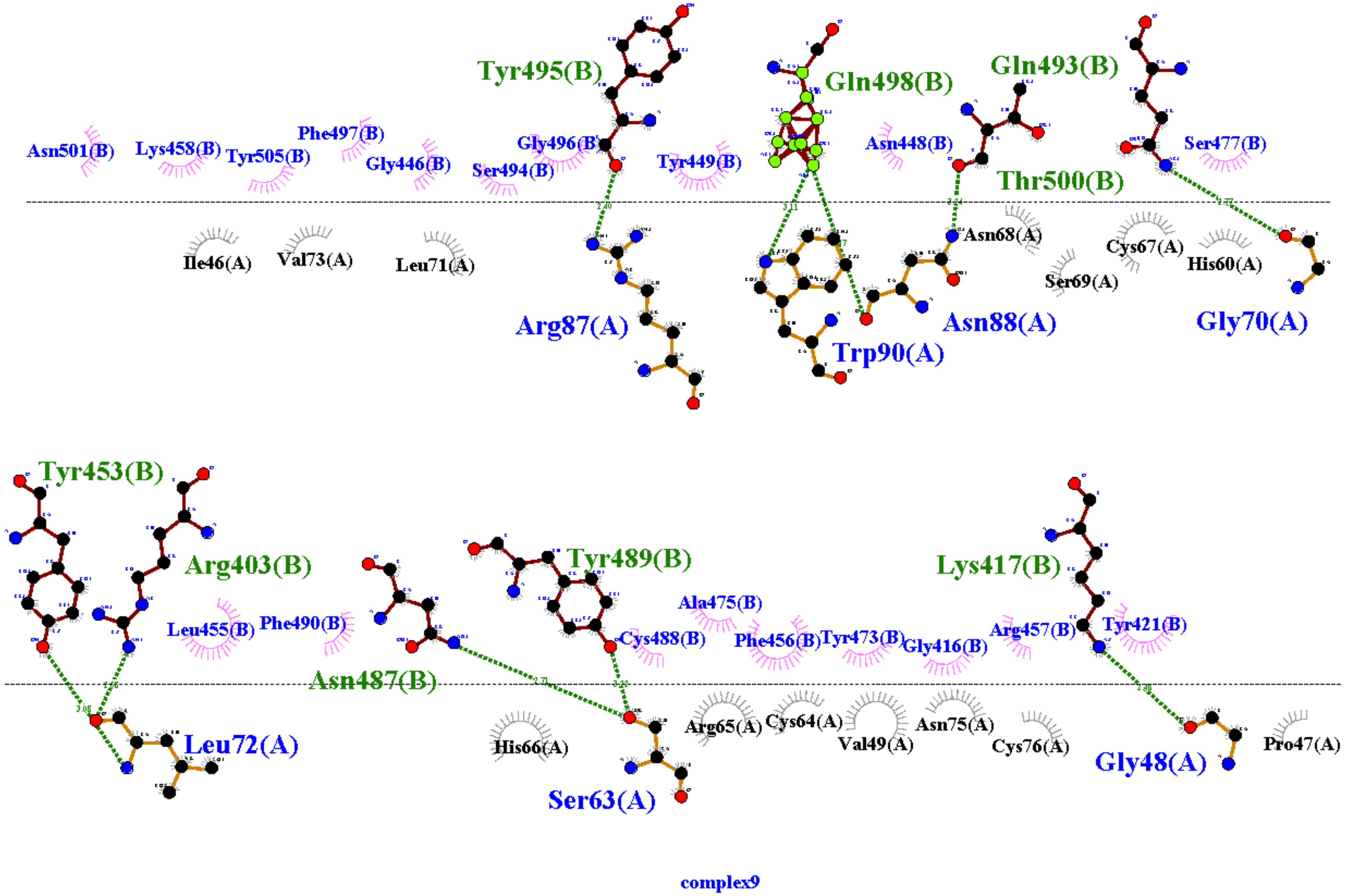

Fig S18: 2D representation of **CD40** (Chain A) docking residues with Spike RBD (Chain B), Complex 3, provided by the LIGPLOT v.2.2 software. Olive Green lines represent hydrogen bonds, grey semi-circles denote hydrophobic interactions made by **CD40** and Pink semi-circles represent hydrophobic interactions made by the Spike-RBD.

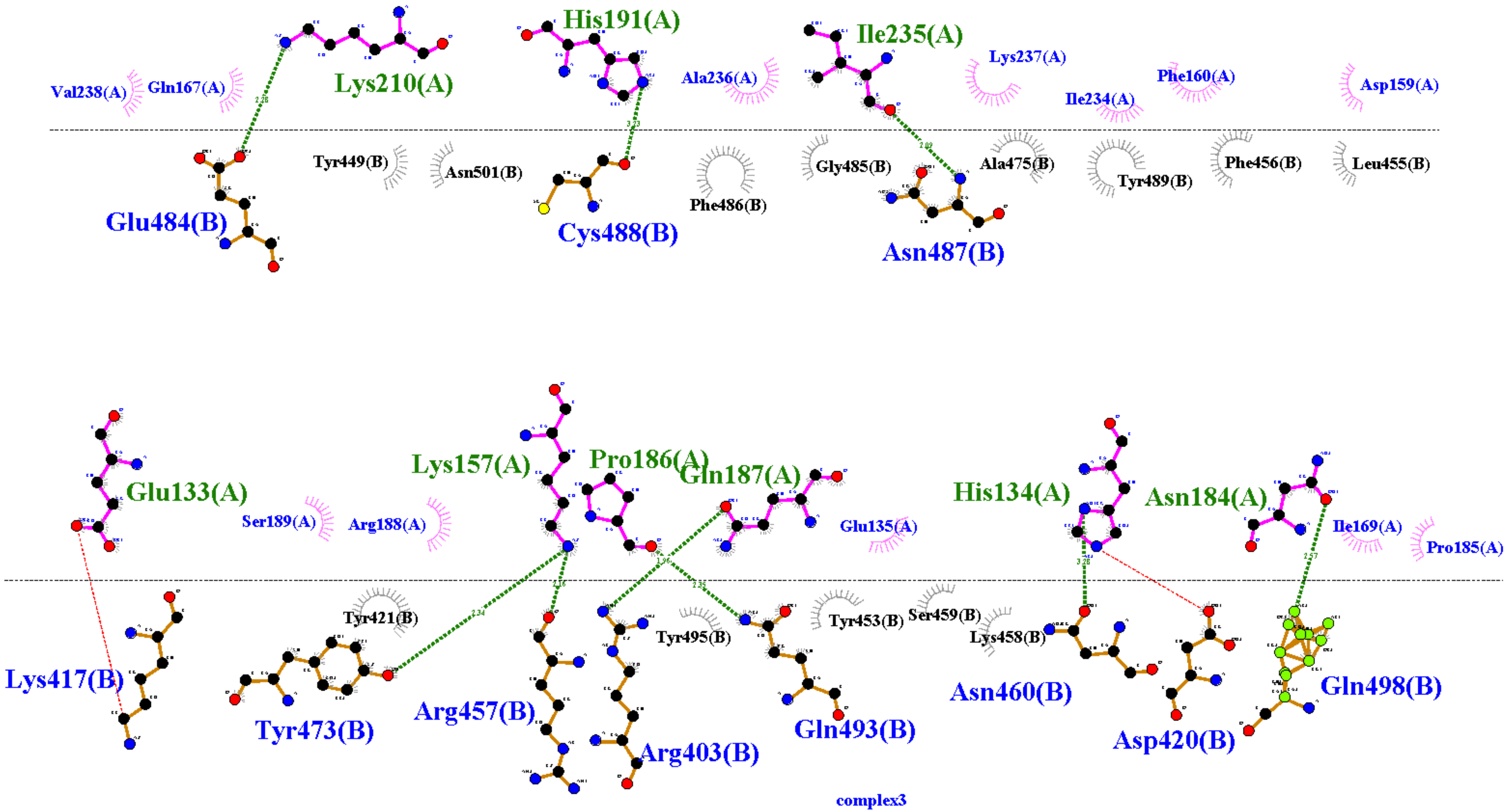

Fig S19: 2D representation of **CD45** (Chain A) docking residues with Spike RBD (Chain B), Complex 8, provided by the LIGPLOT v.2.2 software. Olive Green lines represent hydrogen bonds, grey semi-circles denote hydrophobic interactions made by Spike-RBD and Pink semi-circles represent hydrophobic interactions made by the CD45.

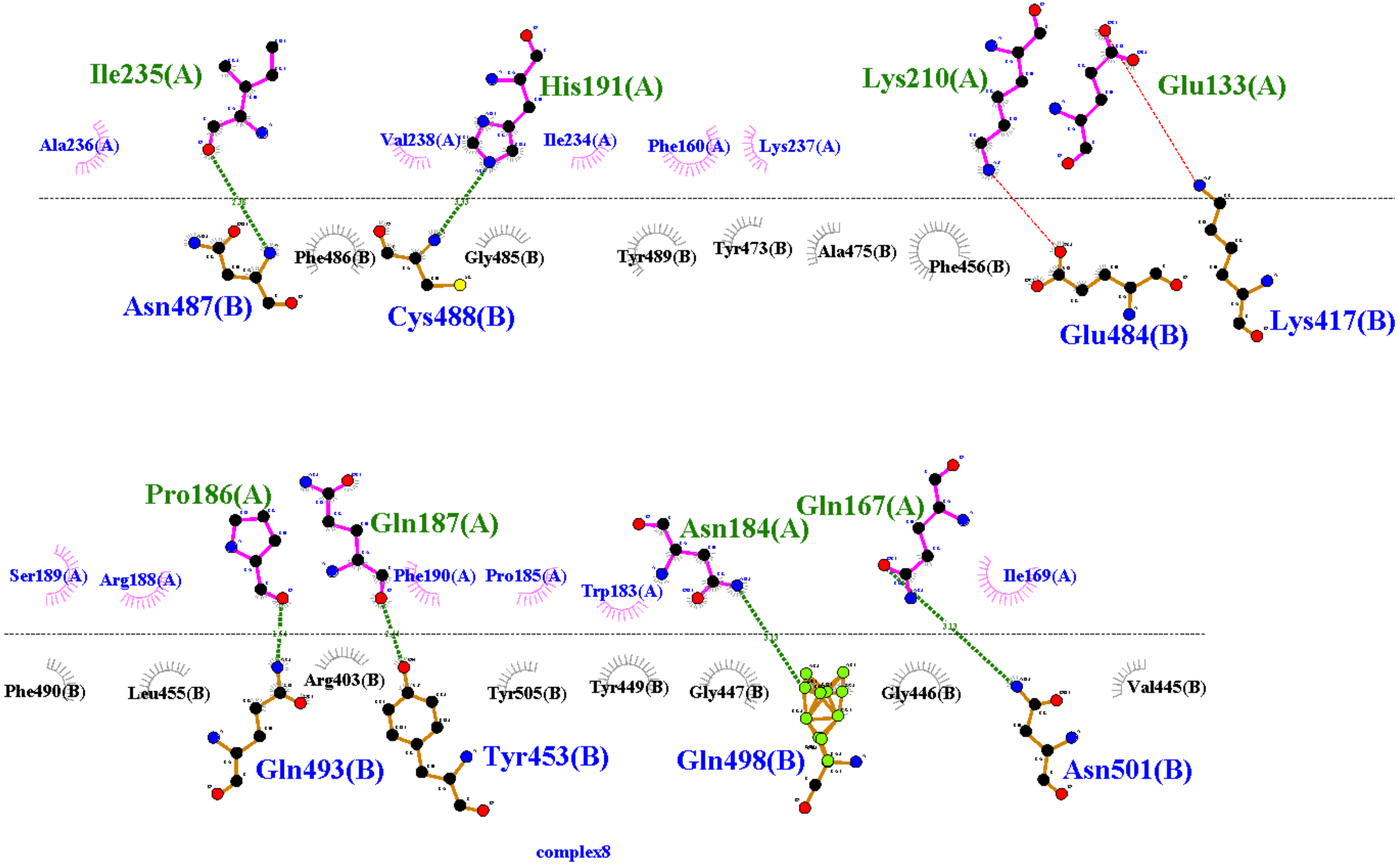

Fig S20: 2D representation of **CD80 (ChainA)** docking residues with Spike RBD (Chain B) Complex 3, provided by the LIGPLOT v.2.2 software. Olive Green lines represent hydrogen bonds, grey semi-circles denote hydrophobic interactions made by CD80 and Pink semi-circles represent hydrophobic interactions made by the Spike RBD

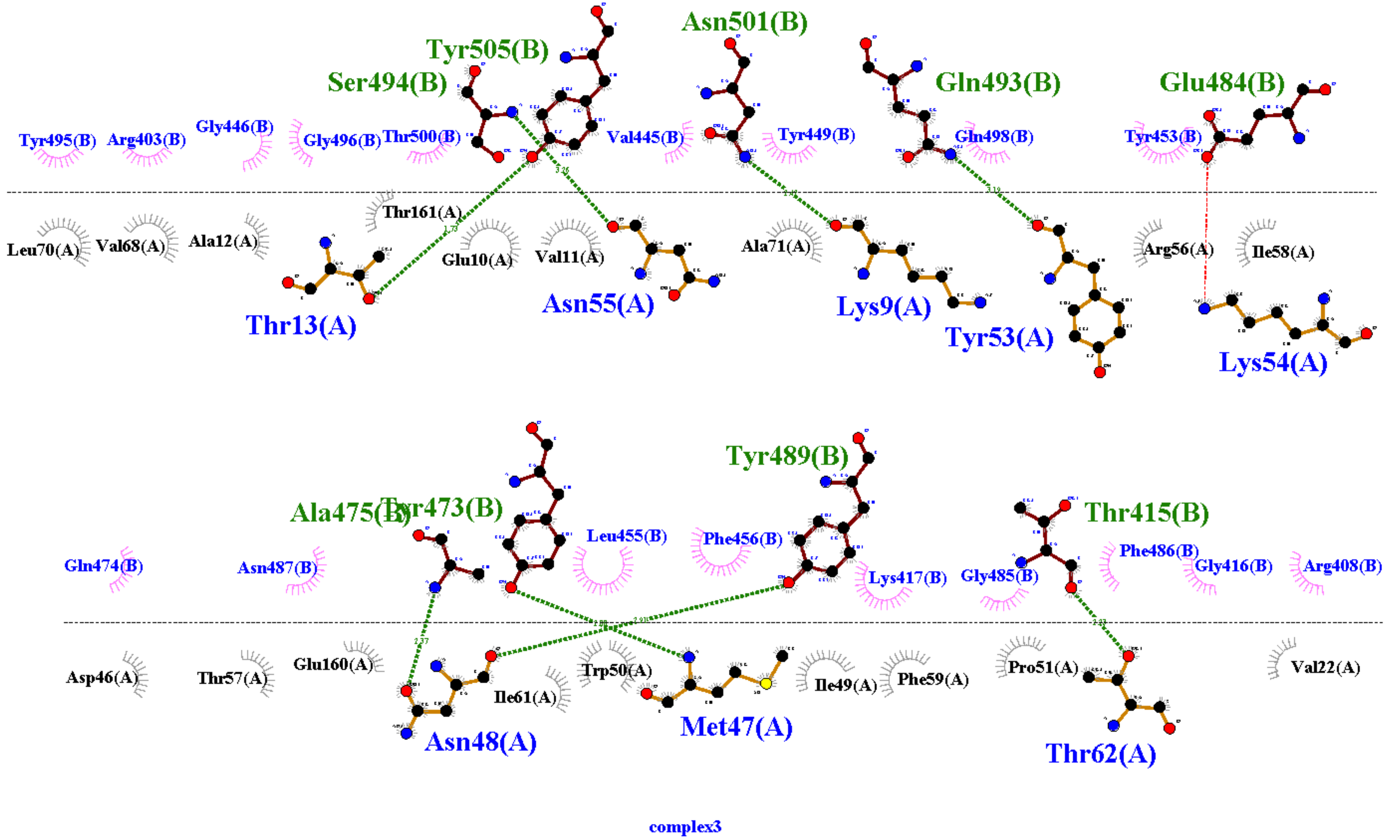

Activate Wi

Fig S21: 2D representation of **CD80 (Chain A)** docking residues with Spike RBD (Chain B) Complex 9, provided by the LIGPLOT v.2.2 software. Olive Green lines represent hydrogen bonds, grey semi-circles denote hydrophobic interactions made by CD80 and Pink semi-circles represent hydrophobic interactions made by the Spike RBD

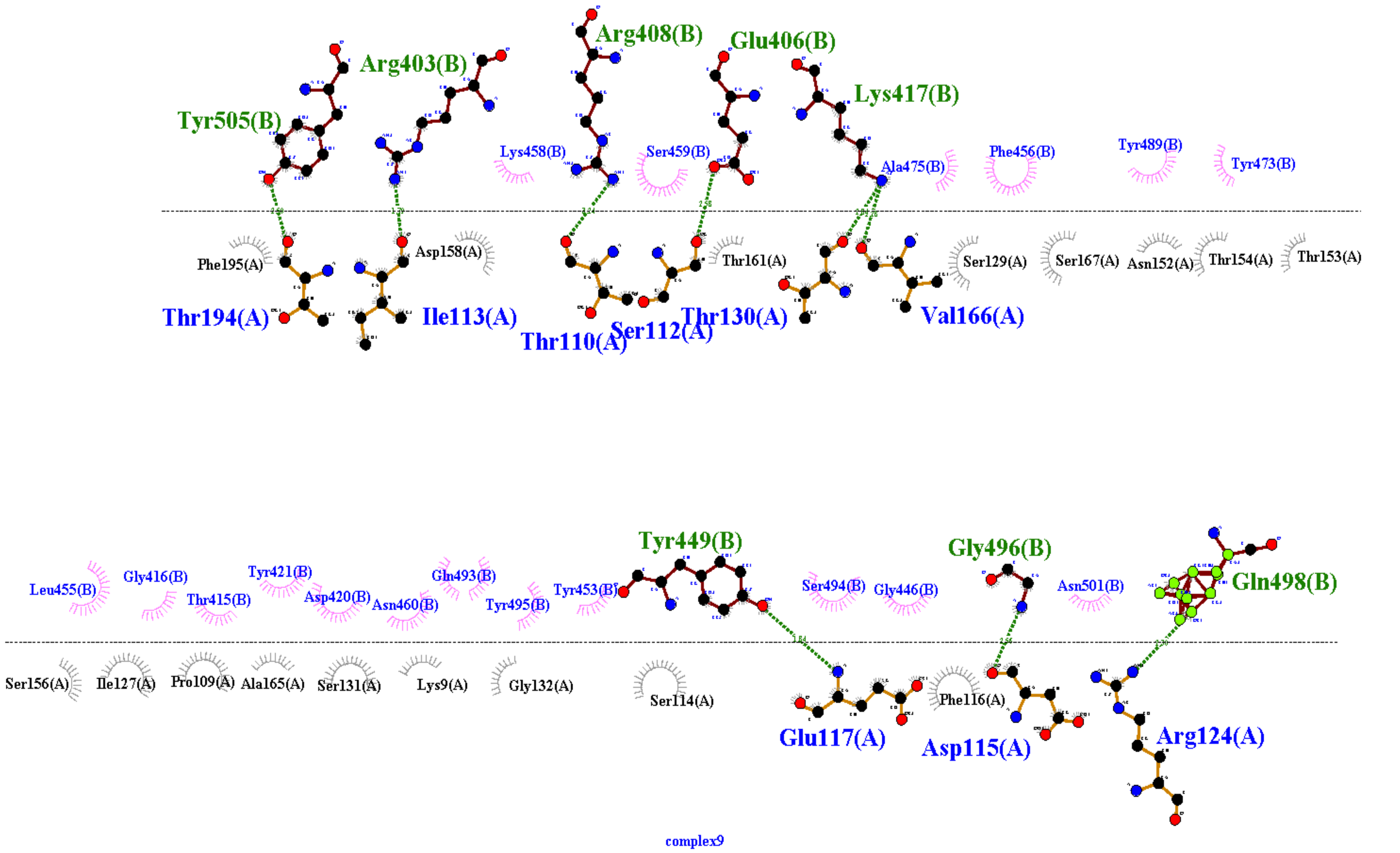

Activate

Fig S22: 2D representation of **CD86** (Chain A) docking residues with Spike RBD Chain B)Complex 2, provided by the LIGPLOT v.2.2 software. Olive Green lines represent hydrogen bonds, grey semi-circles denote hydrophobic interactions made by CD86 and Pink semi-circles represent hydrophobic interactions made by the Spike RBD

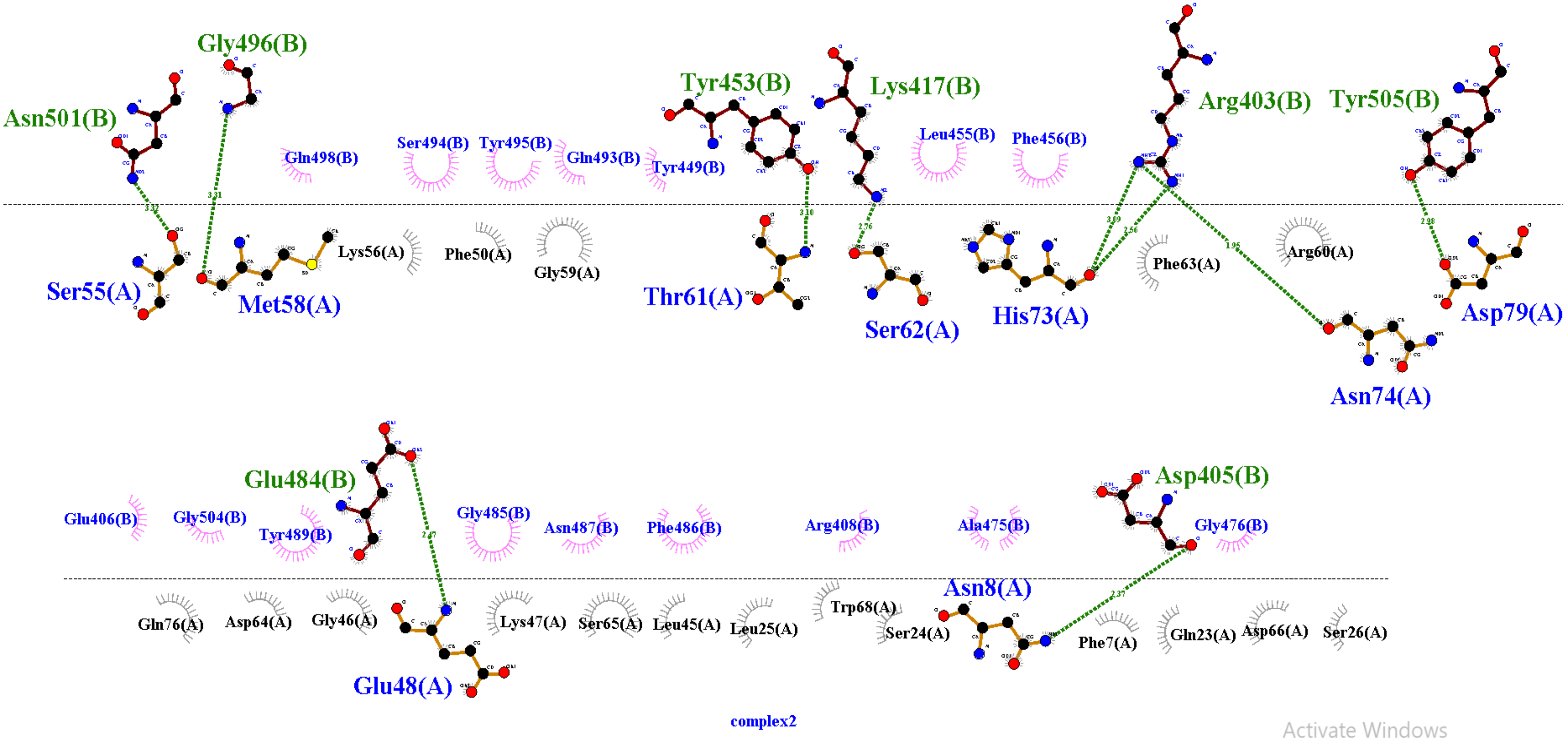

Fig S23: 2D representation of **CD86 (Chain A)** docking residues with Spike RBD (Chain B) Complex 9, provided by the LIGPLOT v.2.2 software. Olive Green lines represent hydrogen bonds, grey semi-circles denote hydrophobic interactions made by CD86 and Pink semi-circles represent hydrophobic interactions made by the Spike RBD .

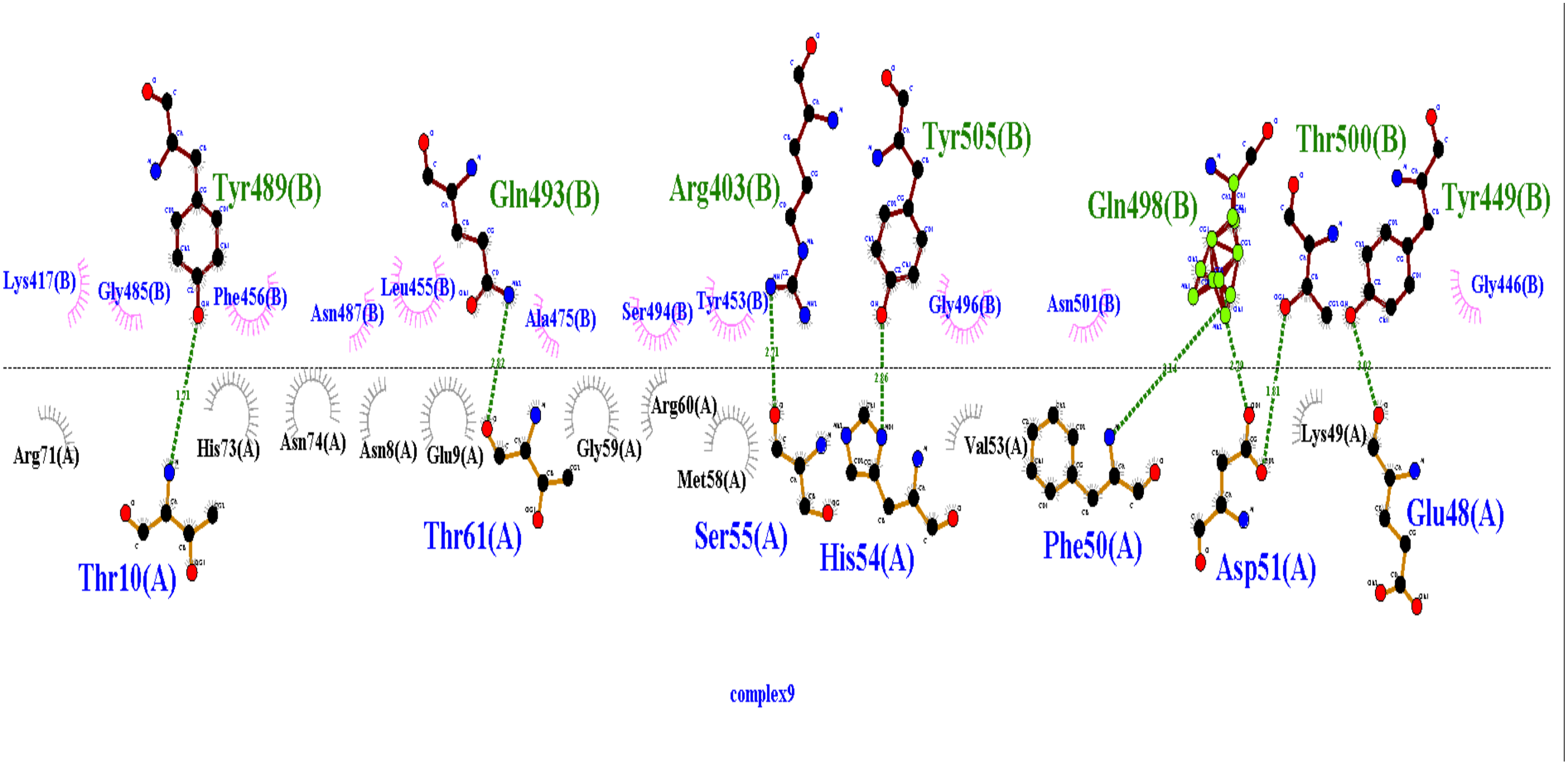

Fig S24: 2D representation of **CD95 (Chain F)** docking residues with Spike RBD (Chain B) Complex 2, provided by the LIGPLOT v.2.2 software. Olive Green lines represent hydrogen bonds, grey semi-circles denote hydrophobic interactions made Spike RBD by and Pink semi-circles represent hydrophobic interactions made by the **CD95**.

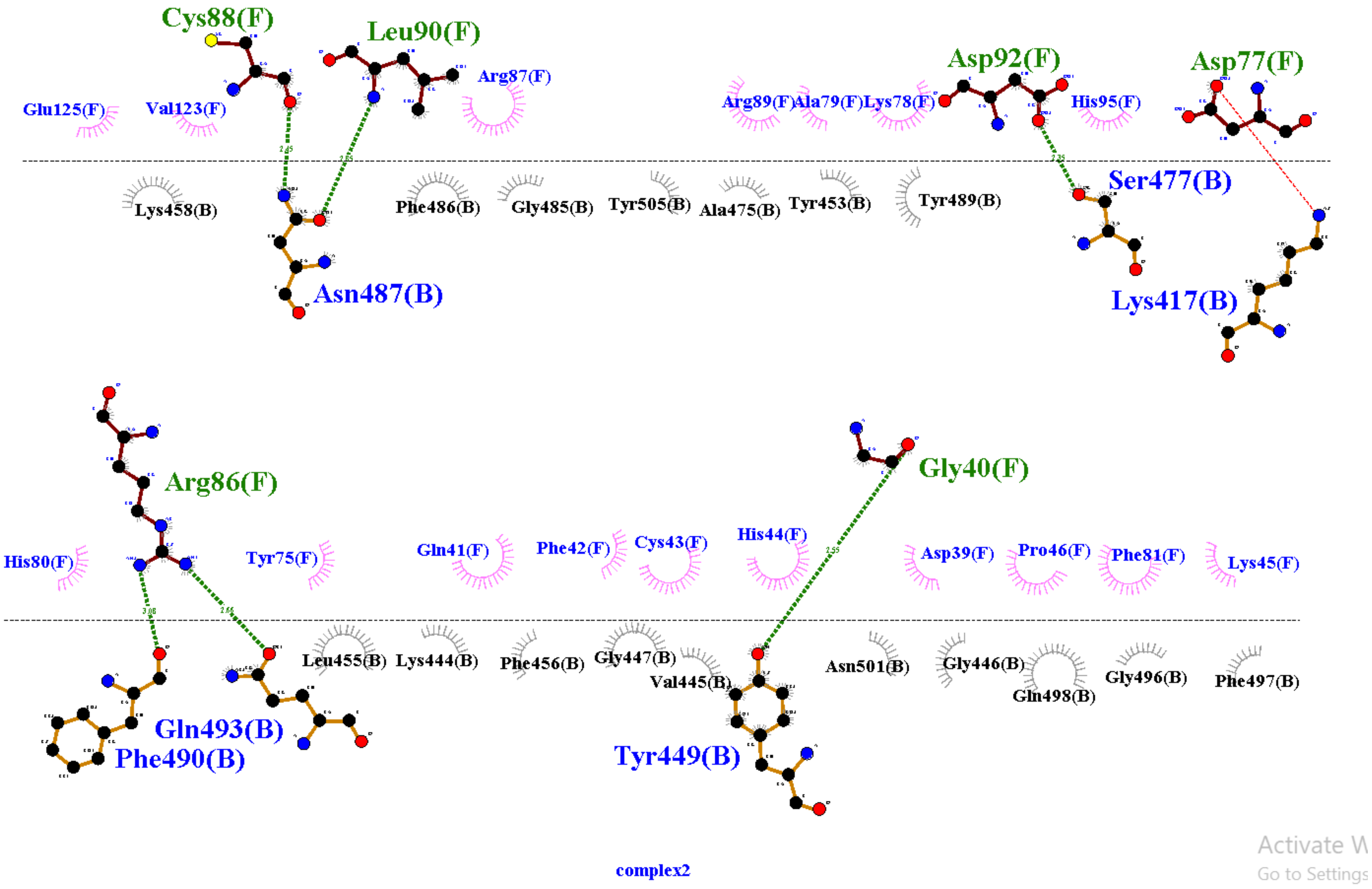

Fig S25: 2D representation of **CD95 (Chain F)** docking residues with Spike RBD (Chain B) Complex 10, provided by the LIGPLOT v.2.2 software. Olive Green lines represent hydrogen bonds, grey semi-circles denote hydrophobic interactions made Spike RBD by and Pink semi-circles represent hydrophobic interactions made by the **CD95**.

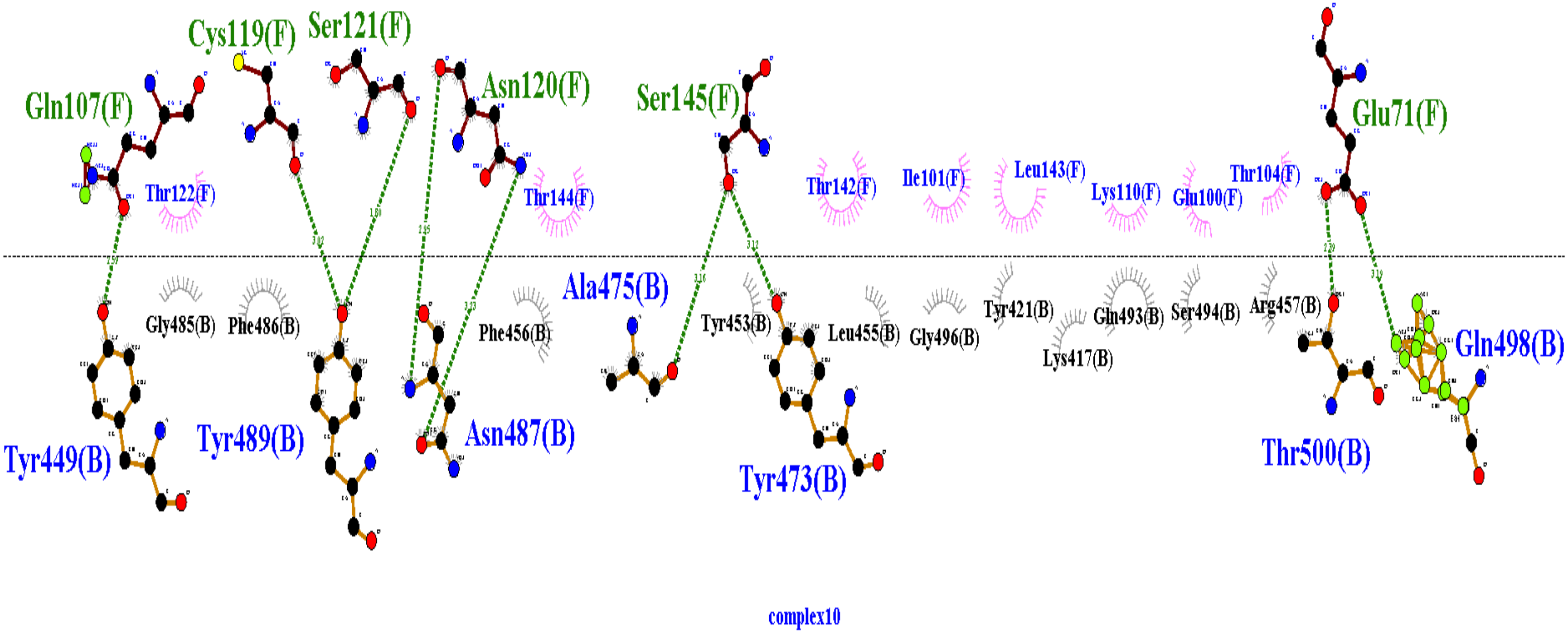

Fig S26: 2D representation of **CTLA4/CD152 (Chain A)** docking residues with Spike RBD (Chain B) Complex 2, provided by the LIGPLOT v.2.2 software. Olive Green lines represent hydrogen bonds, grey semi-circles denote hydrophobic interactions made by **CTLA4/CD152** and Pink semi-circles represent hydrophobic interactions made by the Spike RBD.

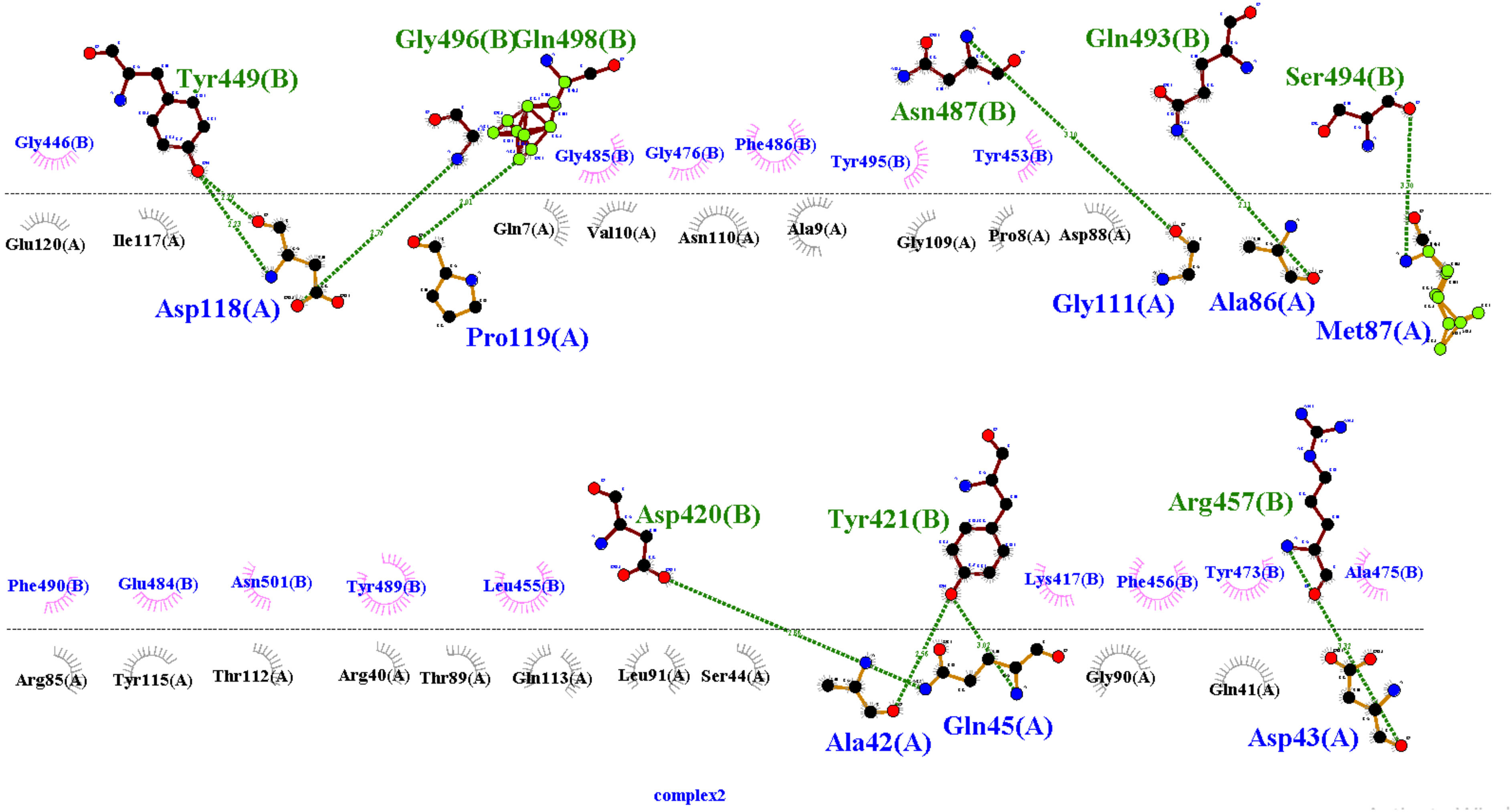

Fig S27: 2D representation of **CTLA4/CD152 (Chain A)** docking residues with Spike RBD (Chain B) Complex 3, provided by the LIGPLOT v.2.2 software. Olive Green lines represent hydrogen bonds, grey semi-circles denote hydrophobic interactions made by **CTLA4/CD152** and Pink semi-circles represent hydrophobic interactions made by the Spike RBD.

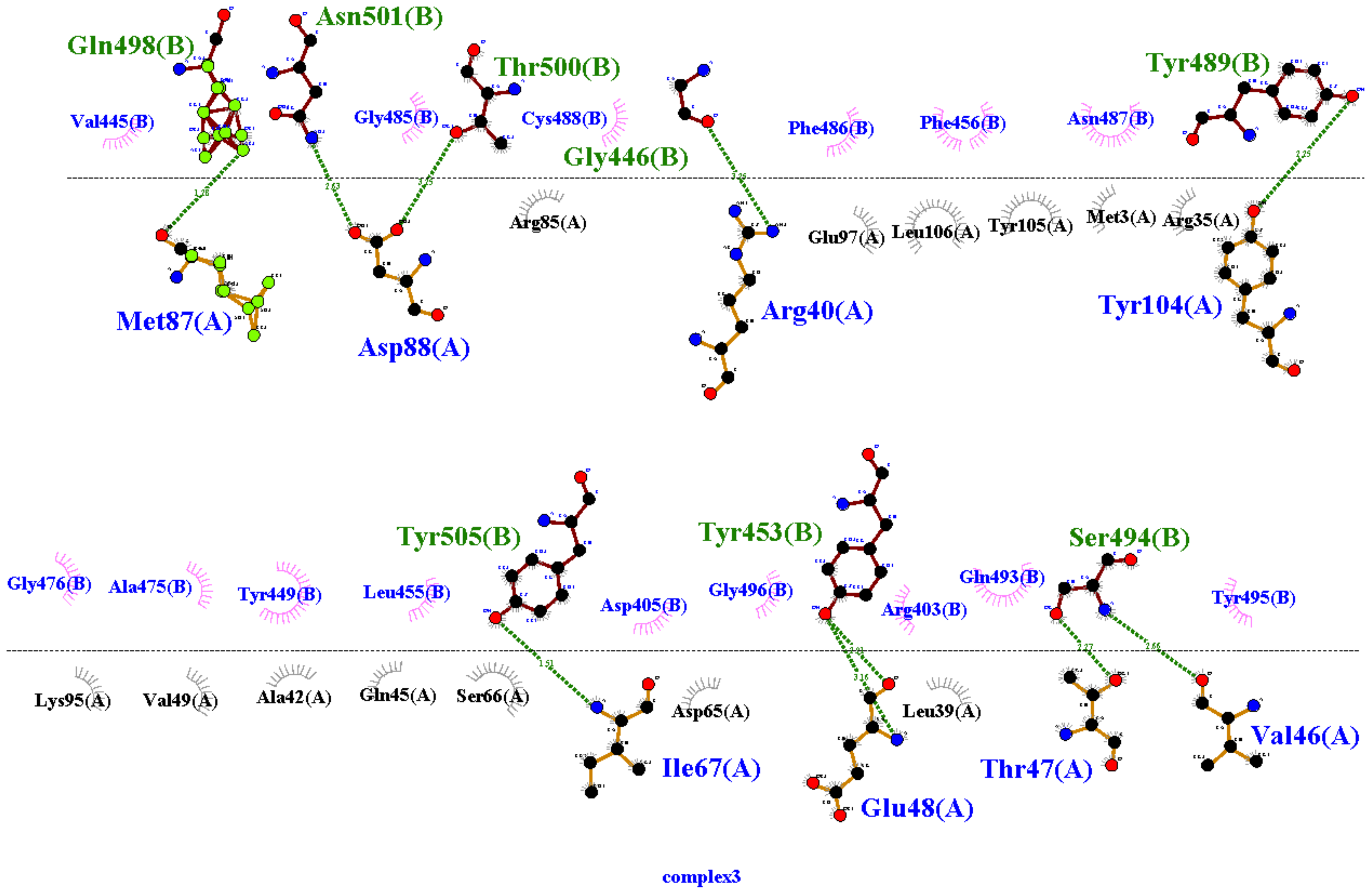

Fig S28: 2D representation of interacting residues between GCP2-GCP3 (PDB: 6V6B , Chain C: GCP2 & Chain B: GCP3), provided by the LIGPLOT v.2.2 software. Olive Green lines represent hydrogen bonds, grey semi-circles denote hydrophobic interactions made by GCP3 and Pink semi-circles represent hydrophobic interactions made by the GCP2.

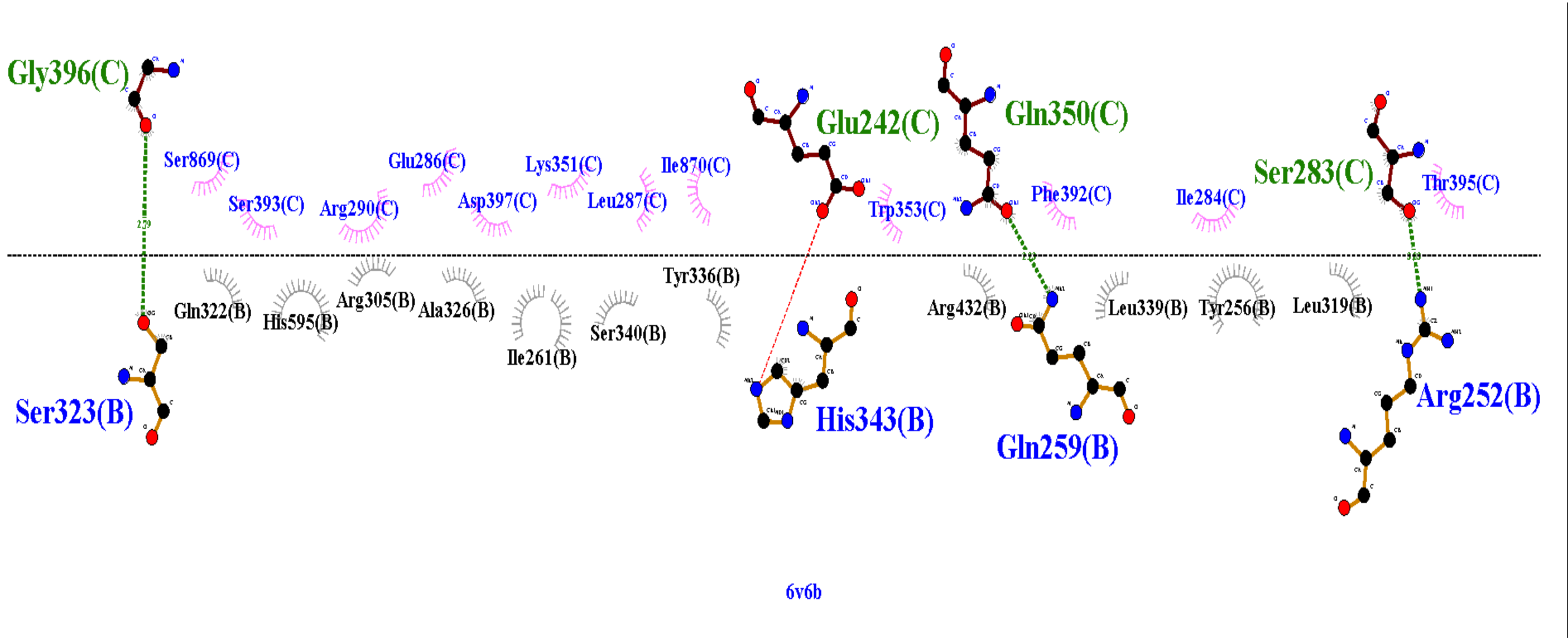

Fig S29: 2D representation of interacting residues between GCP2-TUBG1 (PDB: 6V6S , Chain C: GCP2 & Chain c: TUBG1). provided by the LIGPLOT v.2.2 software. Olive Green lines represent hydrogen bonds, grey semi-circles denote hydrophobic interactions made by GCP2 and Pink semi-circles represent hydrophobic interactions made by the TUBG1.

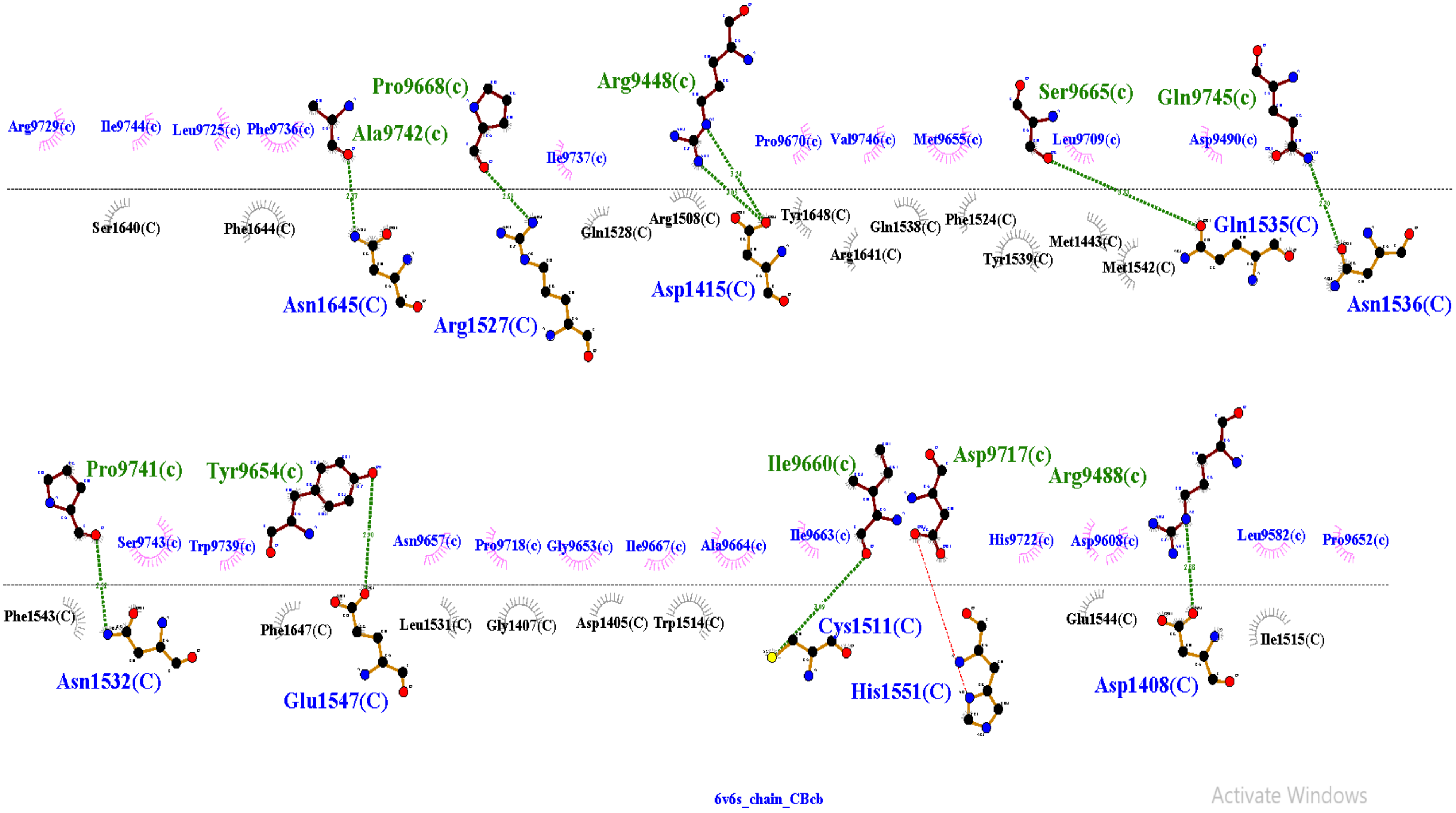

Activate Windows
Go to Settings to activate Windows

Fig S30: Representing the interacting residues between GCP2-TUBG1 (PDB: 6V6S , Chain C: GCP2 & Chain c: TUBG1) and its corresponding residues in PDB ID 6V6B (Chain C) and PDB ID 6V5V (Chain g)

**PDB ID: 6V6S GPC2 (Chain C): TUBG1 (Chain c)**	**PDB ID: 6V6B GPC2 (Chain C): PDB ID 6V5V TUBG1 (Chain g)**
Asn1645:Ala9742	Asn890:Ala354
Arg1527:Pro9668	Arg711:Pro262
Asp1415:Arg9448	Asp561:Arg3
Gln1535:Ser9665	Gln719:Ser259
Asn1536:Gln9745	Asn720:Gln357
Asn1532:Pro9741	Asn716:Pro353
Glu1547:Tyr9654	Glu731:Tyr248
Cys1511:Ile9660	Cys684:Ile254
His1551:Asp9717	His735:Asp329
Asp1408:Arg9488	Asp554:Arg47

Fig S31: 2D representation of interacting residues between GCP3-TUBG1 (PDB: 6V6S , Chain B: GCP2 & Chain b: TUBG1). provided by the LIGPLOT v.2.2 software. Olive Green lines represent hydrogen bonds, grey semi-circles denote hydrophobic interactions made by GCP3 and Pink semi-circles represent hydrophobic interactions made by the TUBG1.

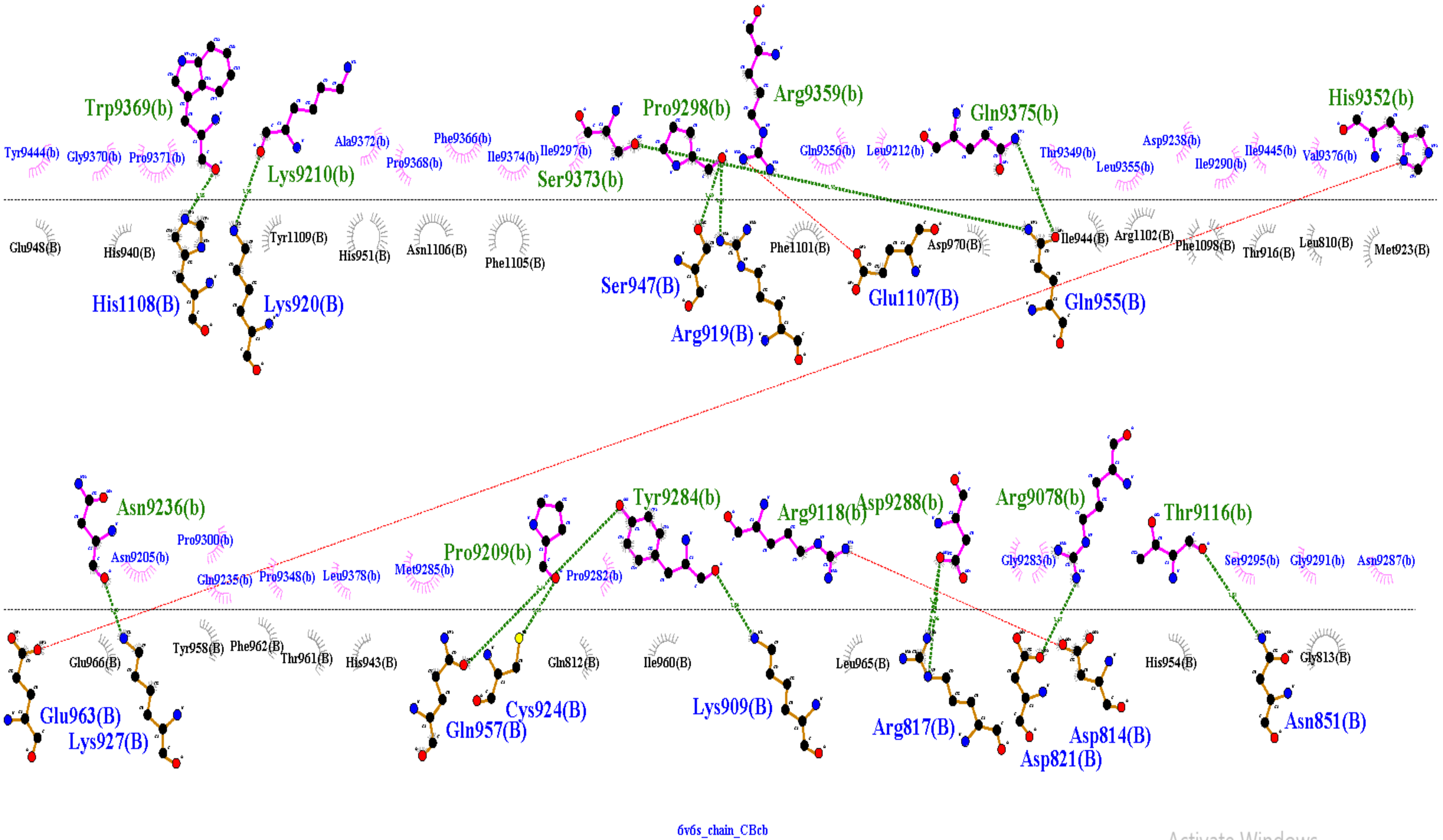

Activate Windows

Fig S32: Representing the interacting residues between GCP3-TUBG1 (PDB: 6V6S , Chain B: GCP2 & Chain b: TUBG1) and its corresponding residues in PDB ID 6V6B (Chain C) and PDB ID 6V5V (Chain g)

**PDB ID: 6V6S GPC3 (Chain B): TUBG1 (Chain b)**	**PDB ID: 6V6B GPC3 (Chain B): PDB ID 6v5v TUBG1 (Chain g)**
His1108:Trp9369	His885:Trp351
Lys920:Lys9210	Lys682:Lys163
Ser947:Pro9298	Ser709:Pro265
Arg919: Pro9298	Arg681: Pro265
Glu1107:Arg9359	Glu884:Arg341
Gln955:Gln9375	Gln717:Gln357
Gln955:Ser9373	Gln717:Ser355
Glu963:His9352	Glu725:His334
Lys927:Asn9236	Lys689:Asn198
Cys924:Pro9209	Cys686:Pro162
Gln957:Tyr9284	Gln719:Tyr248
Lys909: Tyr9284	Lys671: Tyr248
Asp814:Arg9118	Asp572:Arg47
Arg817:Asp9288	Arg575:Asp252
Asp821:Arg9078	Asp579:Arg3
Asn851:Thr9116	Asn609:Thr45

Fig S33: 2D representation of interacting residues between GCP2 (Chain C) and CoV-2 M Protein (Chain A), Complex 7, provided by the LIGPLOT v.2.2 software. Olive Green lines represent hydrogen bonds, grey semi-circles denote hydrophobic interactions made by GCP2 and Pink semi-circles represent hydrophobic interactions made by the CoV-2 M Protein .

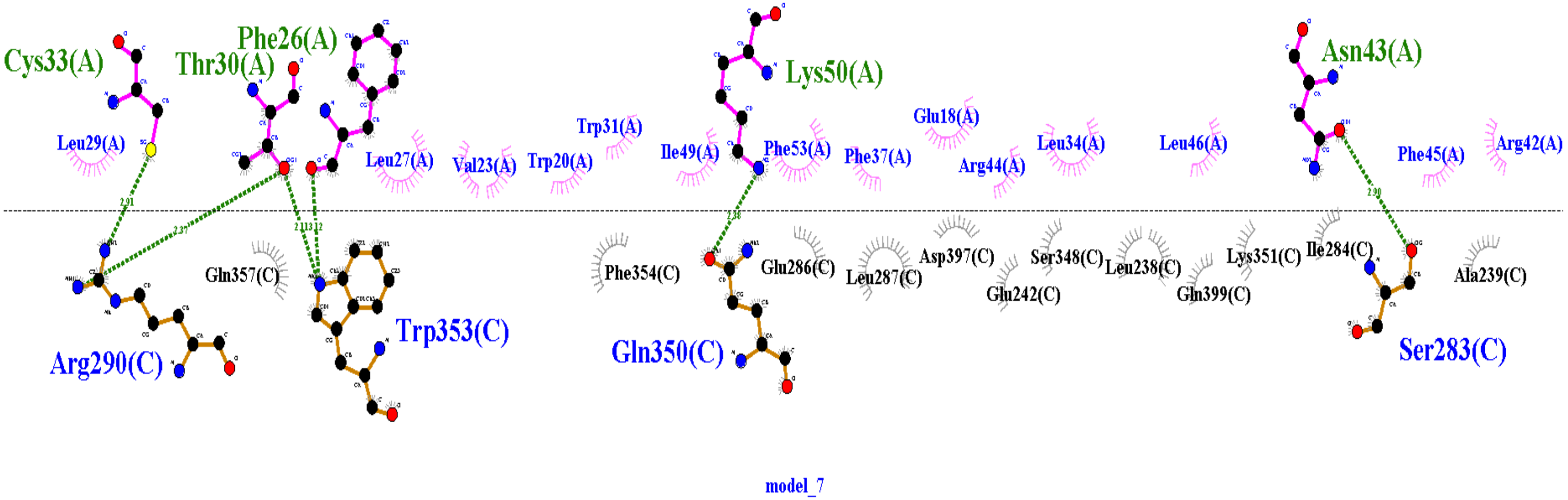

Fig S34: 2D representation of interacting residues between GCP2 (Chain C) and CoV-2 M Protein (Chain A), Complex 3, provided by the LIGPLOT v.2.2 software. Olive Green lines represent hydrogen bonds, grey semi-circles denote hydrophobic interactions made by GCP2 and Pink semi-circles represent hydrophobic interactions made by the CoV-2 M Protein .

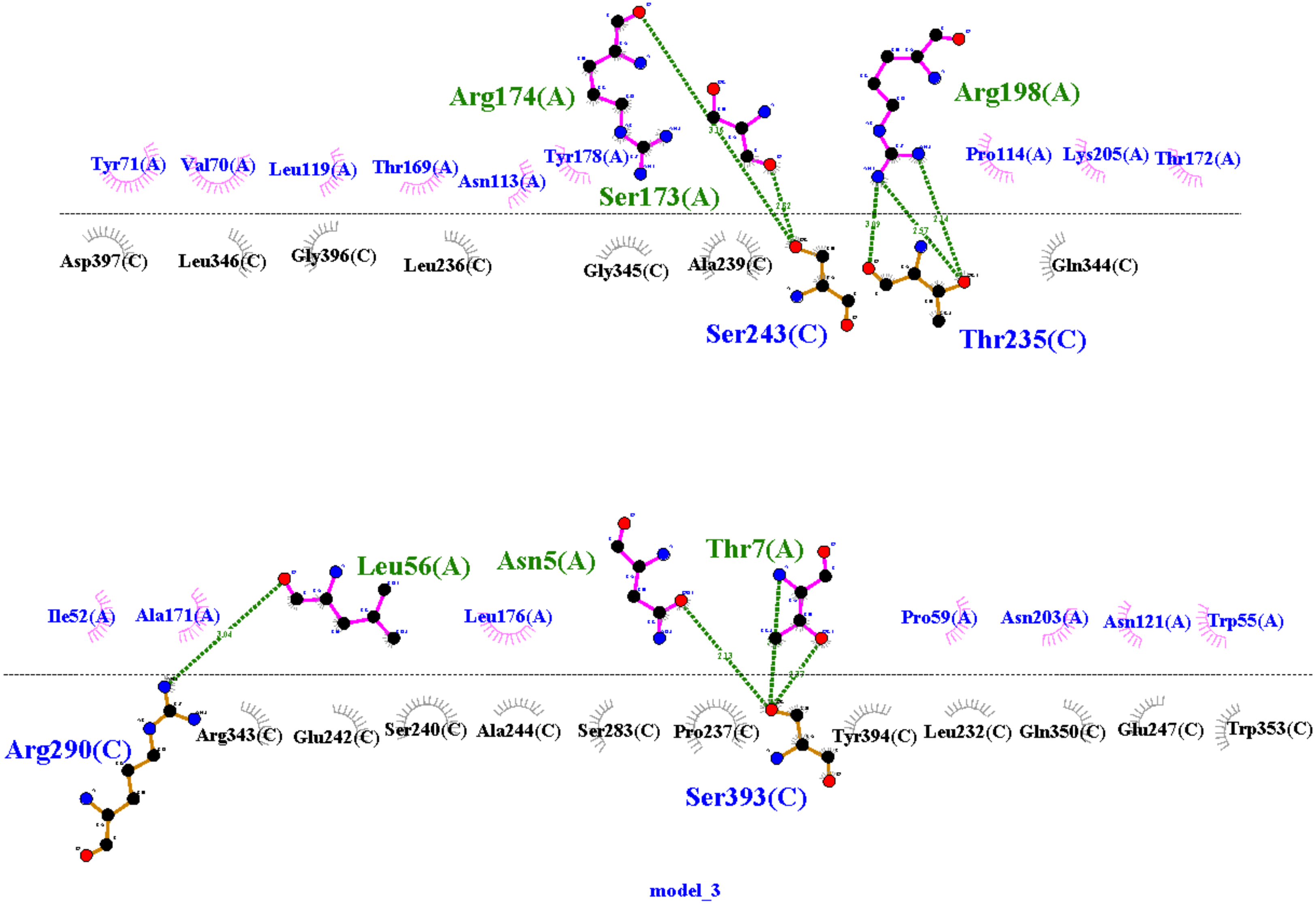

Fig S35: 2D representation of interacting residues between GCP2 (Chain C) and Orf3a (Chain A), Complex 4, provided by the LIGPLOT v.2.2 software. Olive Green lines represent hydrogen bonds, grey semi-circles denote hydrophobic interactions made by GCP2 and Pink semi-circles represent hydrophobic interactions made by the Orf3a .

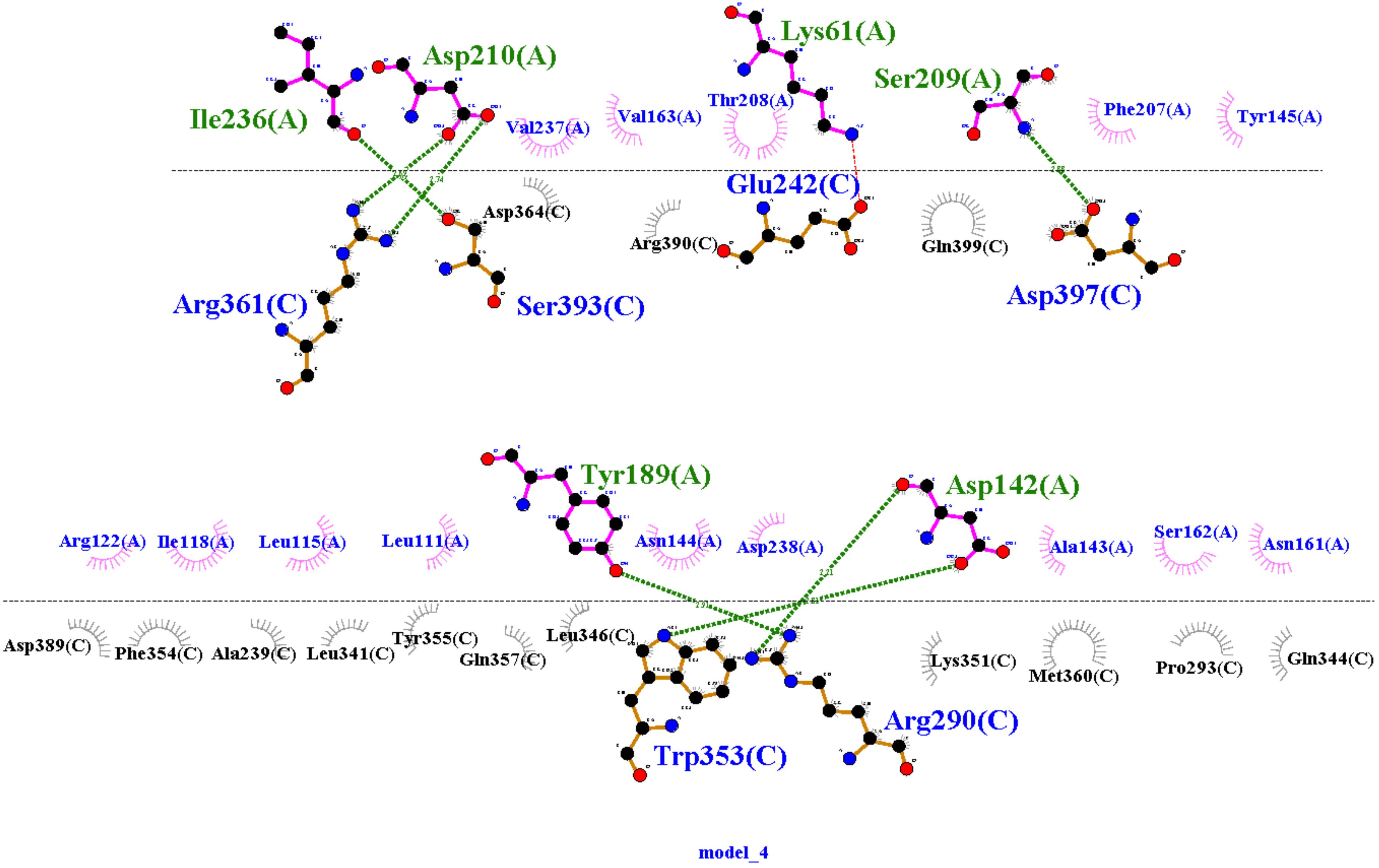

Fig S36: 2D representation of interacting residues between GCP3 (Chain B) and Orf3a (Chain A), Complex 7, provided by the LIGPLOT v.2.2 software. Olive Green lines represent hydrogen bonds, grey semi-circles denote hydrophobic interactions made by GCP3 and Pink semi-circles represent hydrophobic interactions made by the Orf3a .

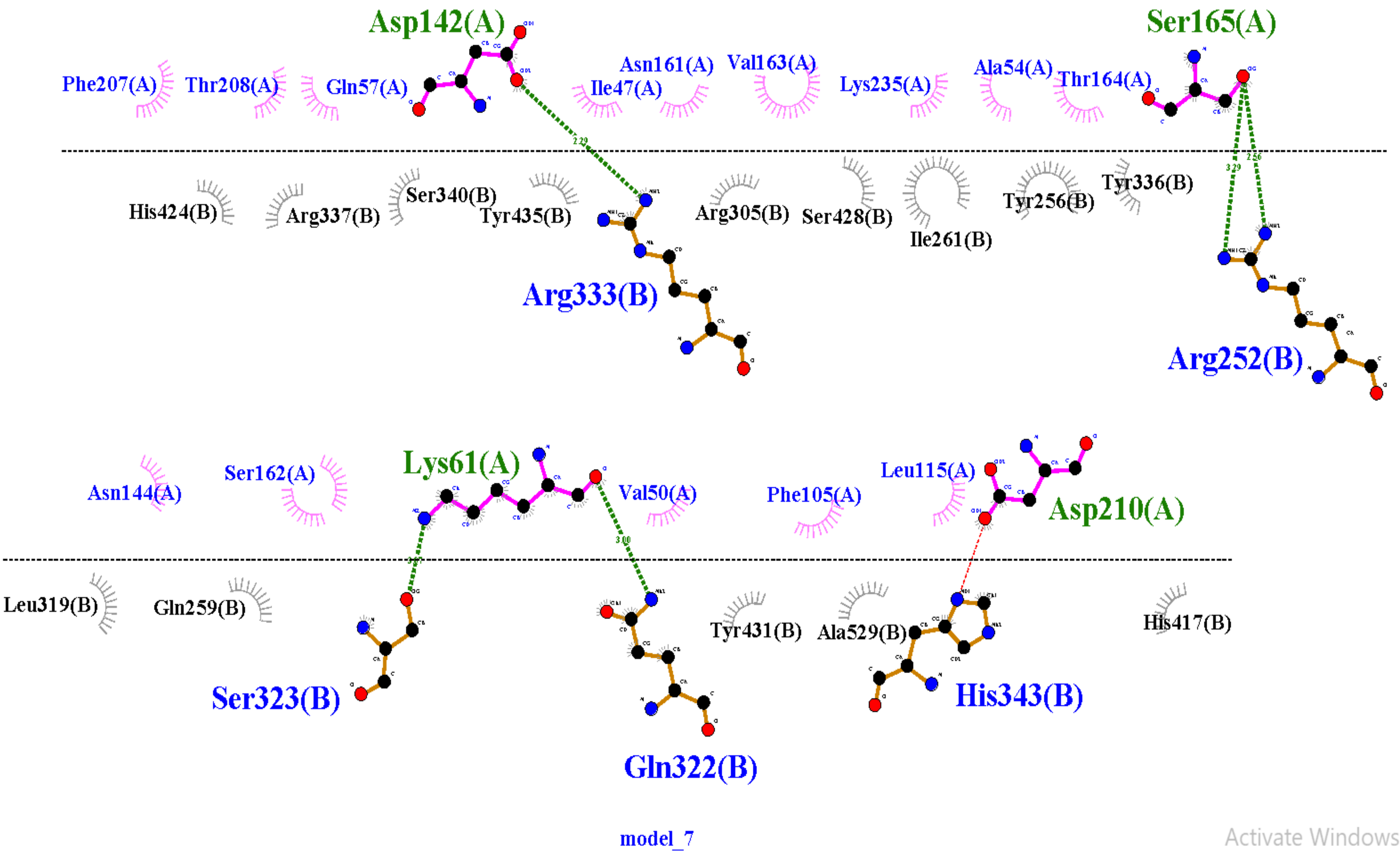

Activate Windows

Fig S37: 2D representation of interacting residues between GCP3 (Chain B) and Orf3a (Chain A), Complex 3, provided by the LIGPLOT v.2.2 software. Olive Green lines represent hydrogen bonds, grey semi-circles denote hydrophobic interactions made by GCP3 and Pink semi-circles represent hydrophobic interactions made by the Orf3a .

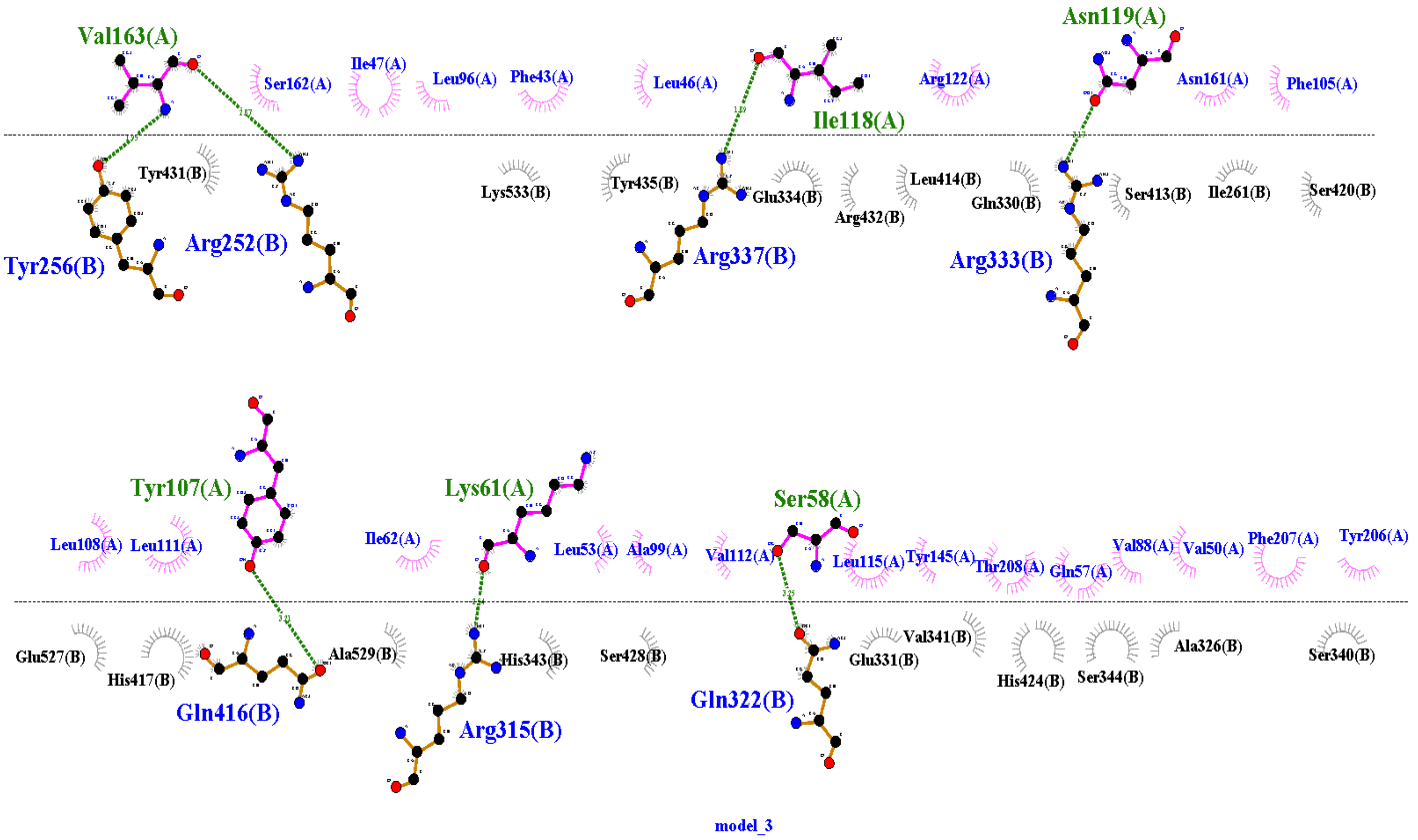

Fig S38: 2D representation of interacting residues between GCP2 (At GCP2 residues that interact with TUBG1) (Chain C) and M Protein (Chain A), Complex 5, provided by the LIGPLOT v.2.2 software. Olive Green lines represent hydrogen bonds, grey semi-circles denote hydrophobic interactions made by GCP2 and Pink semi-circles represent hydrophobic interactions made by the M Protein.

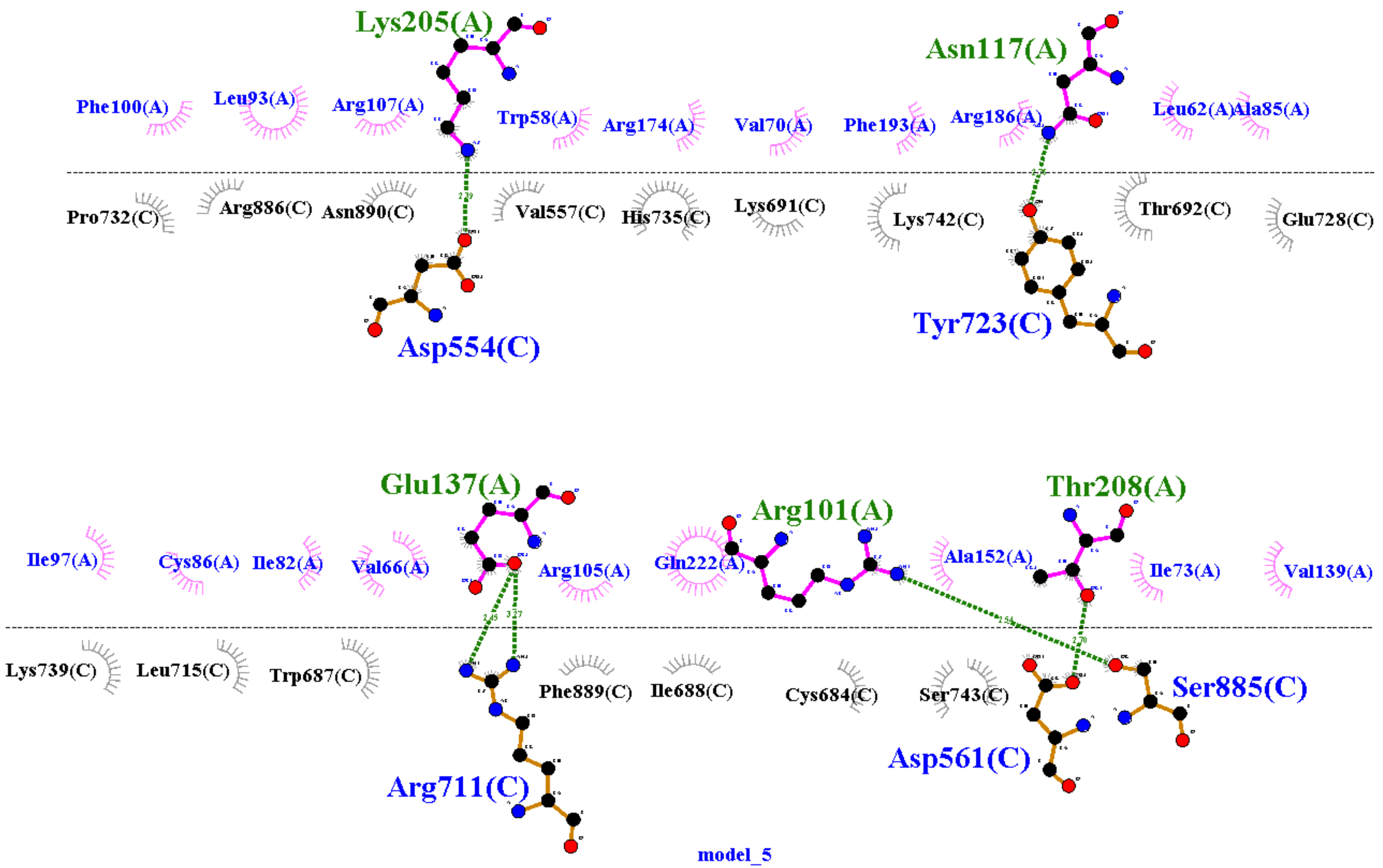

Fig S39: 2D representation of interacting residues between GCP2 (At GCP2 residues that interact with TUBG1) (Chain C) and M Protein (Chain A), Complex 1 , provided by the LIGPLOT v.2.2 software. Olive Green lines represent hydrogen bonds, grey semi-circles denote hydrophobic interactions made by GCP2 and Pink semi-circles represent hydrophobic interactions made by the M Protein.

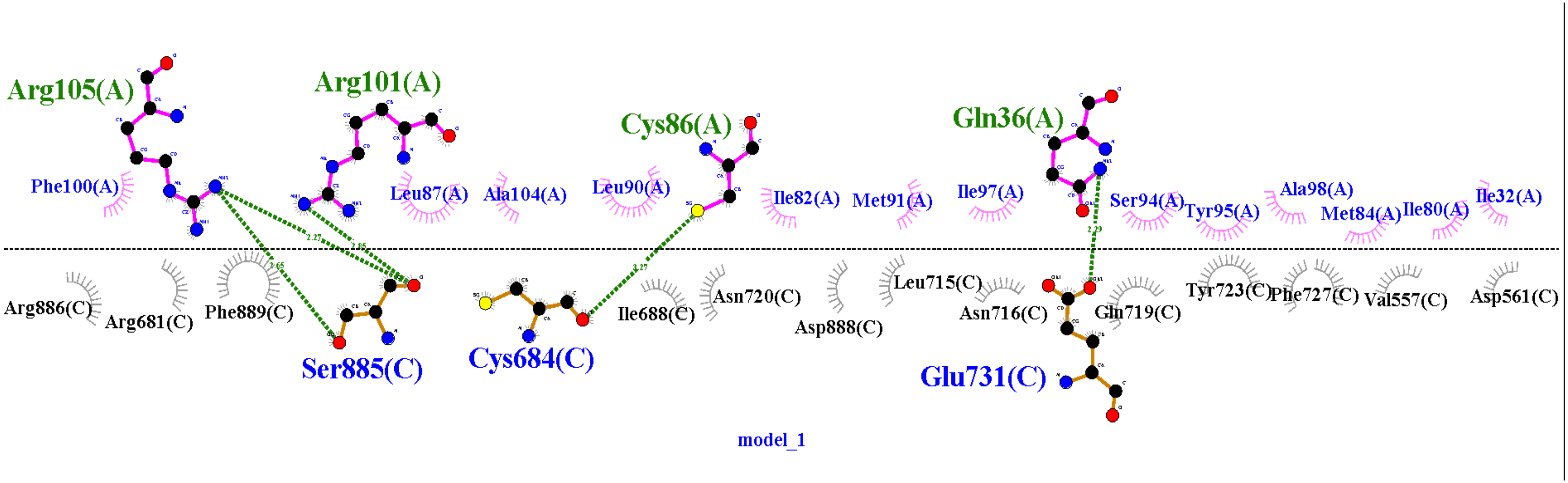

Fig S40: 2D representation of interacting residues between GCP3 (AtGCP3 residues that interact with TUBG1) (Chain B) and M Protein (Chain A), Complex 1, , provided by the LIGPLOT v.2.2 software. Olive Green lines represent hydrogen bonds, grey semi-circles denote hydrophobic interactions made by GCP3 and Pink semi-circles represent hydrophobic interactions made by the M Protein.

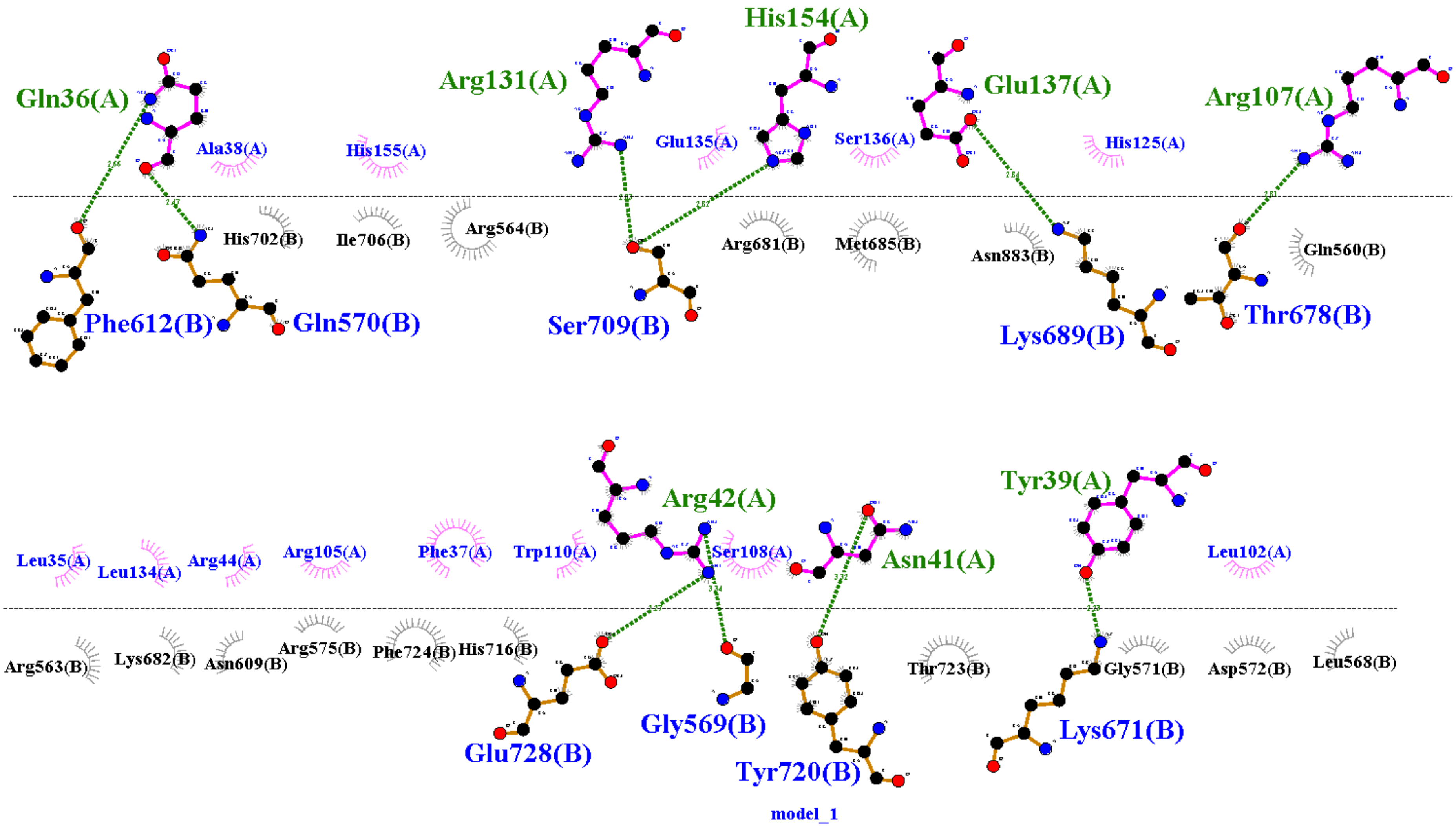

Fig S41: 2D representation of interacting residues between GCP3 (AtGCP3 residues that interact with TUBG1) (Chain B) and M Protein (Chain A), Complex 5, provided by the LIGPLOT v.2.2 software. Olive Green lines represent hydrogen bonds, grey semi-circles denote hydrophobic interactions made by GCP3 and Pink semi-circles represent hydrophobic interactions made by the M Protein.

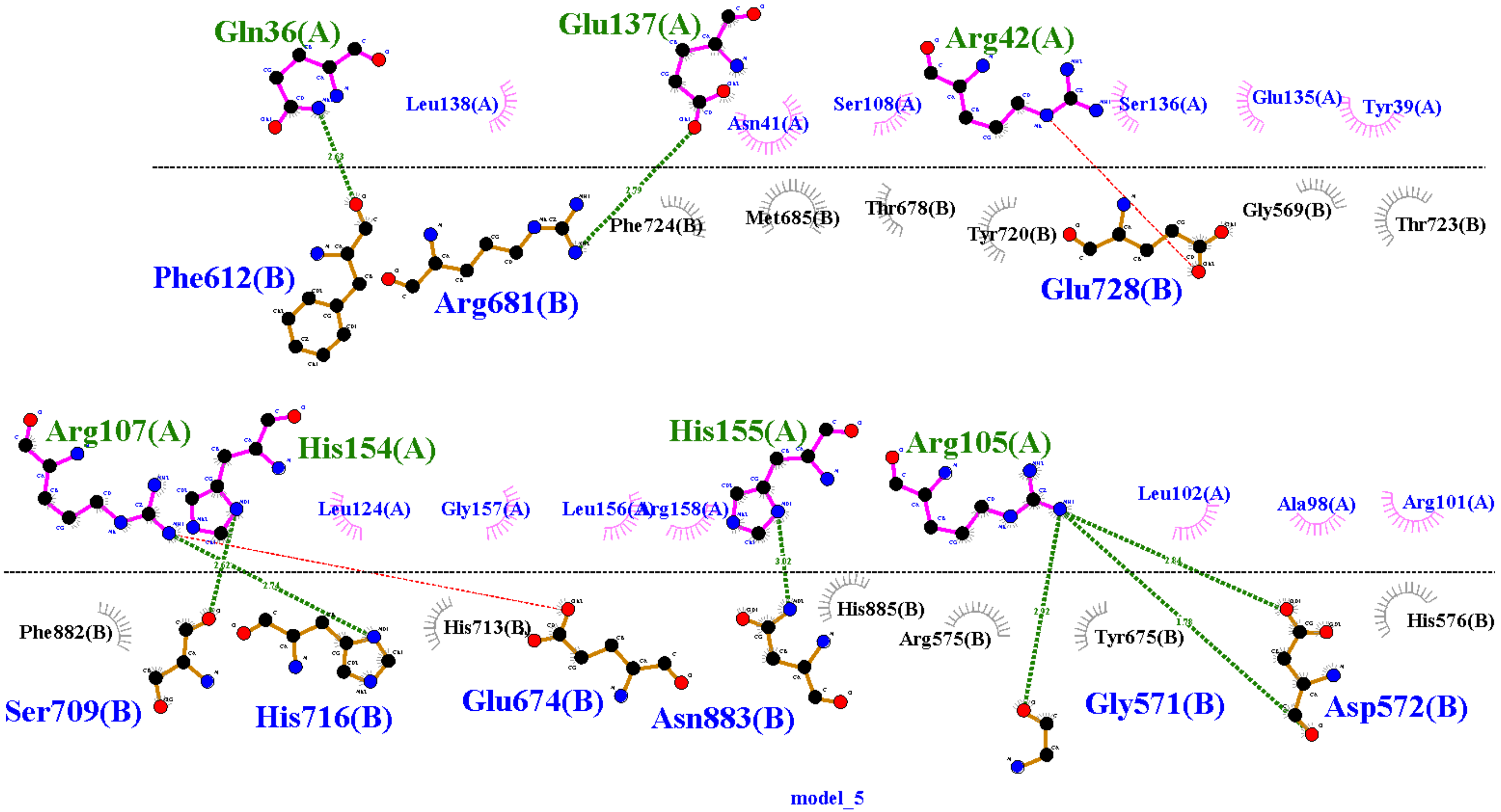

Fig S42: 2D representation of interacting residues between GCP2 (At GCP2 residues that interact with TUBG1) (Chain C) and Orf3a (Chain A), Complex 3, provided by the LIGPLOT v.2.2 software. Olive Green lines represent hydrogen bonds, grey semi-circles denote hydrophobic interactions made by GCP2 and Pink semi-circles represent hydrophobic interactions made by the Orf3a.

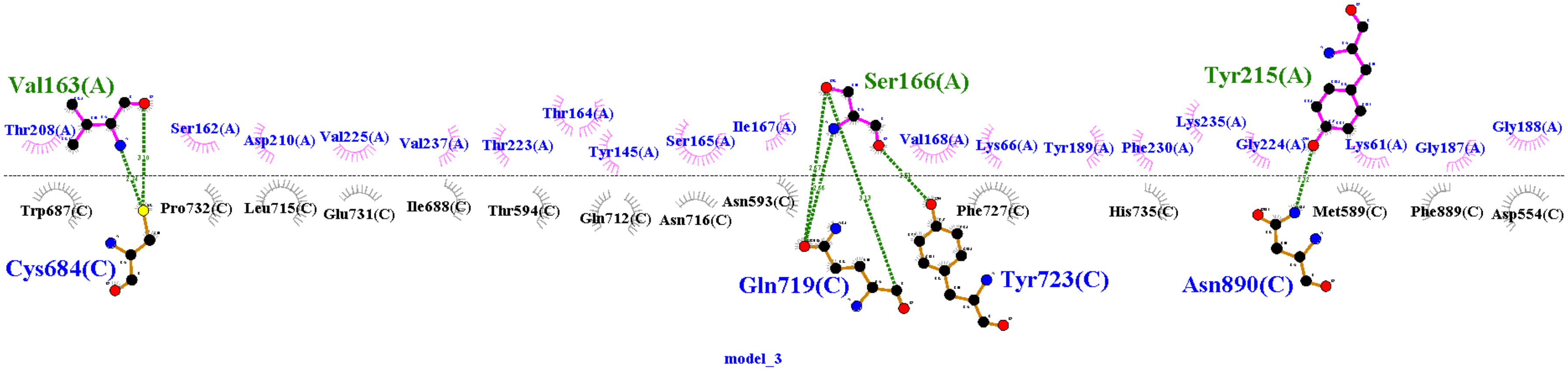

Fig S43: 2D representation of interacting residues between GCP2 (At GCP2 residues that interact with TUBG1) (Chain C) and Orf3a (Chain A), Complex 9, provided by the LIGPLOT v.2.2 software. Olive Green lines represent hydrogen bonds, grey semi-circles denote hydrophobic interactions made by GCP2 and Pink semi-circles represent hydrophobic interactions made by the Orf3a.

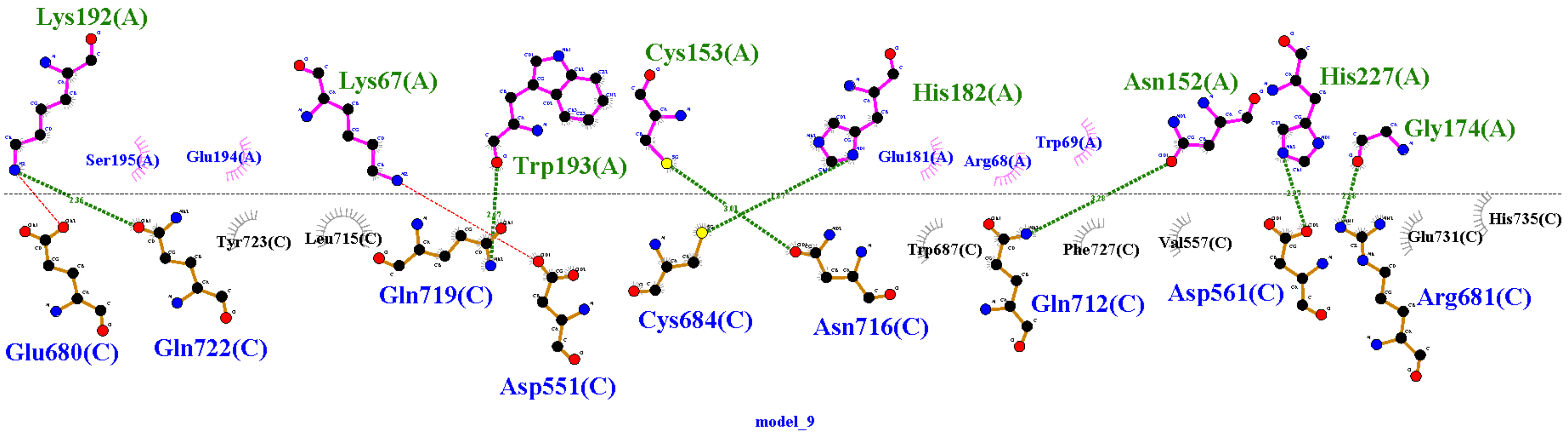

Fig S44: 2D representation of interacting residues between GCP3 (At GCP3 residues that interact with TUBG1) (Chain B) and Orf3a (Chain A), Complex 1, provided by the LIGPLOT v.2.2 software. Olive Green lines represent hydrogen bonds, grey semi-circles denote hydrophobic interactions made by GCP3 and Pink semi-circles represent hydrophobic interactions made by the Orf3a.

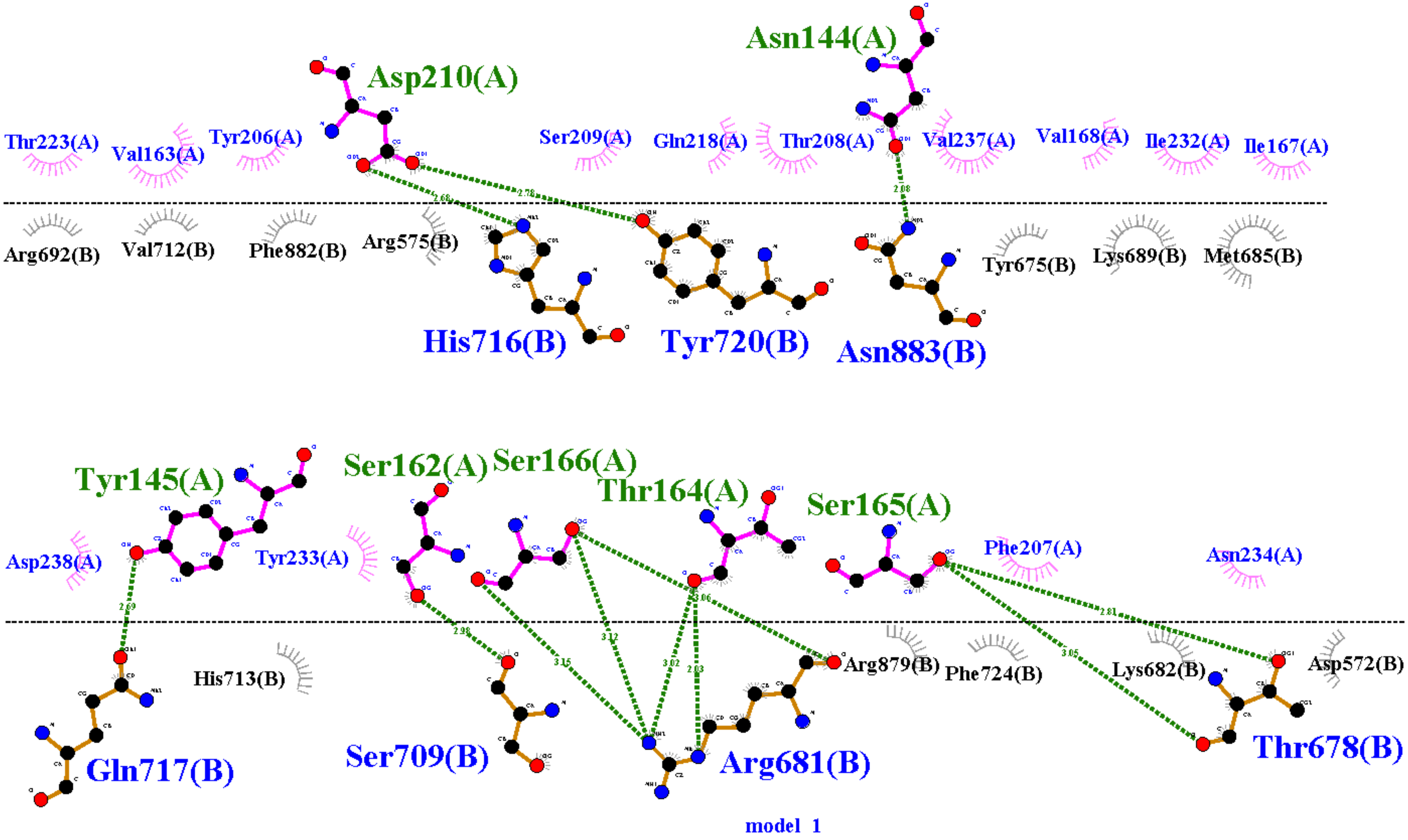

Fig S45: 2D representation of interacting residues between GCP3 (At GCP3 residues that interact with TUBG1) (Chain B) and Orf3a (Chain A), Complex 3. provided by the LIGPLOT v.2.2 software. Olive Green lines represent hydrogen bonds, grey semi-circles denote hydrophobic interactions made by GCP3 and Pink semi-circles represent hydrophobic interactions made by the Orf3a.

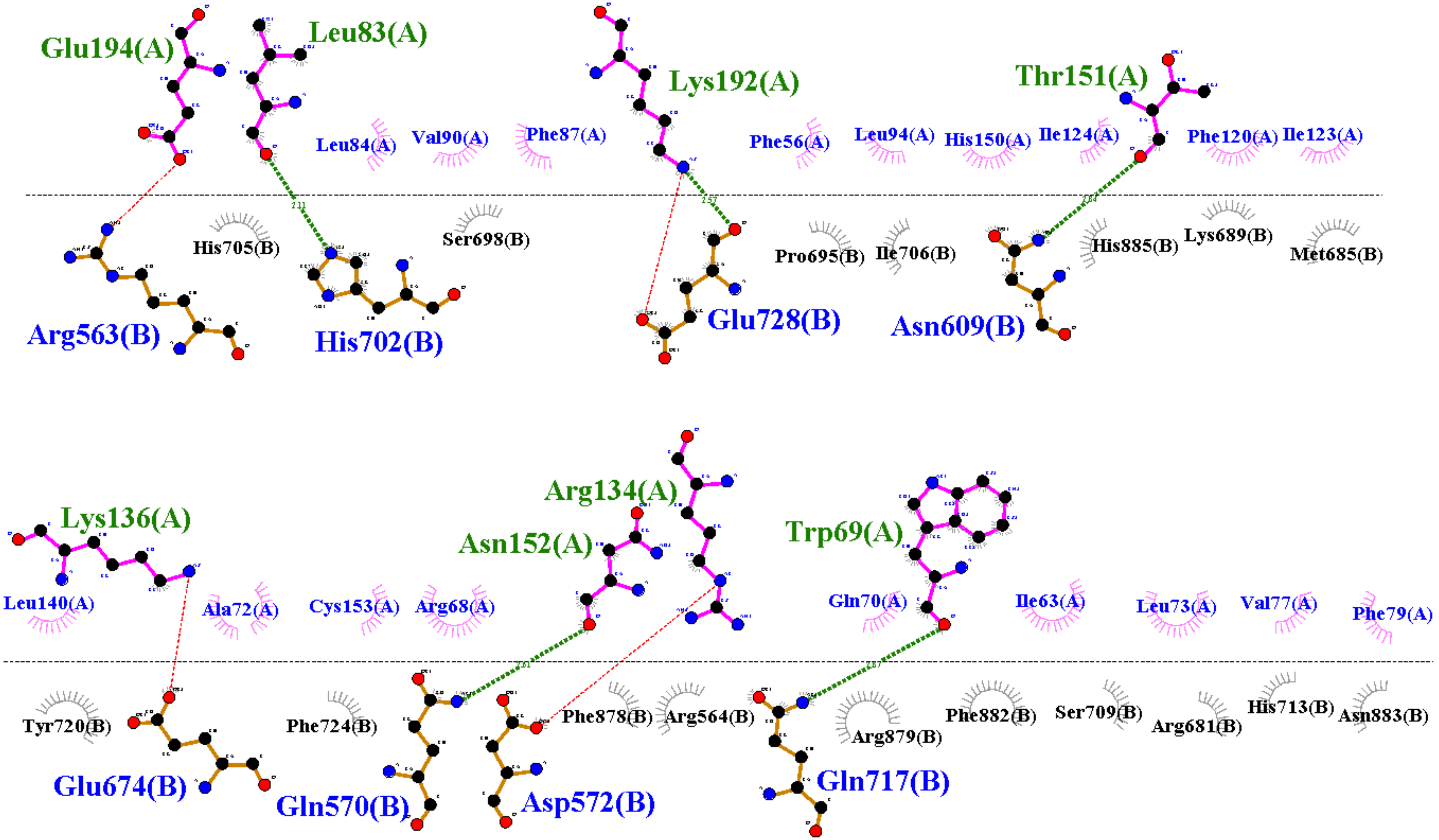

Fig S46: 2D representation of interacting residues between TUBG1 (At TUBG1 residues that interact with GCP2) (Chain g) and M Protein (Chain A), Complex 1, provided by the LIGPLOT v.2.2 software. Olive Green lines represent hydrogen bonds, grey semi-circles denote hydrophobic interactions made by TUBG1 and Pink semi-circles represent hydrophobic interactions made by the M Protein.

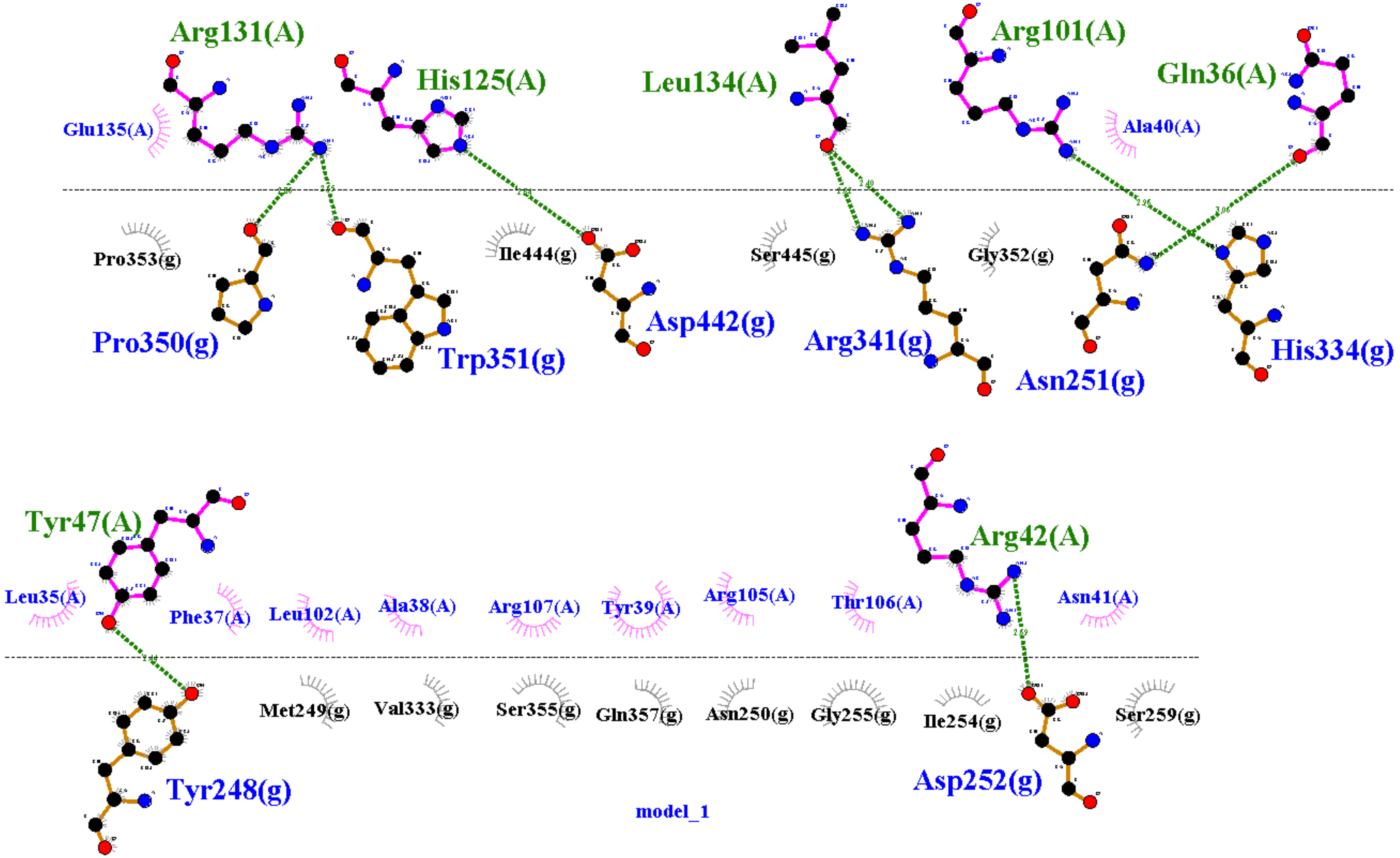

Fig S47: 2D representation of interacting residues between TUBG1 (At TUBG1 residues that interact with GCP2) (Chain g) and M Protein (Chain A), Complex 7, provided by the LIGPLOT v.2.2 software. Olive Green lines represent hydrogen bonds, grey semi-circles denote hydrophobic interactions made by TUBG1 and Pink semi-circles represent hydrophobic interactions made by the M Protein.

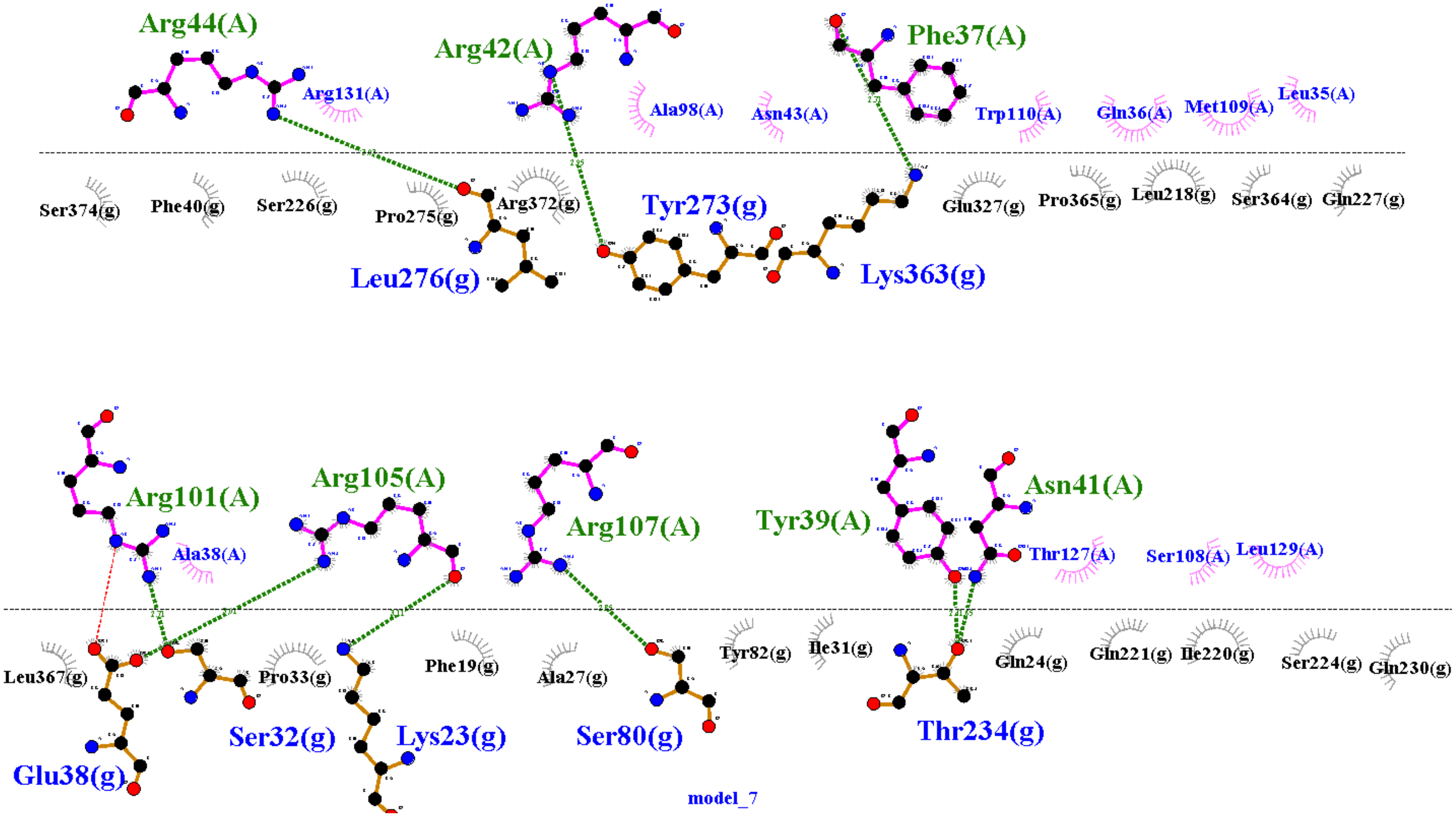

Fig S48: 2D representation of interacting residues between TUBG1 (At TUBG1 residues that interact with GCP2) (Chain g) and M Protein (Chain A), Complex 8, provided by the LIGPLOT v.2.2 software. Olive Green lines represent hydrogen bonds, grey semi-circles denote hydrophobic interactions made by TUBG1 and Pink semi-circles represent hydrophobic interactions made by the M Protein.

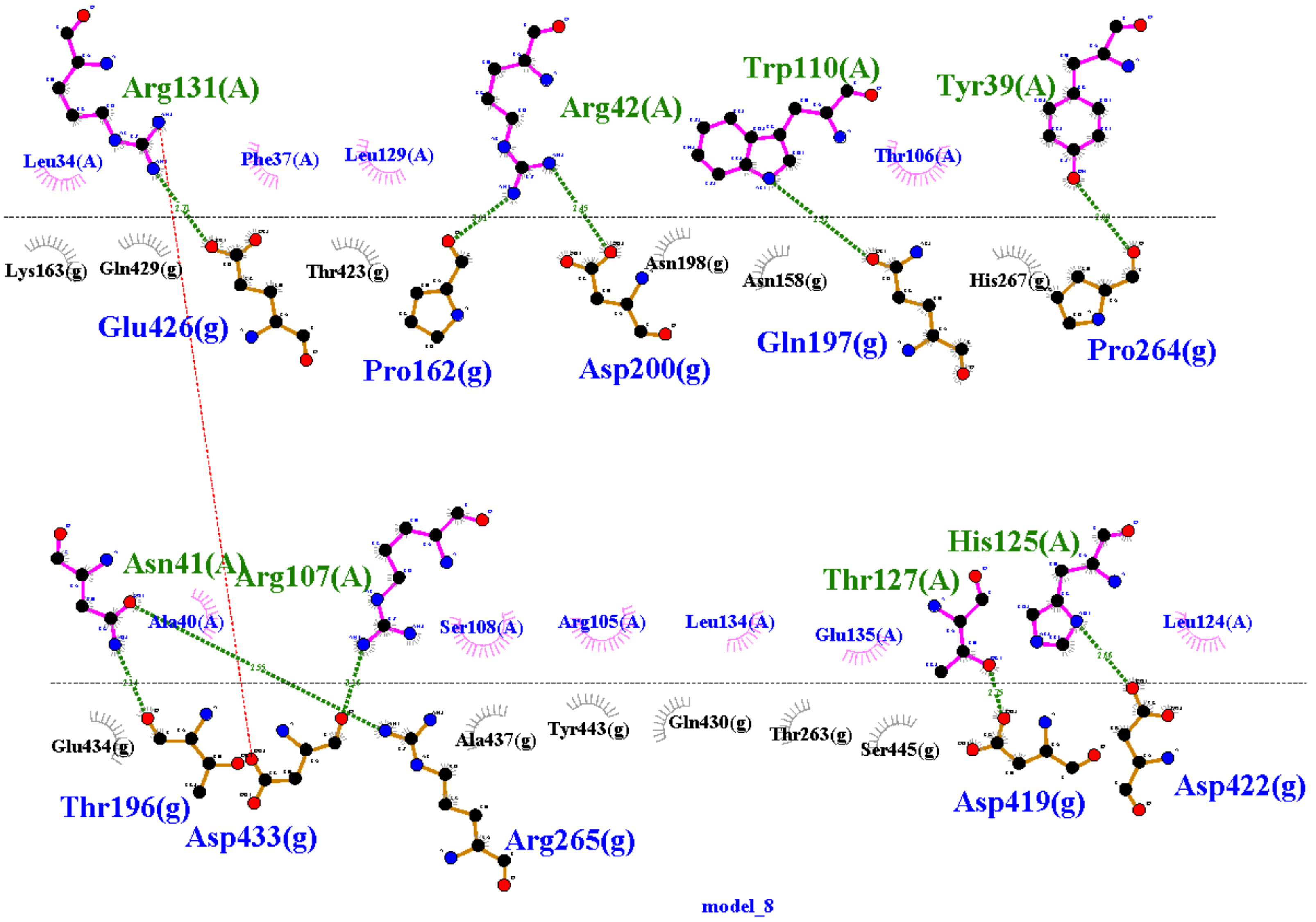

Fig S49: 2D representation of interacting residues between TUBG1 (At TUBG1 residues that interact with GCP3) (Chain g) and M Protein (Chain A), Complex 1, provided by the LIGPLOT v.2.2 software. Olive Green lines represent hydrogen bonds, grey semi-circles denote hydrophobic interactions made by TUBG1 and Pink semi-circles represent hydrophobic interactions made by the M Protein.

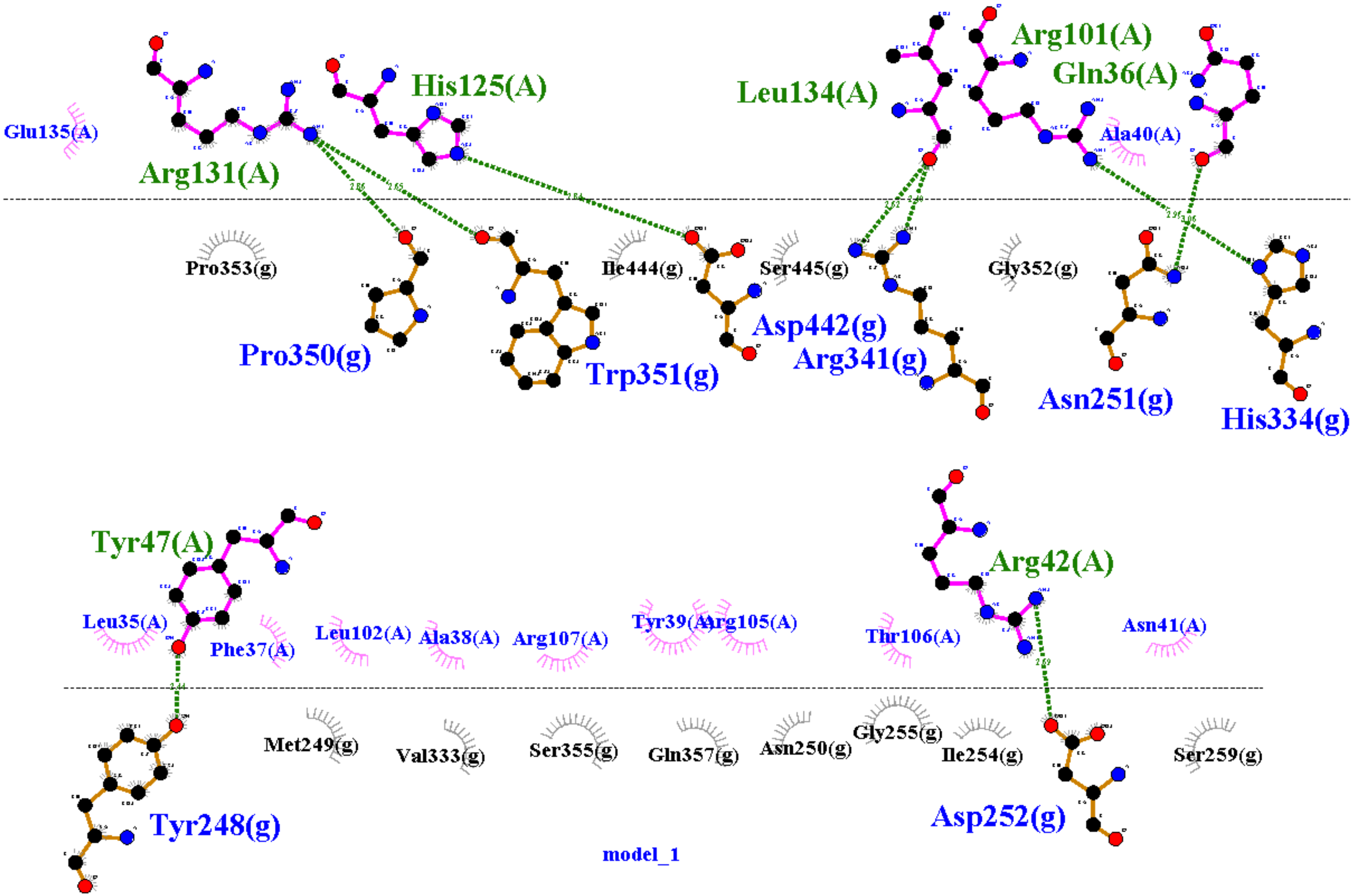

Fig S50: 2D representation of interacting residues between TUBG1 (At TUBG1 residues that interact with GCP3) (Chain g) and M Protein (Chain A), Complex 8, provided by the LIGPLOT v.2.2 software. Olive Green lines represent hydrogen bonds, grey semi-circles denote hydrophobic interactions made by TUBG1 and Pink semi-circles represent hydrophobic interactions made by the M Protein.

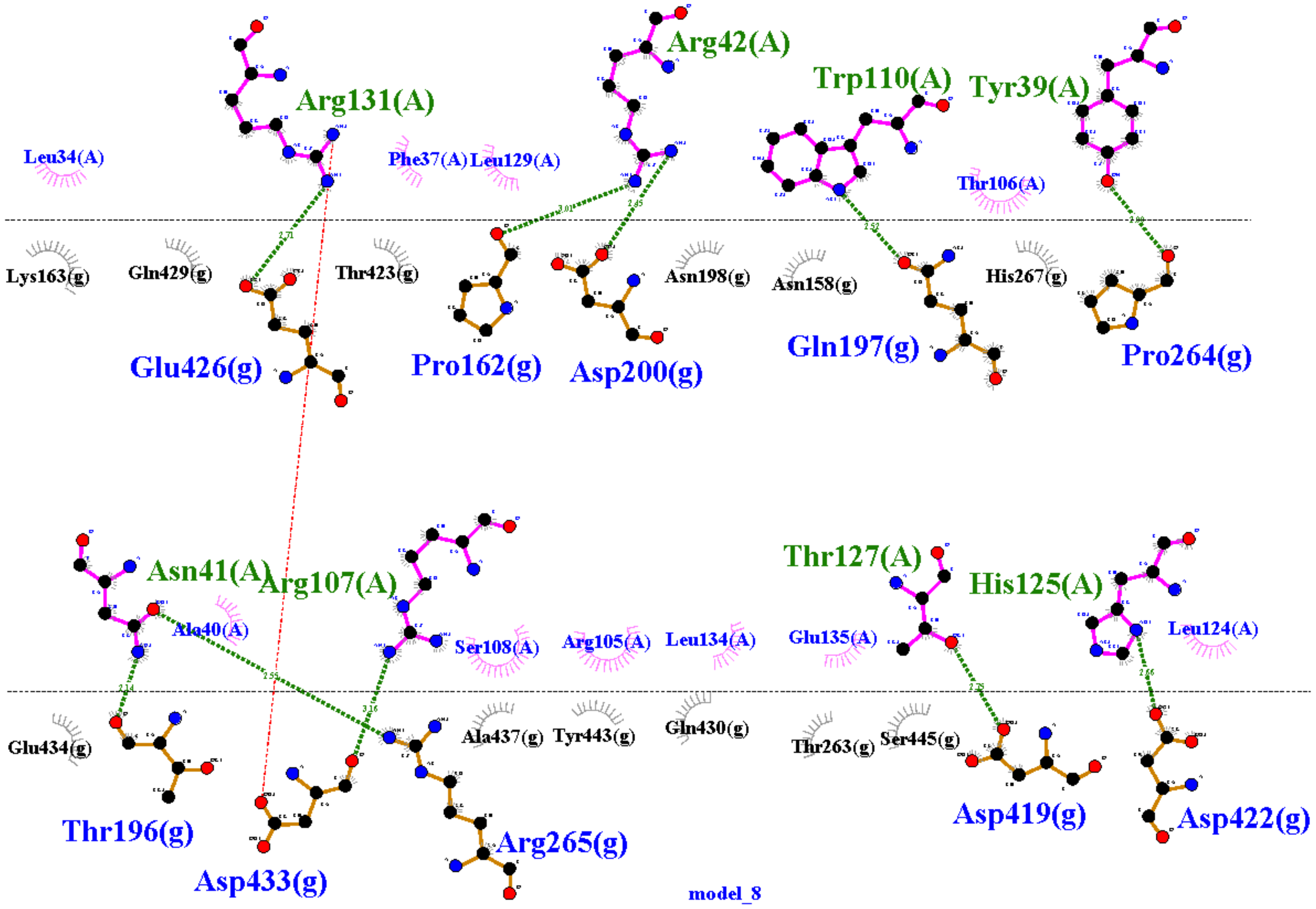

Fig S51: 2D representation of interacting residues between TUBG1 (At TUBG1 residues that interact with GCP3) (Chain g) and M Protein (Chain A), Complex 9,provided by the LIGPLOT v.2.2 software. Olive Green lines represent hydrogen bonds, grey semi-circles denote hydrophobic interactions made by TUBG1 and Pink semi-circles represent hydrophobic interactions made by the M Protein.

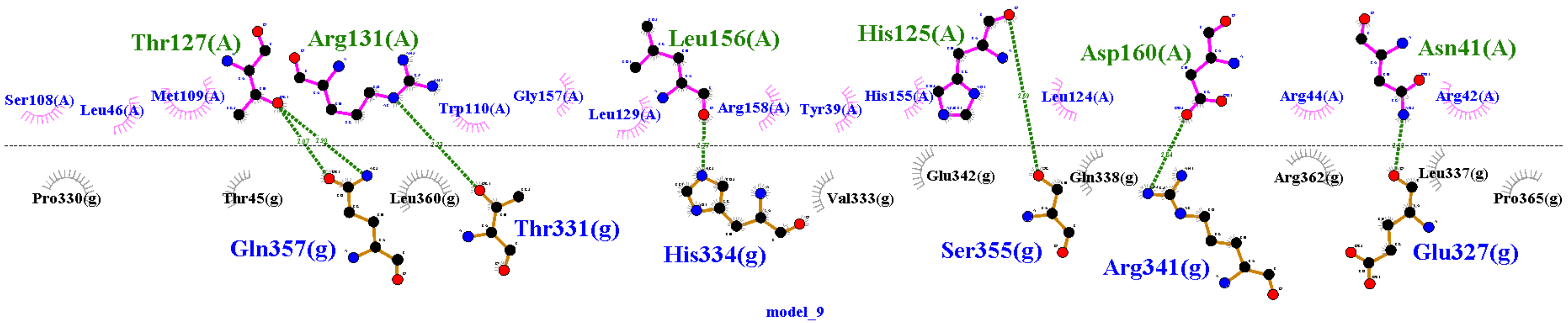

Fig S52: 2D representation of interacting residues between TUBG1 (At TUBG1 residues that interact with GCP2) (Chain g) and orf3a (Chain A), Complex 3 provided by the LIGPLOT v.2.2 software. Olive Green lines represent hydrogen bonds, grey semi-circles denote hydrophobic interactions made by TUBG1 and Pink semi-circles represent hydrophobic interactions made by the orf3a.

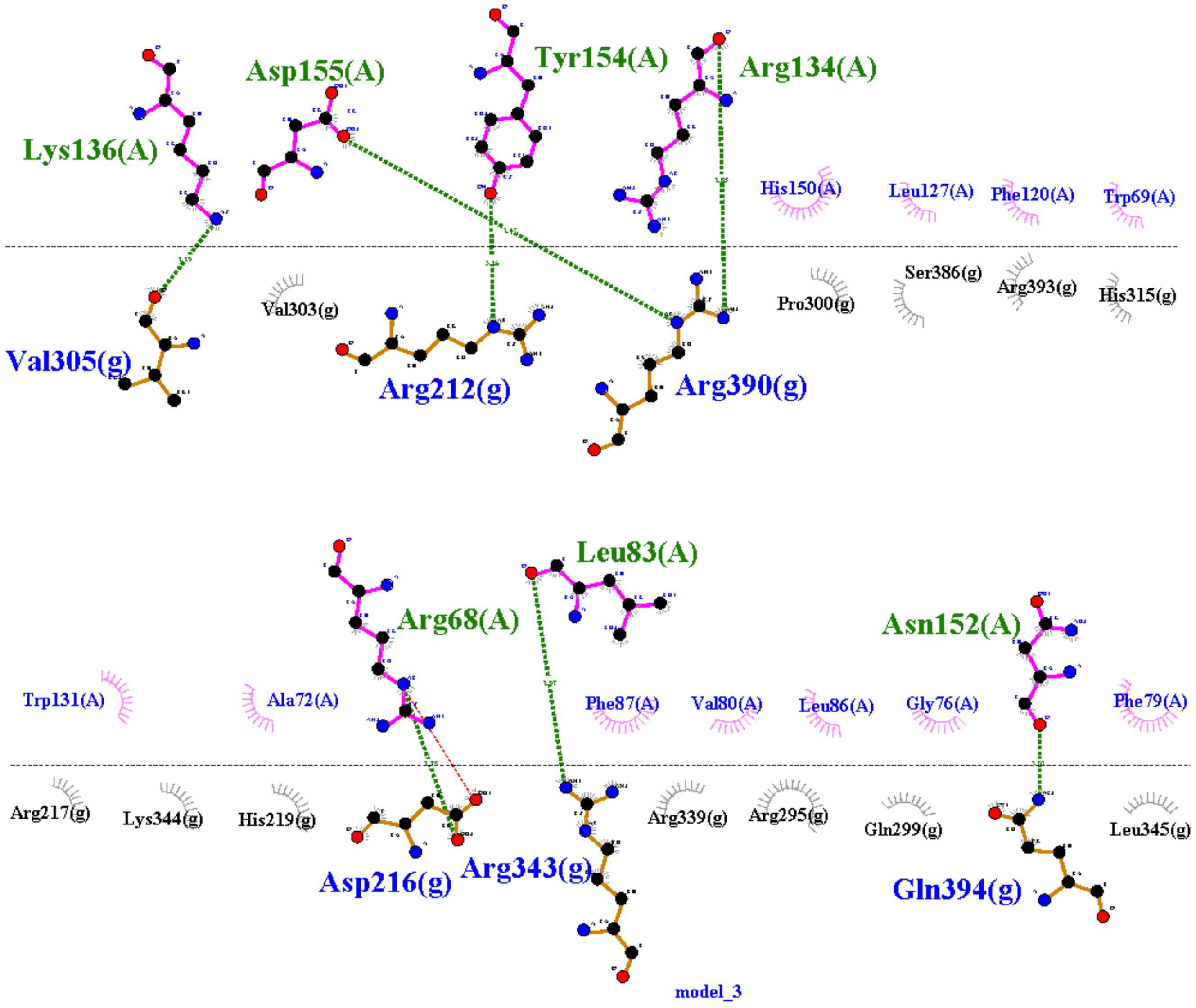

Fig S53: 2D representation of interacting residues between TUBG1 (At TUBG1 residues that interact with GCP2) (Chain g) and orf3a (Chain A), Complex 1,provided by the LIGPLOT v.2.2 software. Olive Green lines represent hydrogen bonds, grey semi-circles denote hydrophobic interactions made by TUBG1 and Pink semi-circles represent hydrophobic interactions made by the orf3a.

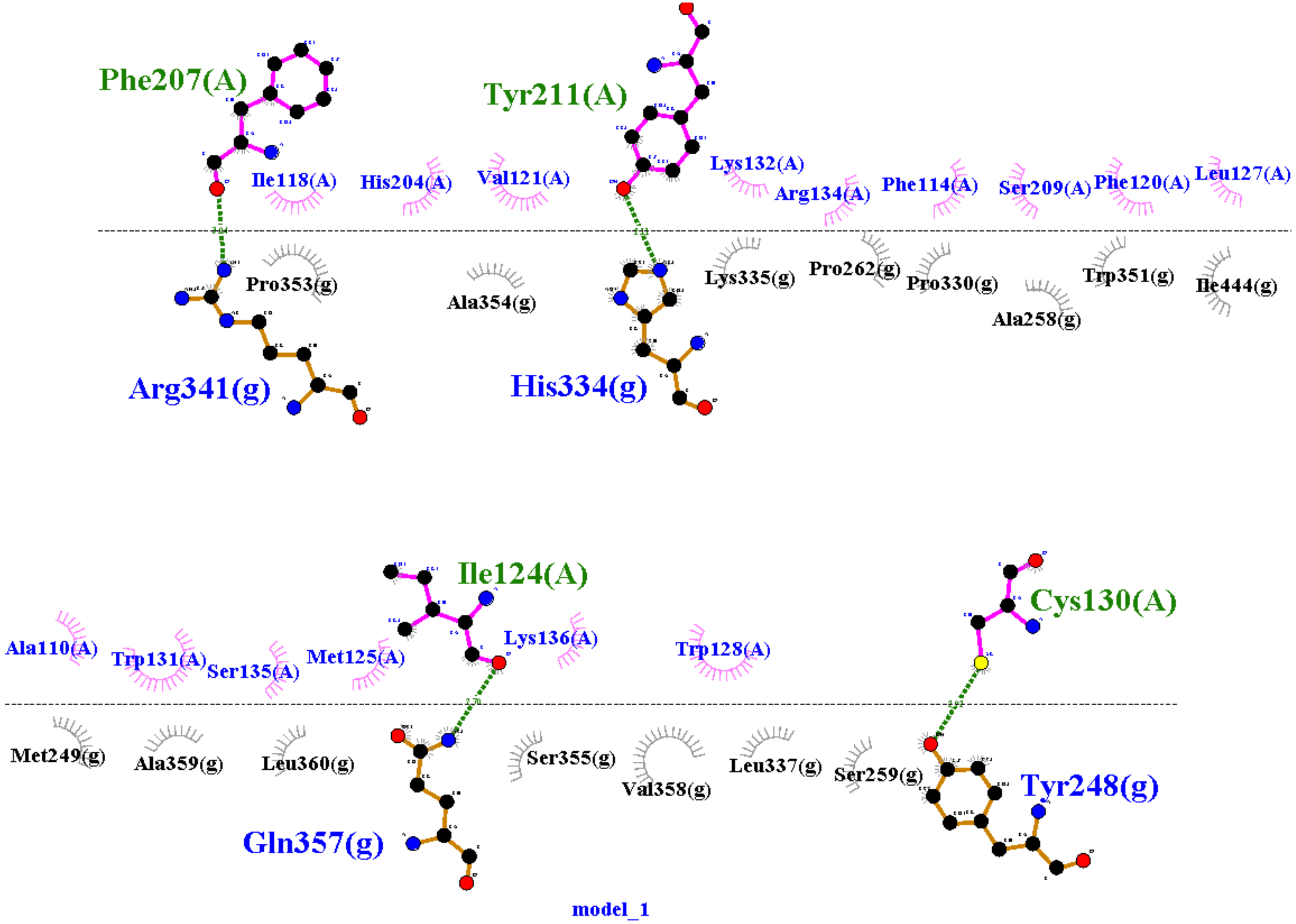

Fig S54: 2D representation of interacting residues between TUBG1 (At TUBG1 residues that interact with GCP3) (Chain g) and orf3a (Chain A), Complex 1,provided by the LIGPLOT v.2.2 software. Olive Green lines represent hydrogen bonds, grey semi-circles denote hydrophobic interactions made by TUBG1 and Pink semi-circles represent hydrophobic interactions made by the orf3a.

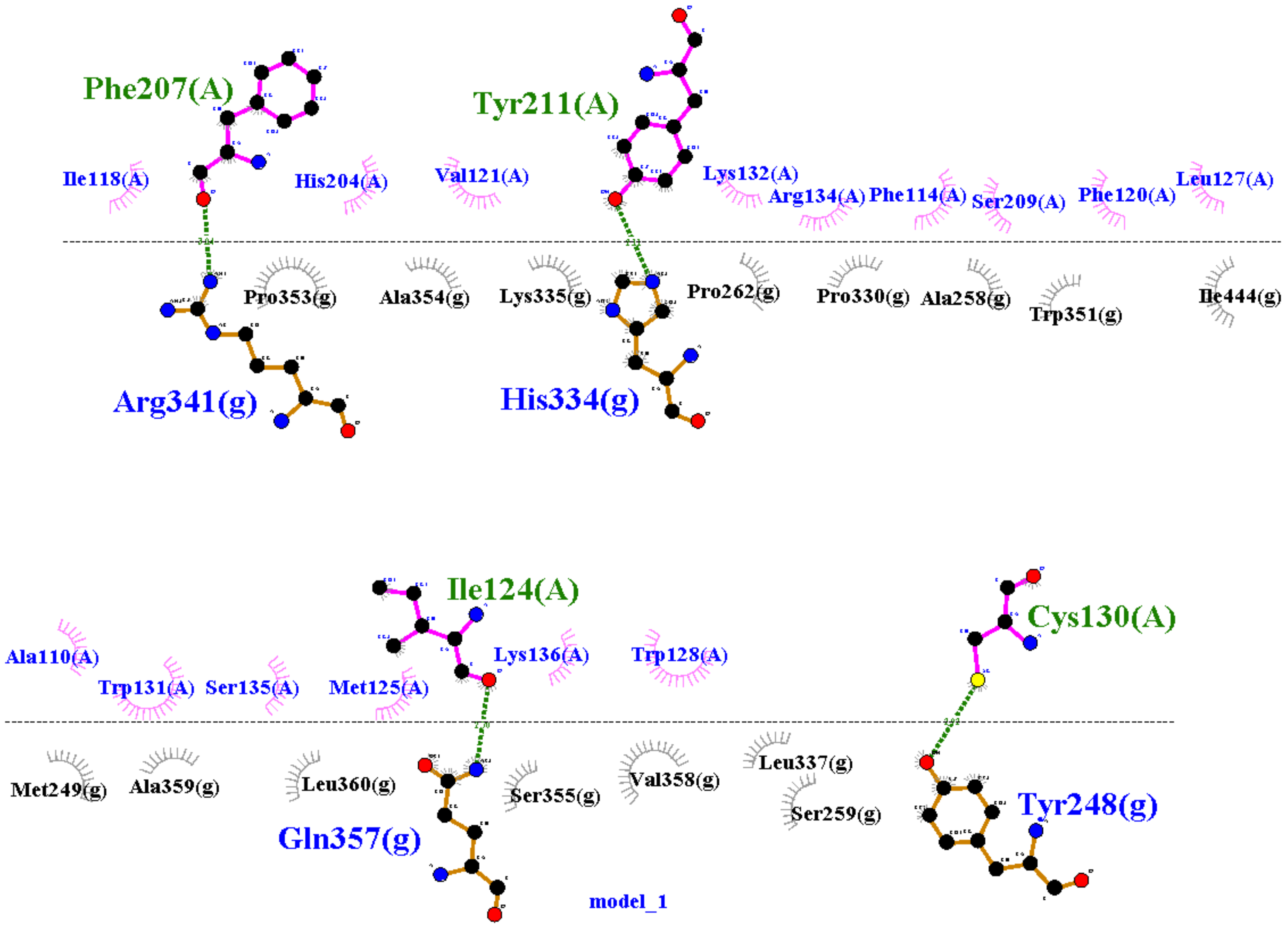

Fig S55: 2D representation of interacting residues between TUBG1 (At TUBG1 residues that interact with GCP3) (Chain g) and orf3a (Chain A), Complex 3,provided by the LIGPLOT v.2.2 software. Olive Green lines represent hydrogen bonds, grey semi-circles denote hydrophobic interactions made by TUBG1 and Pink semi-circles represent hydrophobic interactions made by the orf3a.

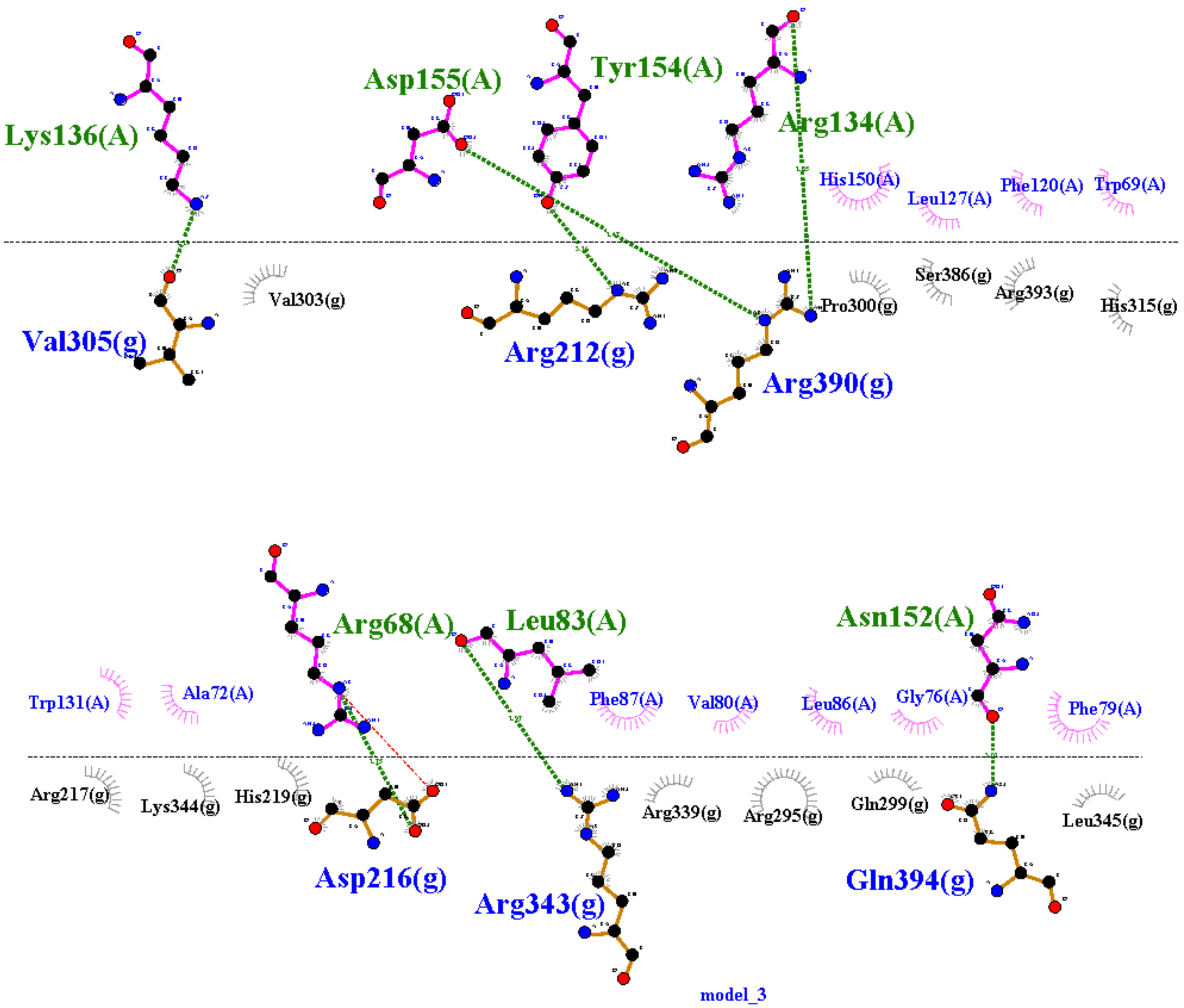

Fig S56: 2D representation of interacting residues between LMP-1 (Chain Y) and **3CLpro/Mpro** (Chain A) complex 1, provided by the LIGPLOT v.2.2 software. Olive Green lines represent hydrogen bonds, grey semi-circles denote hydrophobic interactions made by **3CLpro/Mpro** and Pink semi-circles represent hydrophobic interactions made by the LMP1.

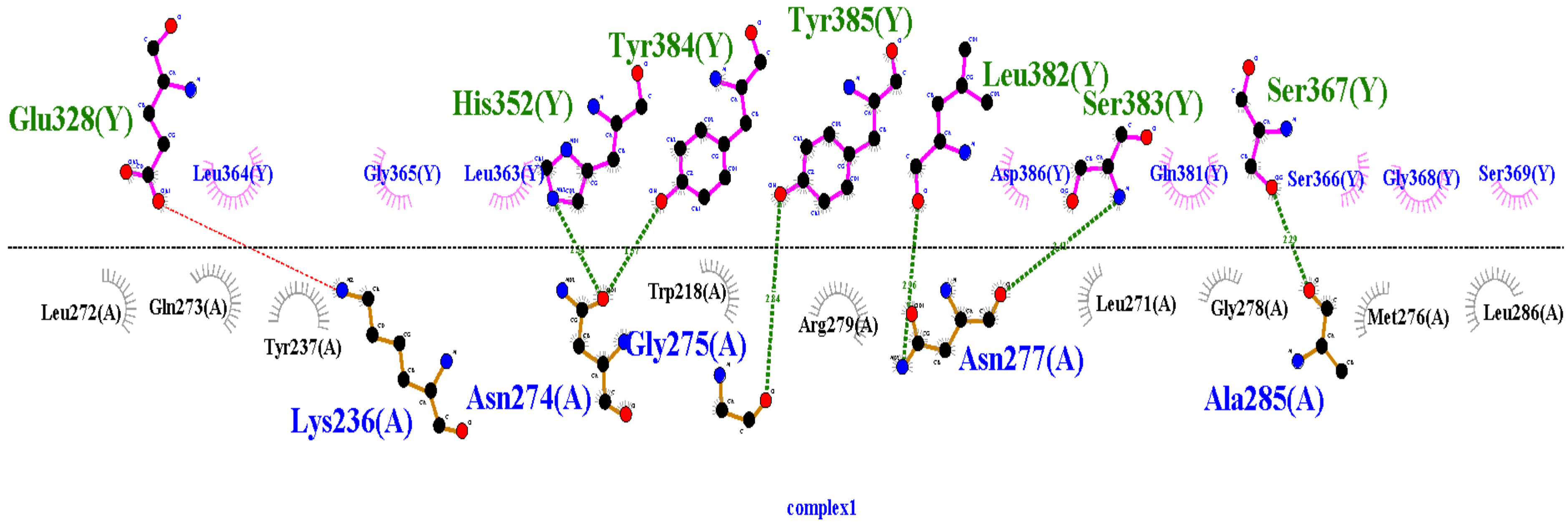

Fig S57: 2D representation of interacting residues between LMP-1 (Chain Y) and **3CLpro/Mpro** (Chain A) complex 9, provided by the LIGPLOT v.2.2 software. Olive Green lines represent hydrogen bonds, grey semi-circles denote hydrophobic interactions made by **3CLpro/Mpro** and Pink semi-circles represent hydrophobic interactions made by the LMP1.

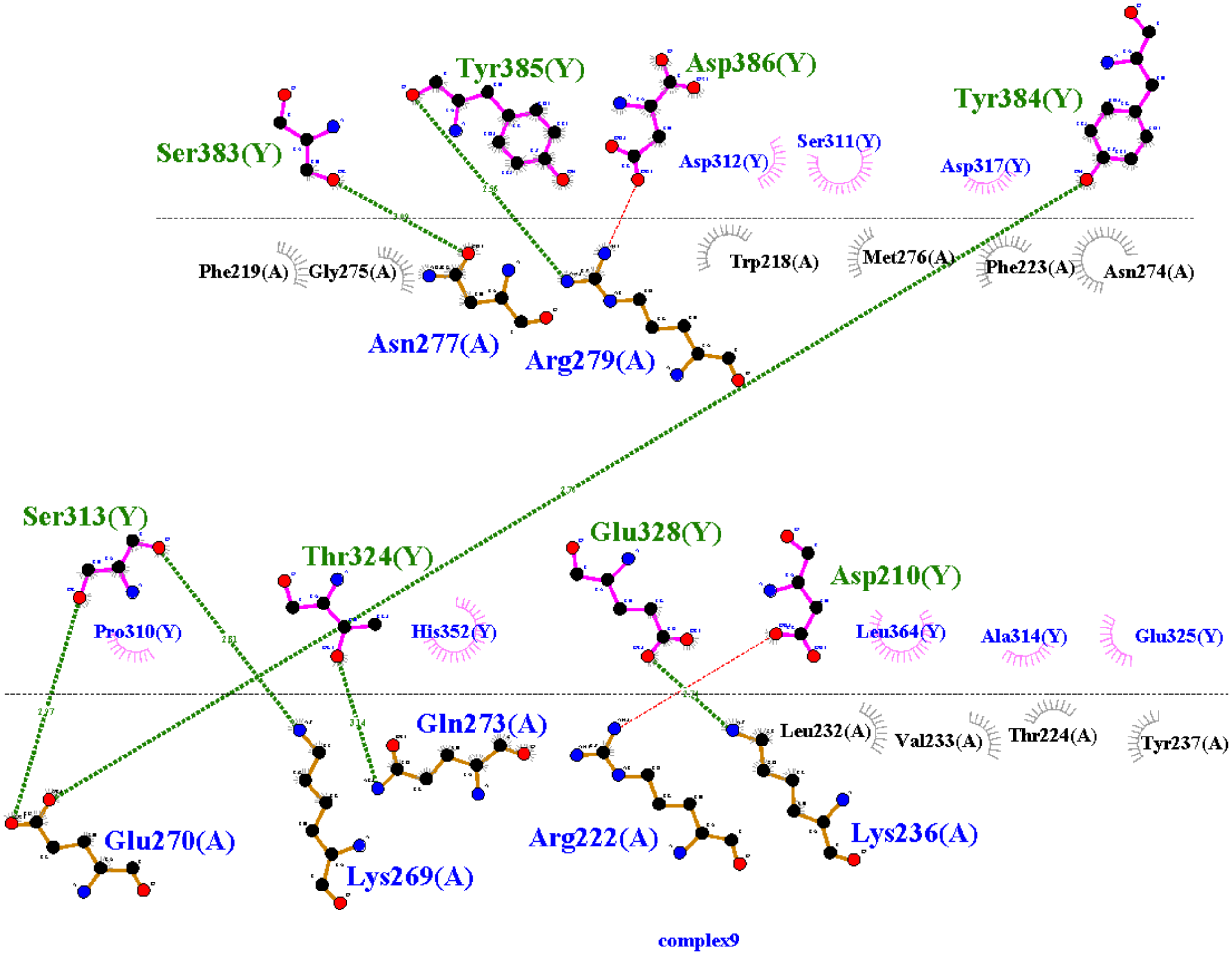

Fig S58: 2D representation of interacting residues between LMP-1 (Chain Y) and **3CLpro/Mpro** (Chain A) complex 10, provided by the LIGPLOT v.2.2 software. Olive Green lines represent hydrogen bonds, grey semi-circles denote hydrophobic interactions made by **3CLpro/Mpro** and Pink semi-circles represent hydrophobic interactions made by the LMP1.

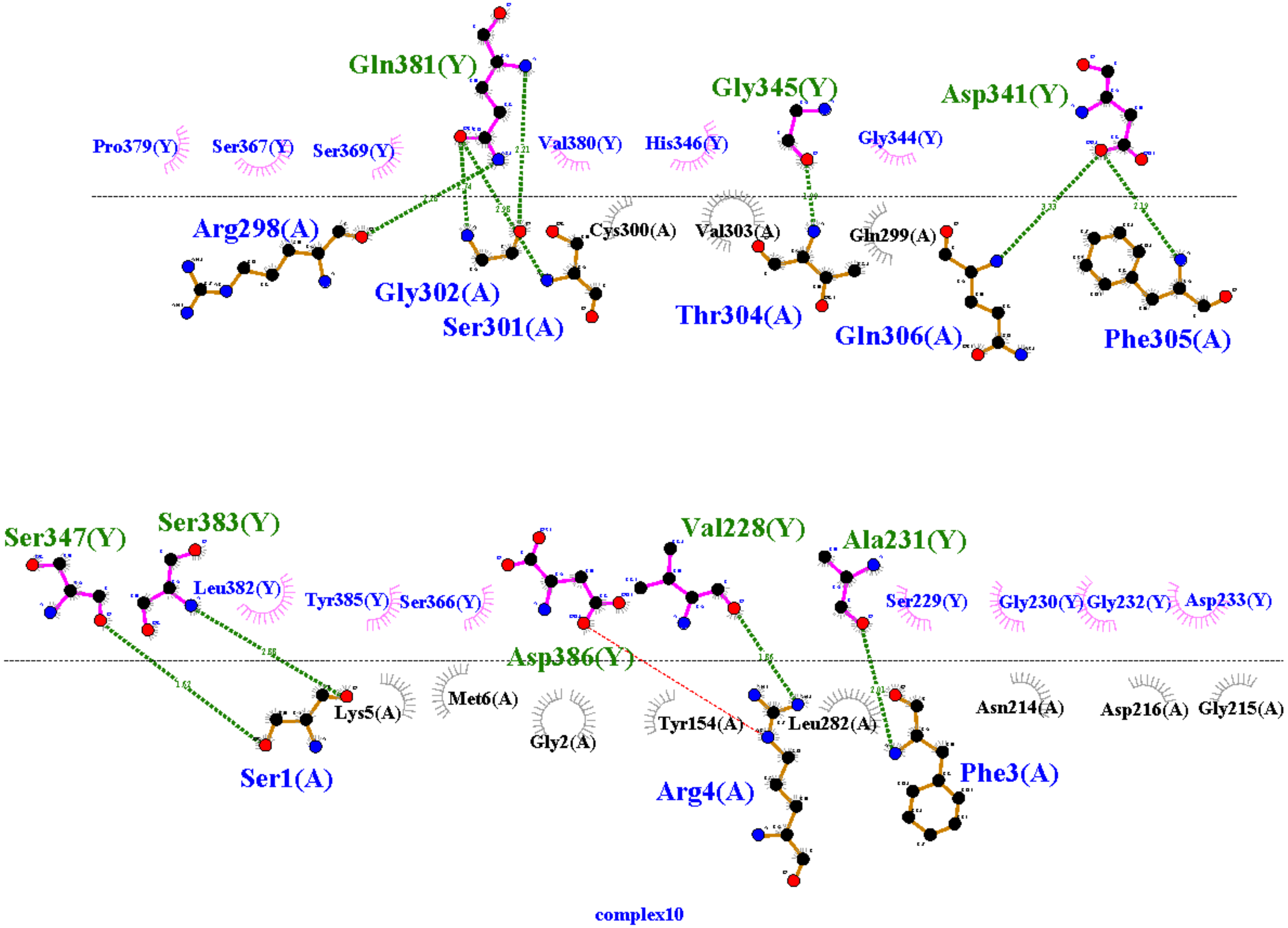

Fig S59: 2D representation of interacting residues between LMP-1 (Chain Y) and nsp7 (Chain A) complex 6, provided by the LIGPLOT v.2.2 software. Olive Green lines represent hydrogen bonds, grey semi-circles denote hydrophobic interactions made by LPM1 and Pink semi-circles represent hydrophobic interactions made by the nsp7.

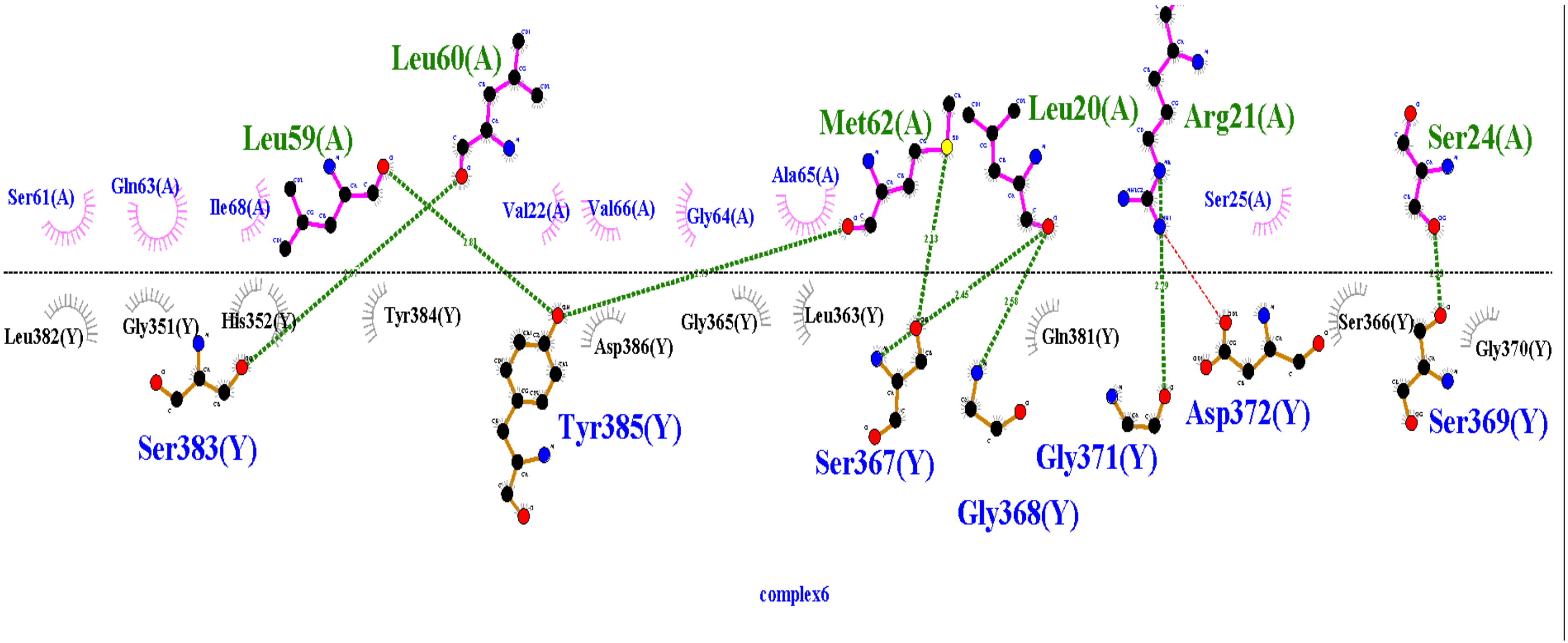

Fig S60: 2D representation of interacting residues between LMP-1 (Chain Y) and nsp7 (Chain A) complex 7, provided by the LIGPLOT v.2.2 software. Olive Green lines represent hydrogen bonds, grey semi-circles denote hydrophobic interactions made by LPM1 and Pink semi-circles represent hydrophobic interactions made by the nsp7.

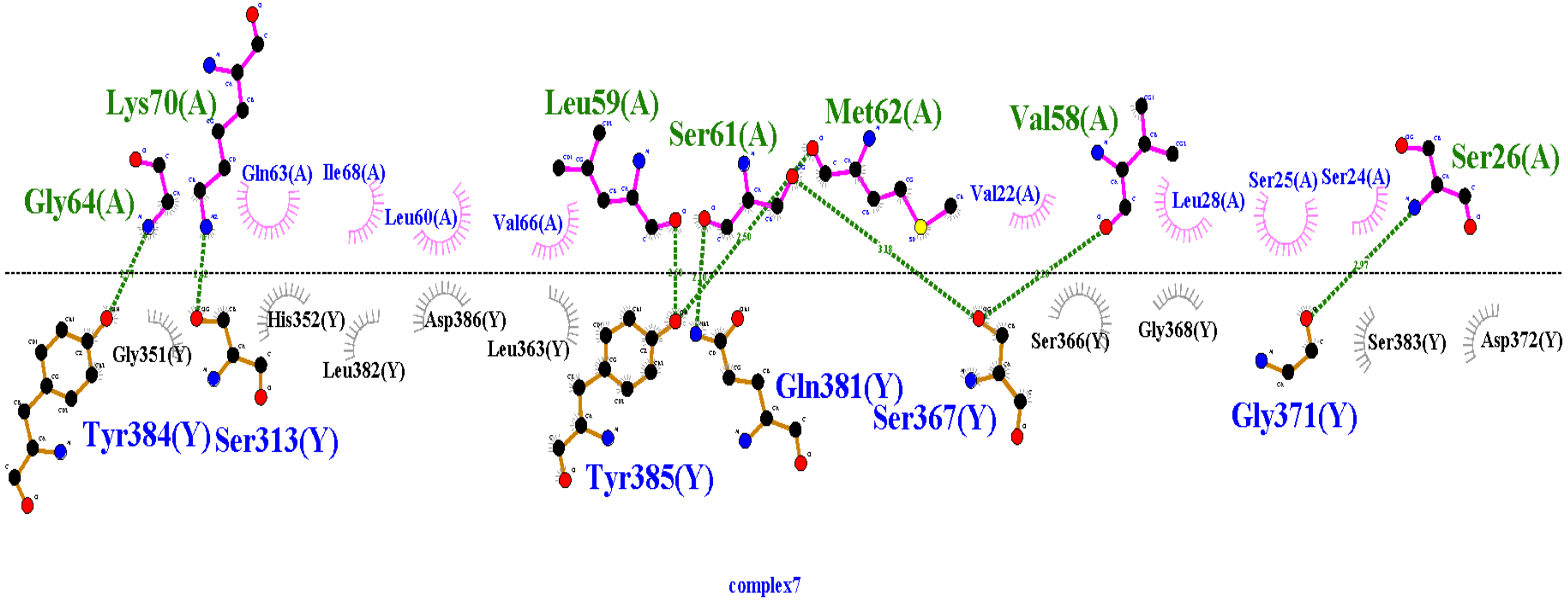

Fig S61: 2D representation of interacting residues between LMP-1 (Chain Y) and nsp7 (Chain A) complex 8, provided by the LIGPLOT v.2.2 software. Olive Green lines represent hydrogen bonds, grey semi-circles denote hydrophobic interactions made by LPM1 and Pink semi-circles represent hydrophobic interactions made by the nsp7.

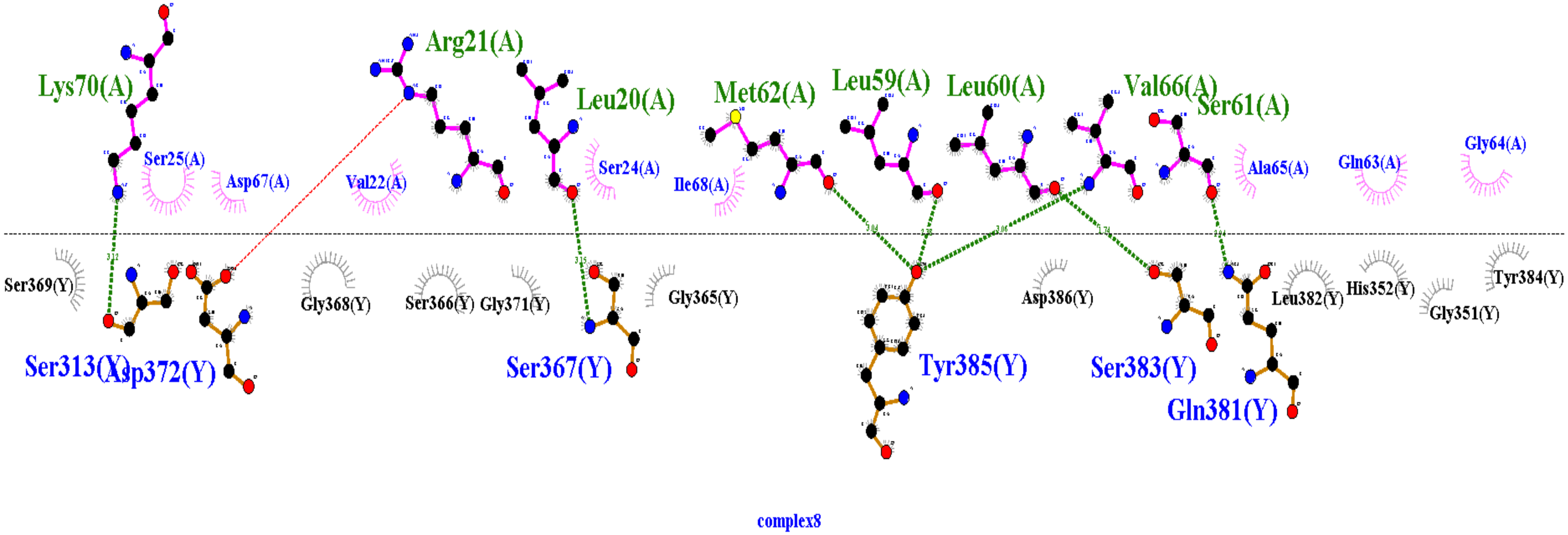

Fig S62: 2D representation of interacting residues between LMP-1 (Chain Y) and nsp10 (Chain B) complex 2, provided by the LIGPLOT v.2.2 software. Olive Green lines represent hydrogen bonds, grey semi-circles denote hydrophobic interactions made by LPM1 and Pink semi-circles represent hydrophobic interactions made by the nsp10.

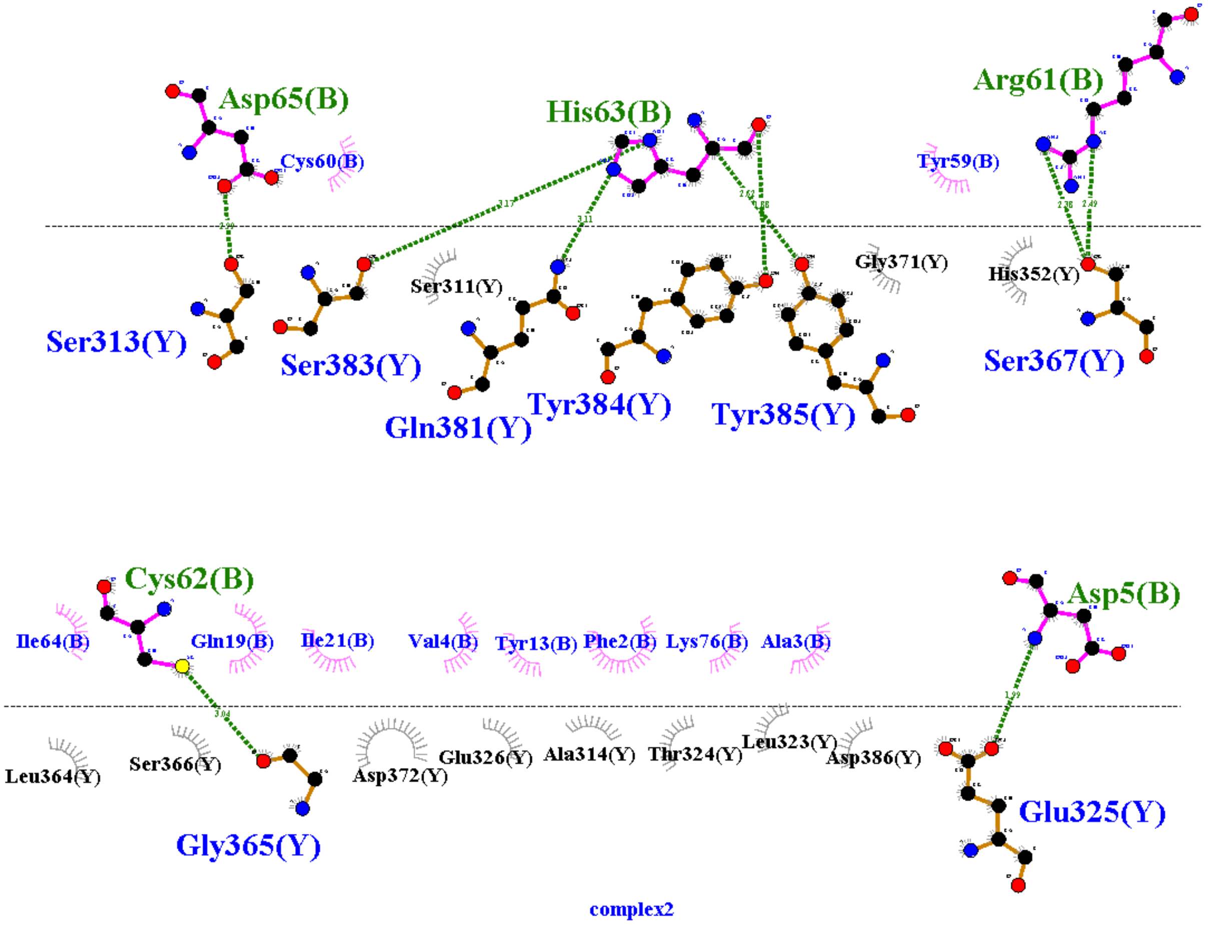

Fig S63: 2D representation of interacting residues between LMP-1 (Chain Y) and nsp10 (Chain B) complex 8, provided by the LIGPLOT v.2.2 software. Olive Green lines represent hydrogen bonds, grey semi-circles denote hydrophobic interactions made by LPM1 and Pink semi-circles represent hydrophobic interactions made by the nsp10.

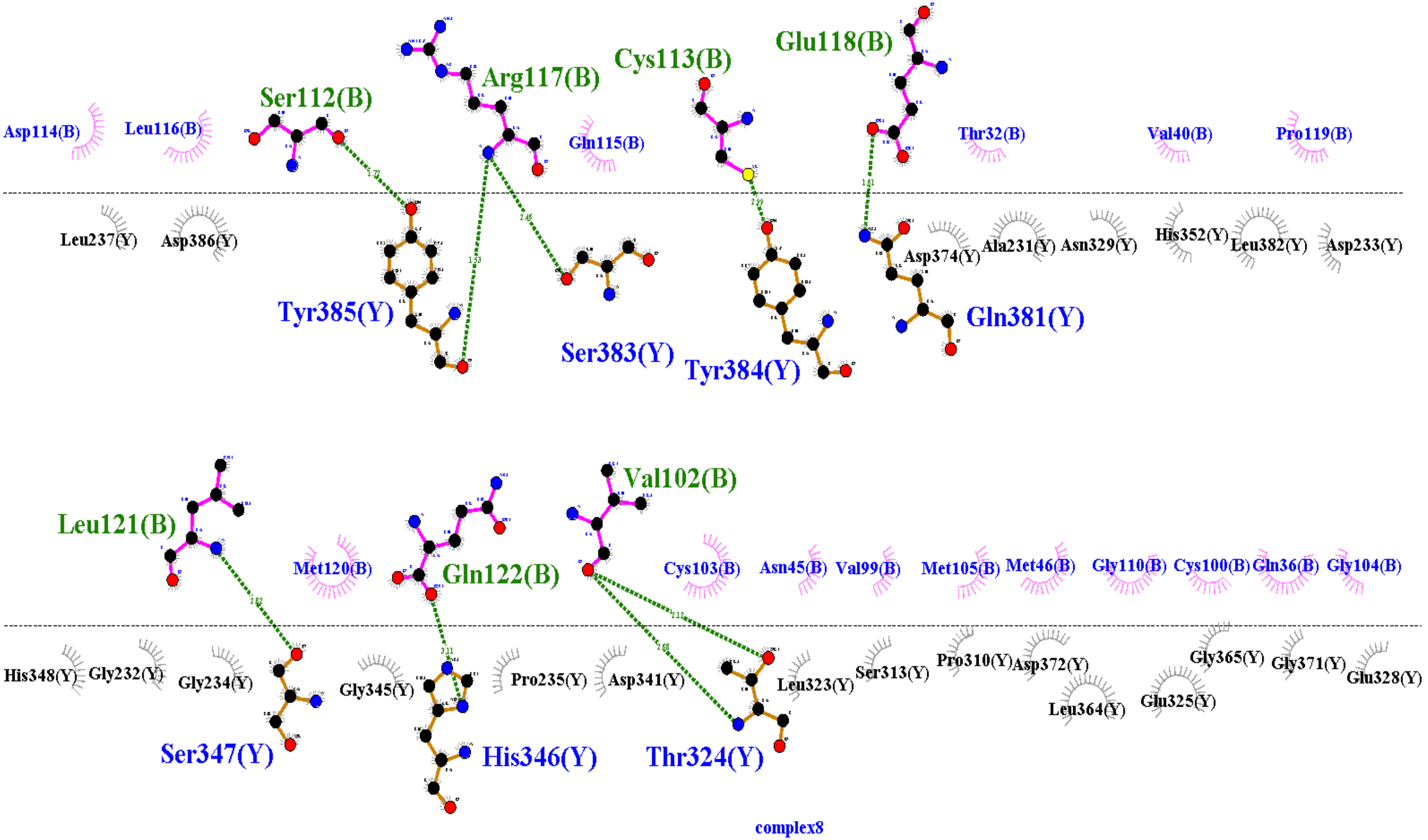

Fig S64: 2D representation of interacting residues between LMP-1 (Chain Y) and CoV-2 Spike (Chain B) complex 6, provided by the LIGPLOT v.2.2 software. Olive Green lines represent hydrogen bonds, grey semi-circles denote hydrophobic interactions made by CoV-2 Spike and Pink semi-circles represent hydrophobic interactions made by the LMP1.

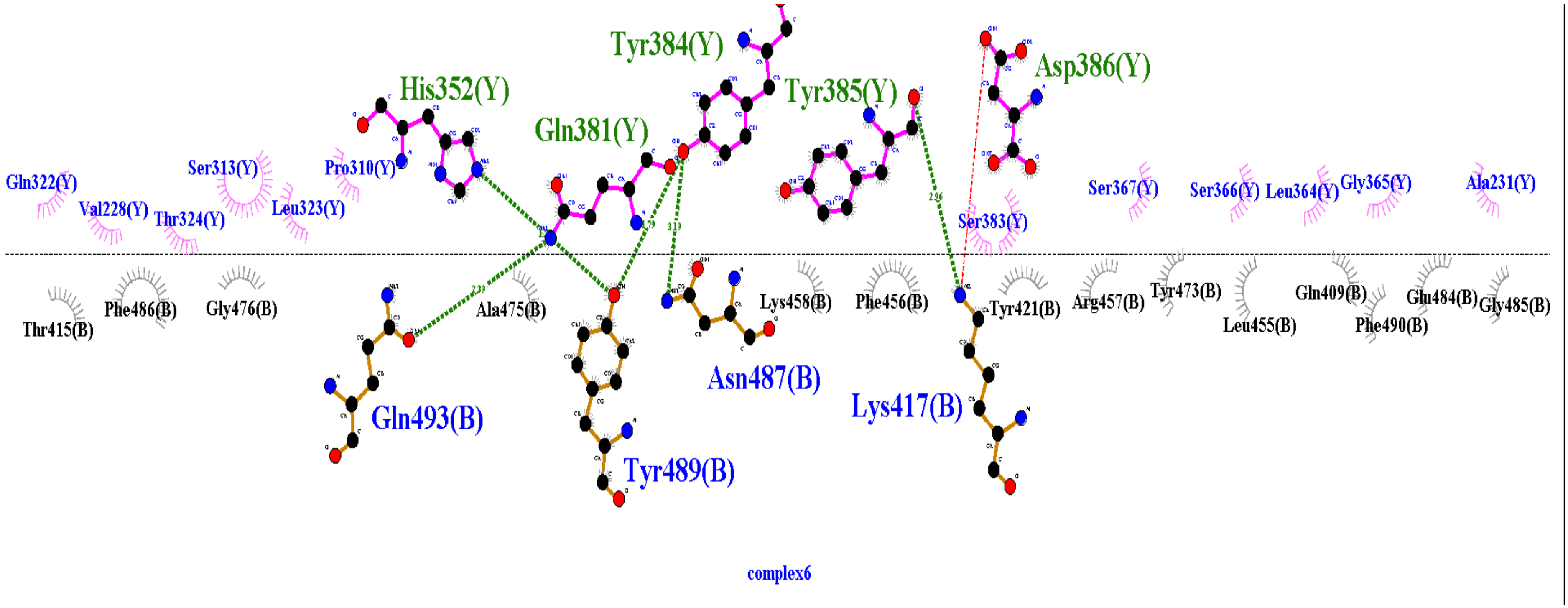

Fig S65: 2D representation of interacting residues between LMP-1 (Chain Y) and CoV-2 Spike (Chain B) complex 7, provided by the LIGPLOT v.2.2 software. Olive Green lines represent hydrogen bonds, grey semi-circles denote hydrophobic interactions made by CoV-2 Spike and Pink semi-circles represent hydrophobic interactions made by the LMP1.

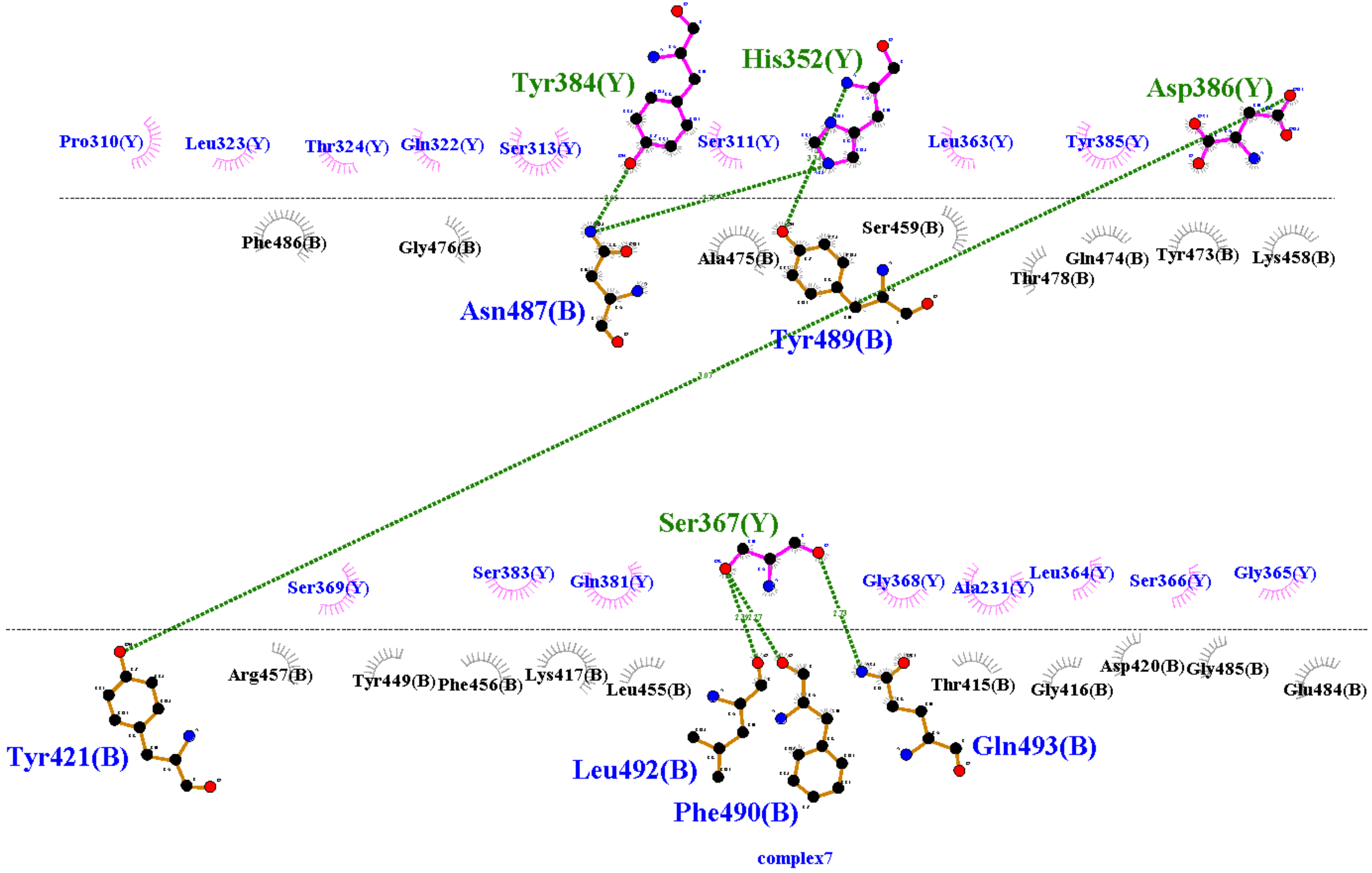

Fig S66: 2D representation of interacting residues between LMP-1 (Chain Y) and CoV-2 Spike (Chain B) complex 8, provided by the LIGPLOT v.2.2 software. Olive Green lines represent hydrogen bonds, grey semi-circles denote hydrophobic interactions made by CoV-2 Spike and Pink semi-circles represent hydrophobic interactions made by the LMP1.

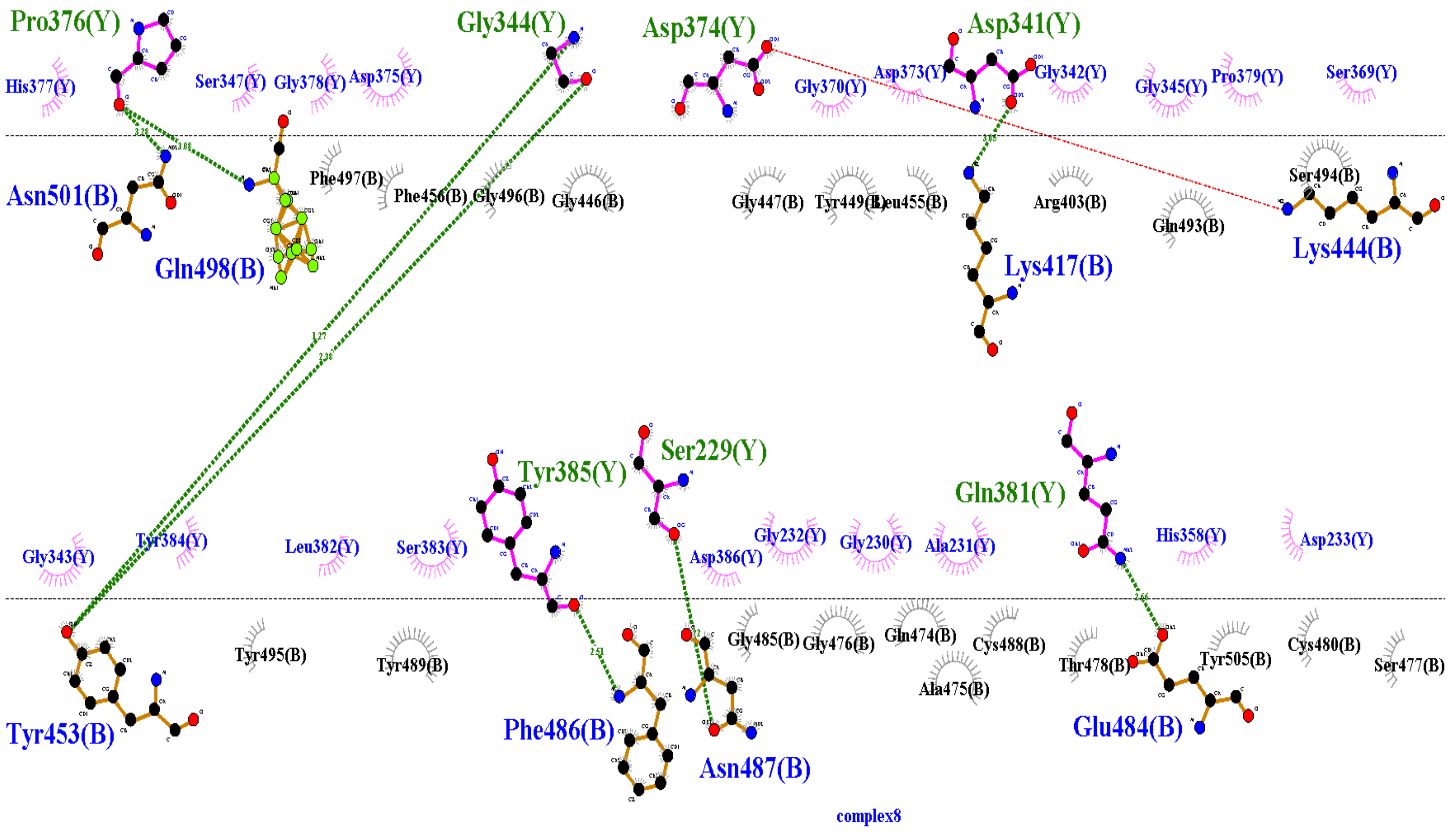

Fig S67: 2D representation of interacting residues between PD-1 (Chain G) and CoV-2 Spike (Chain B) complex 2, provided by the LIGPLOT v.2.2 software. Olive Green lines represent hydrogen bonds, grey semi-circles denote hydrophobic interactions made by CoV-2 Spike and Pink semi-circles represent hydrophobic interactions made by the **PD-1**.

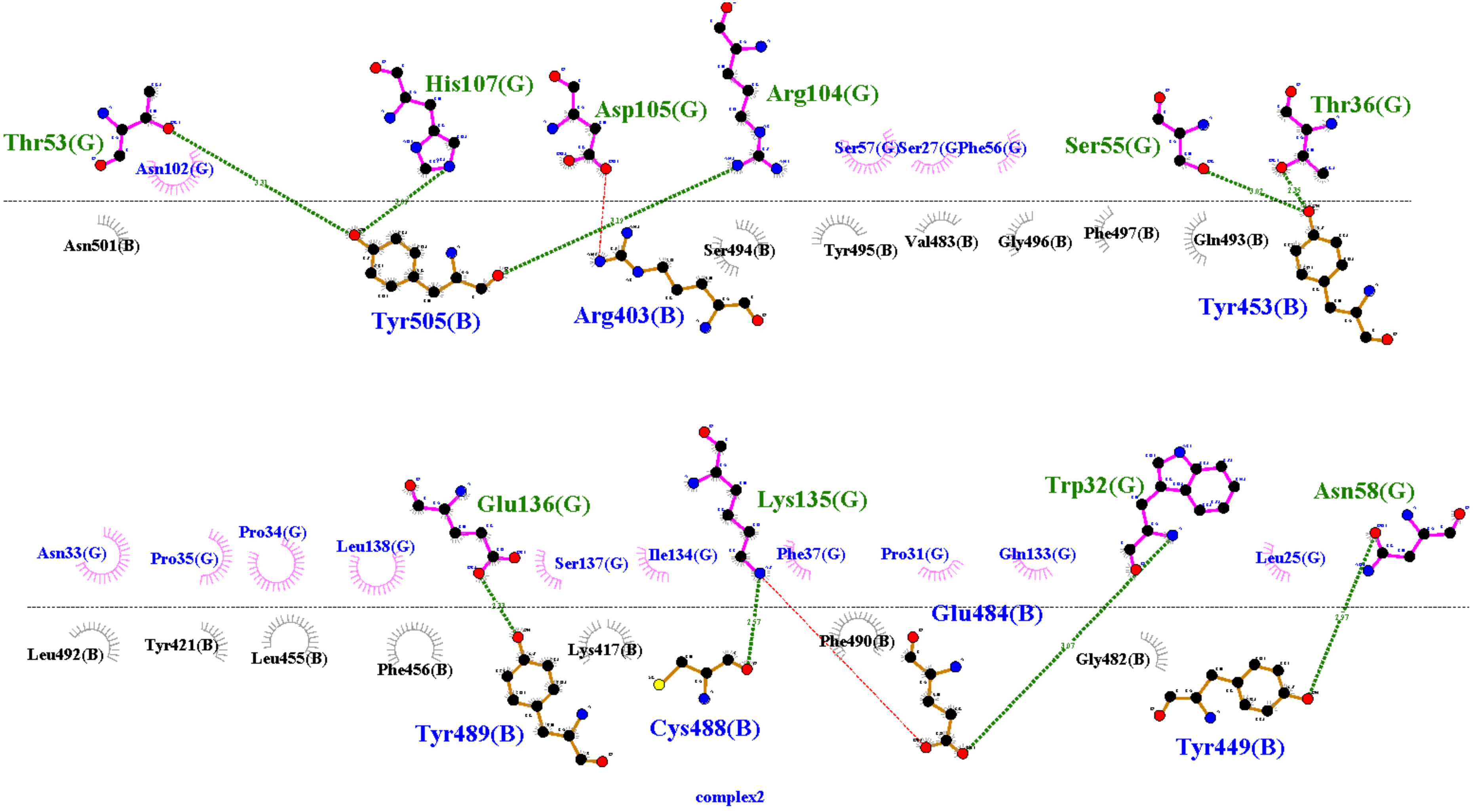

Fig S68: 2D representation of interacting residues between PD-1 (Chain G) and CoV-2 Spike (Chain B) complex 7, provided by the LIGPLOT v.2.2 software. Olive Green lines represent hydrogen bonds, grey semi-circles denote hydrophobic interactions made by CoV-2 Spike and Pink semi-circles represent hydrophobic interactions made by the **PD-1**..

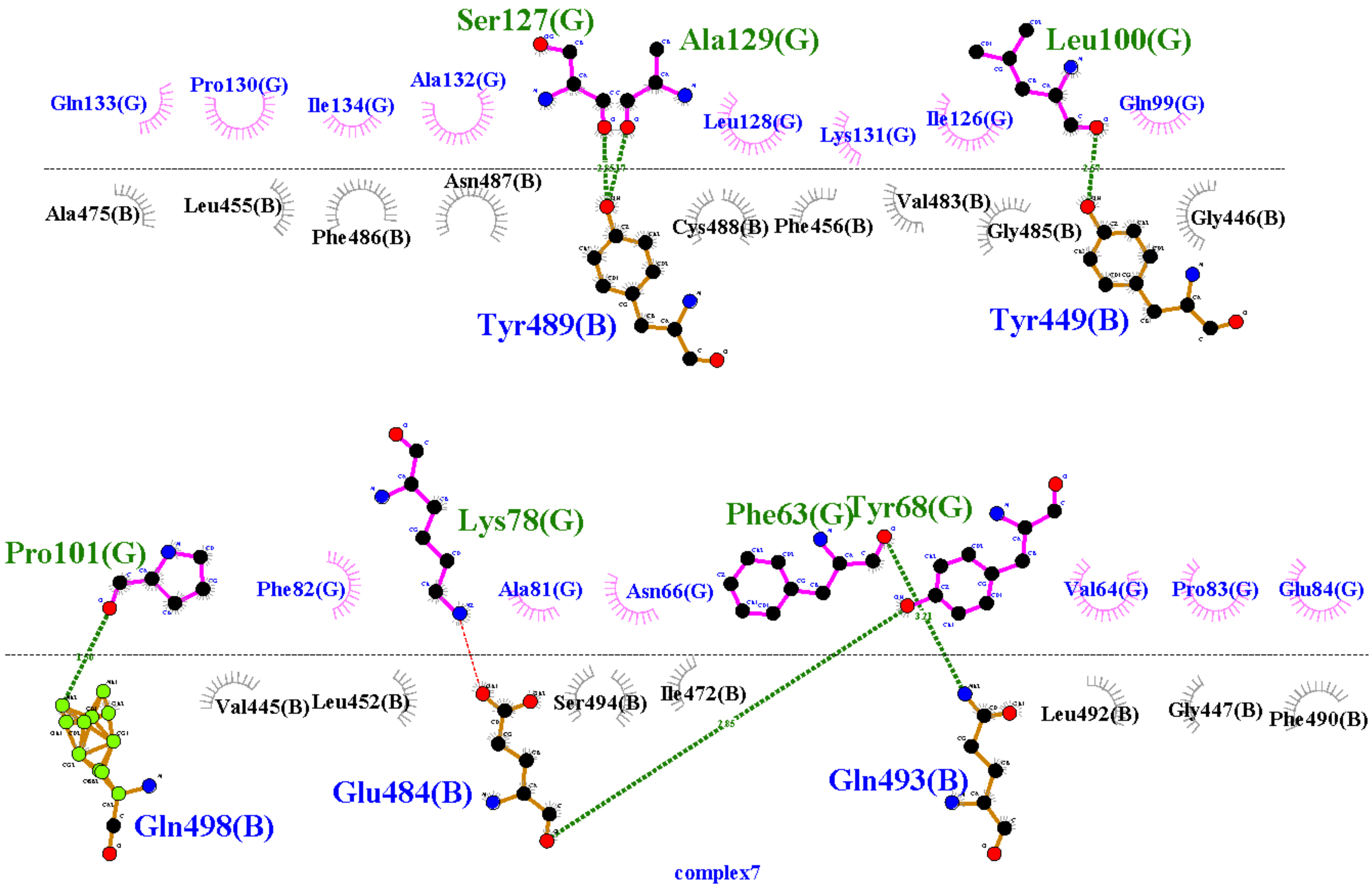

Fig S69: 2D representation of interacting residues between PD-1 (Chain G) and CoV-2 Spike (Chain B) complex 8, provided by the LIGPLOT v.2.2 software. Olive Green lines represent hydrogen bonds, grey semi-circles denote hydrophobic interactions made by CoV-2 Spike and Pink semi-circles represent hydrophobic interactions made by the **PD-1**.

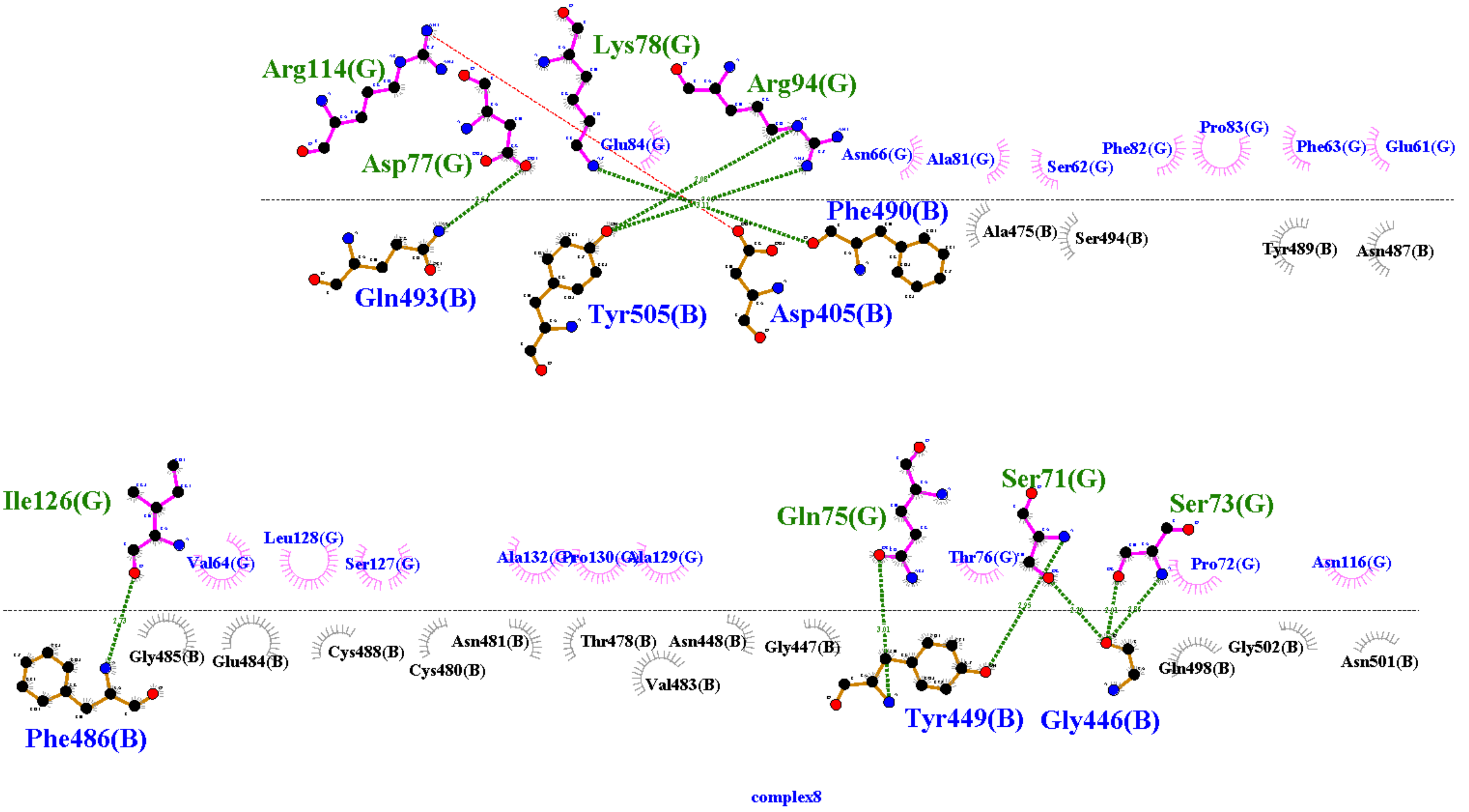

Supplement: Supplementary file 1 [file viruses-13-01927-s001.zip › Suppl Figs_S1-S69_20-7-21.pdf]
