# Supplementary material for: Potential Molecular Mechanisms of Rare Anti-Tumor Immune Response by SARS-CoV-2 in Isolated Cases of Lymphomas
Source: Viruses. 2021 Sep 25;13(10):1927. doi: 10.3390/v13101927 (PMC8539762; doi:10.3390/v13101927)

>sp|P28908|TNR8_HUMAN Tumor necrosis factor receptor superfamily member 8
OS=Homo sapiens OX=9606 GN=TNFRSF8 PE=1 SV=1
MRVLLAALGLLFLGALRAFPQDRPFEDTCHGNPSHYYDKAVRRCCYRCPMGLFPTQQCPQRPTDCRKQCEPDYYLDE
ADRCTACVTCSRDDLVEKTPCAWNSSRVCECRPGMFCSTSAVNSCARCFFHSVCPAGMIVKFPGTAQKNTVCEPASP
GVSPACASPENCKEPSSGTIPQAKPTPVSPATSSASTMPVRGGTRLAQEAASKLTRAPDSPSSVGRPSSDPGLSPTQ
PCPEGSGDCRKQCEPDYYLDEAGRCTACVSCSRDDLVEKTPCAWNSSRTCECRPGMICATSATNSCARCVPYPICAA
ETVTKPQDMAEKDTTFEAPPLGTQPDCNPTPENGEAPASTSPTQSLLVDSQASKTLPIPTSAPVALSSTGKPVLDAG
PVLFWVILVLVVVVGSSAFLLCHRRACRKRIRQKLHLCYPVQTSQPKLELVDSRPRRSSTQLRSGASVTEPVAEERG
LMSQPLMETCHSVGAAYLESLPLQDASPAGGPSSPRDLPEPRVSTEHTNNKIEKIYIMKADTVIVGTVKAELPEGRG
LAGPAEPELEEELEADHTPHYPEQETEPPLGSCSDVMLSVEEEGKEDPLPTAASGK

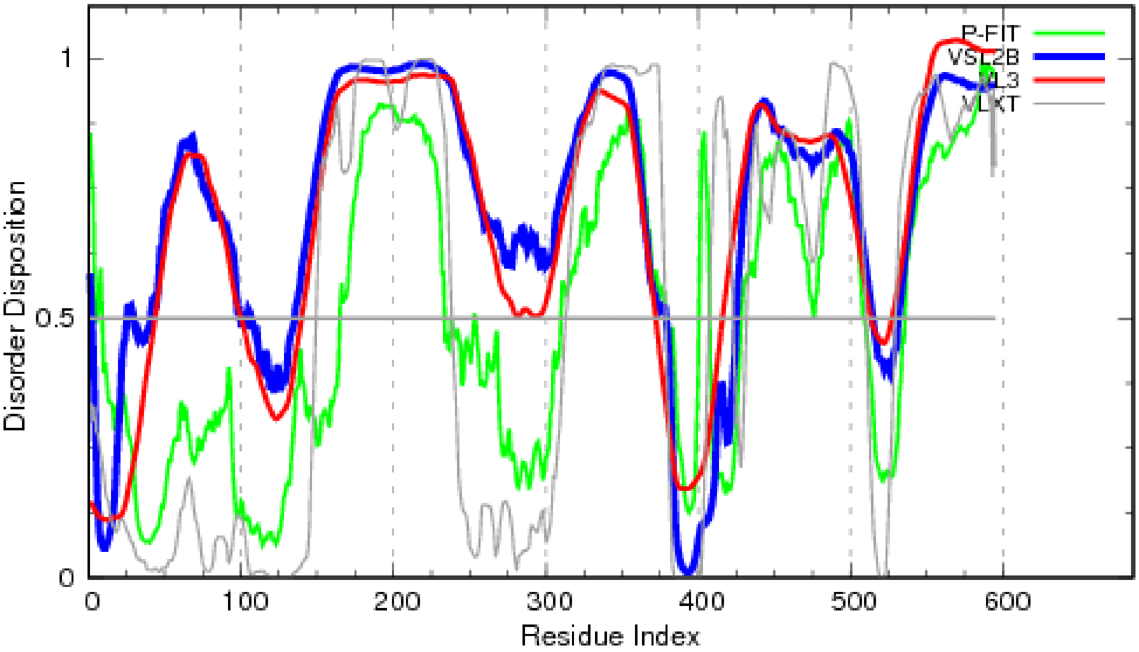

>sp|P22083|FUT4_HUMAN Alpha-(1,3)-fucosyltransferase 4 OS=Homo sapiens OX=9606 GN=FUT4 PE=2 SV=3

```
MRRLWGAARKPSGAGWEKEWAEAPQEAPGAWSGRLGPGRSGRKGRAVPGWASWPAHLALAARPARHLGGAGQGPRPL
HSGTAPFHSRASGERQRRLEPQLQHESRCRSSTPADAWRAEAALPVRAMGAPWGSPTAAAGGRRGWRRGRGLPWTVC
VLAAAGLTCTALITYACWGQLPPLPWASPTPSRPVGVLLWWEPFGGRDSAPRPPPDCRLRFNISGCRLLTDRASYGE
AQAVLFHHRDLVKGPPDWPPPWGIQAHTAEEVDLRVLDYEEAAAAAEALATSSPRPPGQRWVWMNFESPSHSPGLRS
LASNLFNWTLSYRADSDVFVPYGYLYPRSHPGDPPSGLAPPLSRKQGLVAWVVSHWDERQARVRYYHQLSQHVTVDV
FGRGGPGQPVPEIGLLHTVARYKFYLAFENSQHLDYITEKLWRNALLAGAVPVVLGPDRANYERFVPRGAFIHVDDF
PSASSLASYLLFLDRNPAVYRRYFHWRRSYAVHITSFWDEPWCRVCQAVQRAGDRPKSIRNLASWFER
```

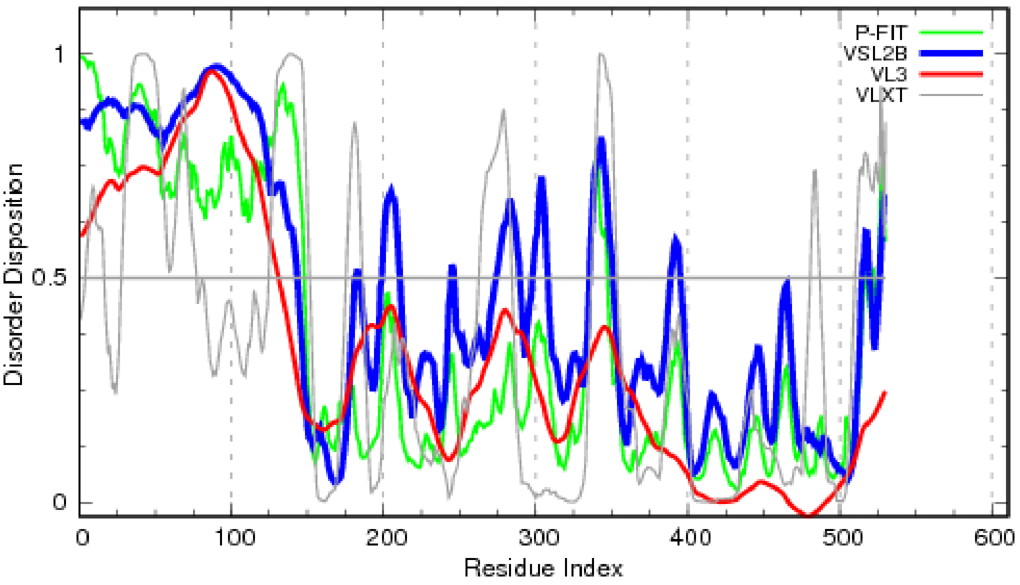

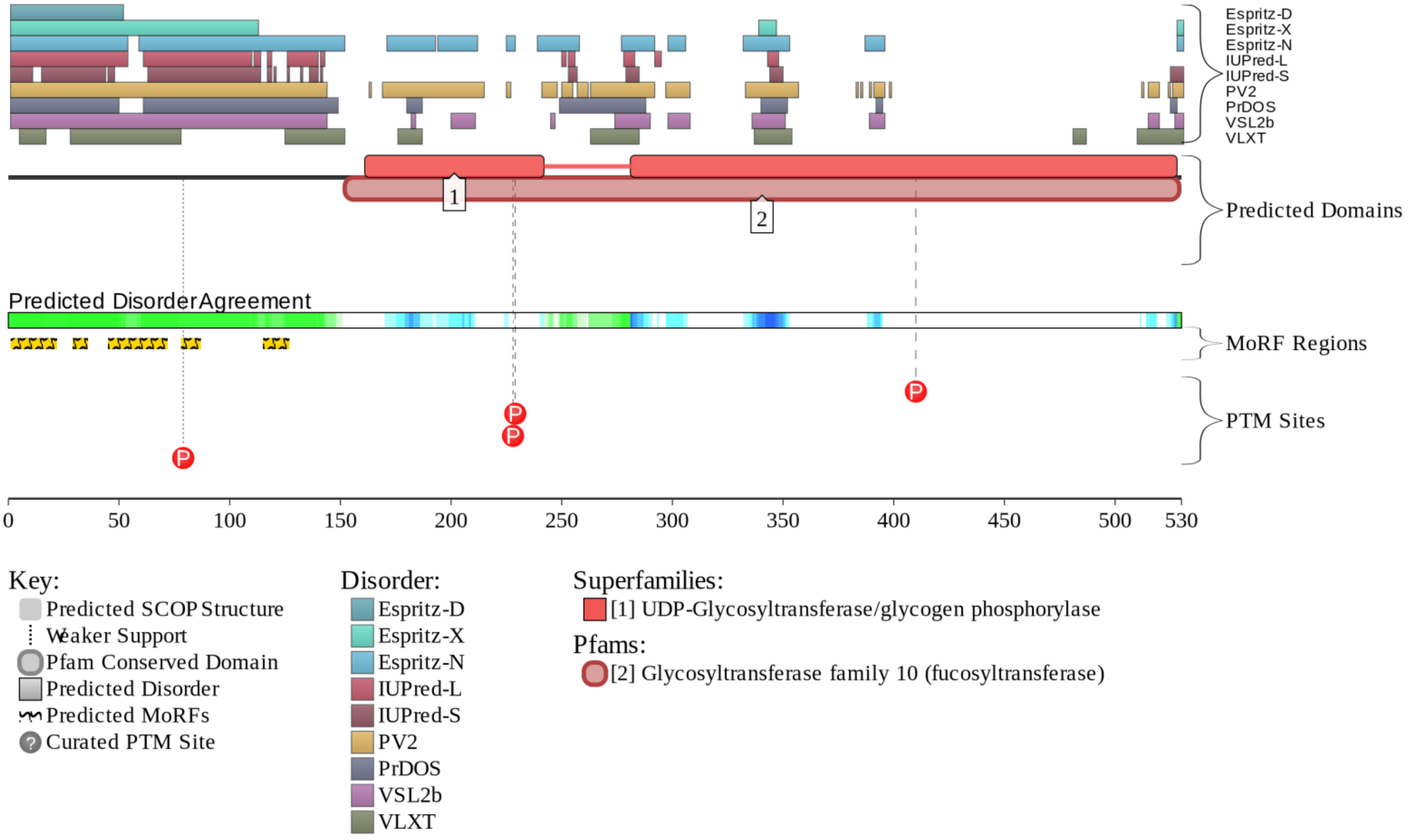

```
>sp|P03230|LMP1_EBVB9 Latent membrane protein 1 OS=Epstein-Barr virus (strain
B95-8) OX=10377 GN=LMP1 PE=1 SV=1
MEHDLERGPPGPRRPPRGPPLSSSLGLALLLLLLALLFWLYIVMSDWTGGALLVLYSFALMLIIIILIIFIFRRDLL
CPLGALCILLLMITLLLIALWNLHGQALFLGIVLFIFGCLLVLGIWIYLLEMLWRLGATIWQLLAFFLAFFLDLILL
IIALYLQQNWWTLLVDLLWLLLFLAILIWMYYHGQRHSDEHHHDDSLPHPQQATDDSGHESDSNSNEGRHHLLVSGA
GDGPPLCSQNLGAPGGGPDNGPQDPDNTDDNGPQDPDNTDDNGPHDPLPQDPDNTDDNGPQDPDNTDDNGPHDPLPH
SPSDSAGNDGGPPQLTEEVENKGGDQGPPLMTDGGGGHSHDSGHGGGDPHLPTLLLGSSGSGGDDDDPHGPVQLSYY
D
```

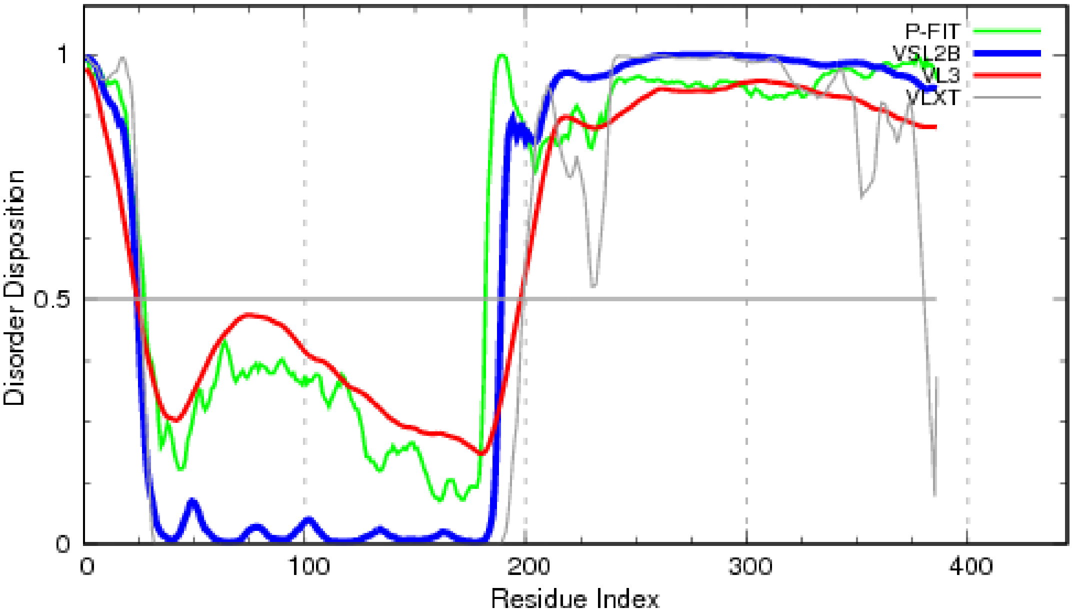


```
>sp|P0DTC5|VME1_SARS2 Membrane protein OS=Severe acute respiratory syndrome
coronavirus 2 OX=2697049 GN=M PE=1 SV=1
MADSNGTITVEELKKLLEQWNLVIGFLFLTWICLLQFAYANRNRFLYIIKLIFLWLLWPVTLACFVLAAVYRINWIT
GGIAIAMACLVGLMWLSYFIASFRLFARTRSMWSFNPETNILLNVPLHGTILTRPLLESELVIGAVILRGHLRIAGH
HLGRCDIKDLPKEITVATSRTLSYYKLGASQRVAGDSGFAAYSRYRIGNYKLNTDHSSSSDNIALLVQ
```

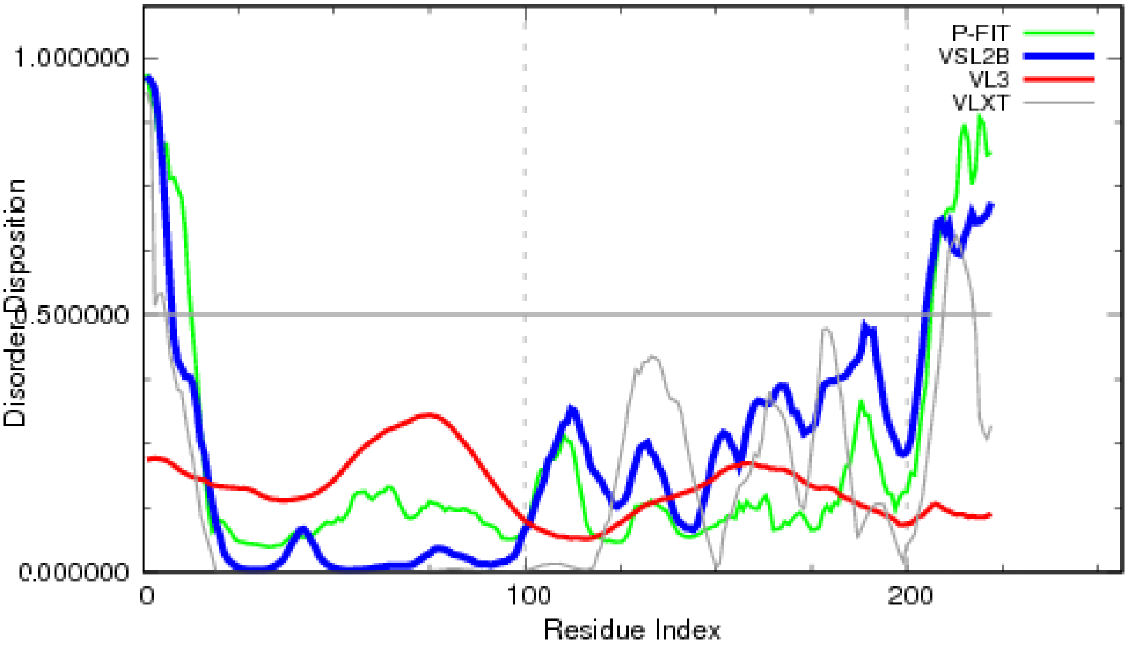

Supplement: Supplementary file 1 [file viruses-13-01927-s001.zip › Suppl File_S1_20-7-21.pdf]
